# Contactless medical equipment AI big data risk control and quasi thinking iterative planning

**DOI:** 10.1038/s41598-022-18724-5

**Published:** 2022-09-03

**Authors:** Zhu Rongrong

**Affiliations:** Fudan University, Shanghai, China

**Keywords:** Applied mathematics, Scientific data, Statistics

## Abstract

Research Background, the intelligent polymorphic system of heavy core clustering fitting iterative programming is constructed by using the edge lens of dual core heavy core. The tracking system of heavy core TANH equilibrium array is used to obtain the abnormal data range. The energy regular fluctuation of the edge lens with dual core and heavy core is used to obtain high-definition images. And build the complexity dependent parameter group from low-end equipment to high-end equipment. Heavy core clustering of hierarchical fuzzy clustering system based on differential incremental balance theory is applied to Contactless medical equipment AI big data risk control and quasi thinking iterative planning. At the same time, the mathematical model risk control is performed by fitting the TANH balance of the local nonlinear random regular micro-vibration diffusion curve. The CT/MR original data is subjected to hierarchical cross domain overlapping grid screening with the structure of fitting weakly nonlinear curve, which can capture the heavy core cluster analysis of the core layer of big data anomalies [1:10]. Successfully control the parameter group of CT/MR machine internal data, big data AI Mathematical model risk. The polar graph of high-dimensional heavy core clustering processing data is regular and scientific. The same time, it can prevent the dimension disaster caused by the construction of high-dimensional big data due to the partial loss of original data, and form a stable and predictable maintenance of CT/MR. Compared with the discrete characteristics of the polar graph of the original data. So as to correctly detect and control the dynamic change process of CT/MR in the entire life cycle. It provides help for predictive maintenance of early pre-inspection and orderly maintenance of the medical system, and developed standardized model software of automated unsupervised learning for medical big equipment big data AI Mathematical model risk control. Scientifically evaluated the exposure time and heat capacity MHU% of CT tubes, as well as the internal law of MR (nuclear magnetic resonance), and processed big data twice and three times in heavy nuclear clustering. After optimizing the algorithm, hundreds of thousands of nonlinear random vibrations are performed in the operation and maintenance database every second, and at least 30 concurrent operations are formed, which greatly improves and shortens the operation time (Yanwei et al. in J Complex 2017:1–9, 2017. 10.1155/2017/3437854). Finally, after adding micro-vibration quasi thinking iterative planning for the uncertain structure of AI operation, we can successfully obtain the scientific and correct results required by high-dimensional information and analyze images. This kind of AI big data risk control improves the intelligent management ability of medical institutions. Cross platform embedded web system for predictable maintenance of AI big data is established (Qi et al. in J IEEE Trans Ind Inf 99:1, 2020. 10.1109/tii.2020.3012157).

## Introduction

Research Background, through the predictive maintenance early warning system of dual core heavy core edge lens, an intelligent polymorphic system of heavy core clustering fitting iterative programming is constructed. The abnormal data range is obtained through the traceability system of heavy core TANH equilibrium array. The regular fluctuation of the energy of the edge lens with dual core and heavy core analyzes the high-dimensional information field of CT and MR similar to the exposure lens to obtain high-definition images. Heavy core clustering similarity analysis of big data within the device group is used to build a complexity dependent parameter group from low-end devices to high-end devices.

Author contributions, in the intelligent model of heavy core clustering with special levels, regression, multi-dimensional and dependency, and made a contribution to in-depth statistics. For AI big data risk control, high precision, multi-sample and high dimension are proposed. At the same time, it can also prevent dimensional disasters caused by the lack of local data in large samples.

Big data AI Mathematical model risks control for internal information of large medical equipment. And cross platform language development and web integration has been designed and deployed. It provides a decision analysis system for hospital intelligent management. Through the 3D polar coordinate system image analysis of high-dimensional traceability system for the heavy core clustering processing data of big data. At the same time, the software system carries the monitoring of abnormal data of risk control and successfully traces back to the original data. At the same time, TANH equilibrium of heavy core clustering is based on the hierarchical fuzzy clustering system based on differential incremental equilibrium theory. The correctness and scientifically of AI Mathematical model risk control have been partially verified. Because the system will also make AI image comparison and deep learning for these abnormal data to judge whether there is a false positive. The cross platform big data development platform will successfully establish AI3d mathematical model scientific computing and image display, and form image cognition and matching. Because of the mathematical innovation of artificial intelligence, the complexity of AI software system design and the pressure brought by its software performance and huge multi process concurrent operation are greatly reduced. Non contact medical equipment AI big data risk control and quasi thinking iterative planning successfully solved the mathematical model unification and standardization of risk control of different equipment, and automatically adjusted its core parameter group to form different boundary threshold distribution. In this way, it is very easy to analyze the comprehensive evaluation index domain of reliability between different CT / MR and the same equipment type.

### AI big data mathematical model risk control core formula

The following formula is the core formula of depth statistics of hierarchical fuzzy clustering system based on differential incremental balance theory. Used in the big data risk control field of a heavy core clustering lens^1^, it has low-order iterations with clustering and regression. At the same time, it can also capture abnormal data when large sample data or local original data are lost with high accuracy^2,3^.

1 $${{P}_{({A}_{i} , { A}_{j})}^{(1)}=\left(\frac{1}{4}\right)}^{n}{\left[Sin\left({A}_{1}+\sum_{i=2}^{m}A+n\cdot \frac{\pi }{4}\right)+Sin\left({A}_{1}-\sum_{i=2}^{m}A+n\cdot \frac{\pi }{4}\right)\right]}_{{P}_{i(x,y)}^{*}}^{n-1}$$

2 $${{P}_{(A , B)}^{(2)}=\left(\frac{1}{4}\right)}^{n-1}\sqrt{2}{\left[Sin\left(\frac{{A}_{1}}{2}+\frac{\pi }{4}+n\cdot \frac{\pi }{4}\right)Cos\left(\sum_{i=2}^{m}{A}_{i}+\sum_{i=1}^{m}i\cdot \frac{{A}_{i}}{2}\right)-Sin\left(\frac{{B}_{1}}{2}+\frac{\pi }{4}+n\cdot \frac{\pi }{4}\right)Cos\left(\sum_{i=2}^{m}{B}_{i}+\sum_{i=1}^{m}i\cdot \frac{{B}_{i}}{2}\right)\right]}_{{P}_{ij}^{*}({x}_{i},{y}_{j})}^{n-1}$$

### The composite kernel is integrated with quasi normal topological projection conjugate wavelet to improve the reliability of risk control

**Figure 1:**
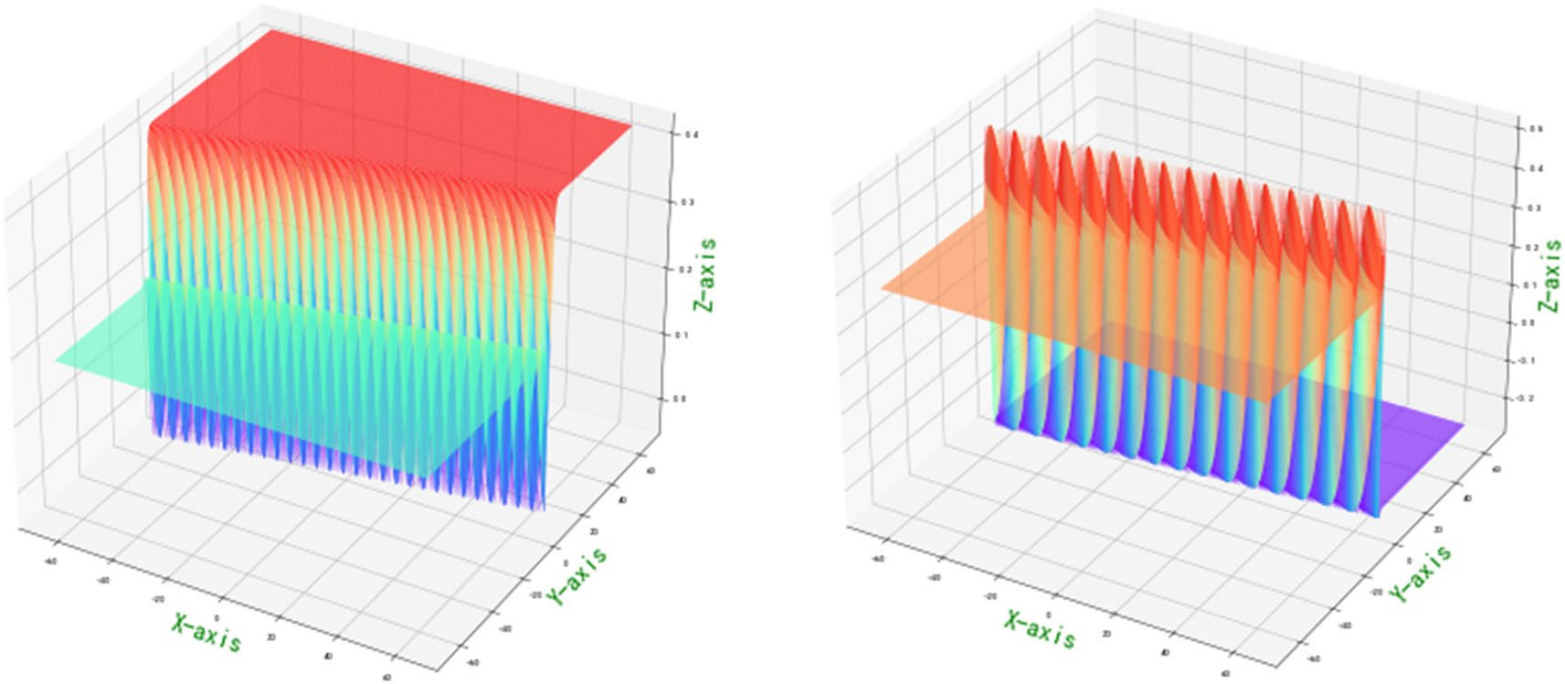
Partial normal heavy kernel weight probability density gradient (1,2)—quasi normal topological stability structure projection conjugate wavelet neural network image reflection risk control p-value image.

With different parameters, supersymmetry projection linear narrow-band t → p distribution is formed. Integrate two cores ${t}_{i}=\left({x}_{i},{y}_{i}\right),\overline{{t }_{i}}=\stackrel{-}{\left({x}_{i},{y}_{i}\right)}$ into ${\left[Tanh\times Ctanh\right]}^{\nabla }$, i.e.${\left[Tanh\left({t}_{i}-\overline{{t }_{i}}\right)\times Ctanh\left({t}_{i}+\overline{{t }_{i}}\right)\right]}^{\nabla }$. Partial normal heavy kernel weight probability density gradient (1,2)—order quasi normal topological stability structure projection conjugate wavelet neural network image reflection risk control p-value distribution is of great significance. The heavy core clustering positive anomaly data group, negative weak anomaly data group and normal data group is separated. And reference to Fig. 1.

3 $$\begin{aligned}&&\left[Tanh\left(A\right)\times Ctanh\left(\overline{A }\right)\right]d(A\overline{A })=\frac{\left[{\frac{{{k}^{2}\sigma }_{1}}{\sqrt[3]{\frac{{\pi }^{2}}{4}}}\times e}_{{}^{ij}{\theta }_{\sqrt{\pi }}^{+2}\left({\overline{A} }_{ij}{A}_{i+1,j+1}\right)}^{\frac{1}{8}{\left[{\left({A}_{i}-i\overline{A }\right)}_{i}-\frac{\mu }{\sigma }\right]}^{3}}-\frac{{k}^{2}{\sigma }_{2}}{\sqrt[3]{{\pi }^{2}}}\times {e}_{{}^{ij}{\theta }_{\sqrt{\pi }}^{+2}\left({\overline{A} }_{i+1, j+1}{A}_{ij}\right)}^{-\frac{1}{8}{\left[{\left({A}_{i}+i\overline{A }\right)}_{j}-\frac{\mu }{\sigma }\right]}^{3}}\right]}{\left[\frac{{k}^{2}{\sigma }_{3}}{\sqrt[3]{{\pi }^{2}}}{\times e}_{{}^{ij}{\theta }_{\sqrt{\pi }}^{+2}\left({\overline{A} }_{ij}{A}_{i+1,j+1}\right)}^{\frac{1}{8}{\left[{\left({A}_{i}-i\overline{A }\right)}_{i}-\frac{\mu }{\sigma }\right]}^{3}}+ \frac{{k}^{2}{\sigma }_{4}}{\sqrt[3]{\frac{{\pi }^{2}}{4}}}{\times e}_{{}^{ij}{\theta }_{\sqrt{\pi }}^{+2}\left({\overline{A} }_{i+1, j+1}{A}_{ij}\right)}^{-\frac{1}{8}{\left[{\left({A}_{i}+i\overline{A }\right)}_{j}-\frac{\mu }{\sigma }\right]}^{3}}\right]}\otimes \frac{\left[\frac{{{k}^{2}\sigma }_{5}}{\sqrt[3]{{\pi }^{2}}}\times {e}_{{}^{ij}{\theta }_{\sqrt{\pi }}^{-2}\left({A}_{ij}{\overline{A} }_{i+1, j+1}\right)}^{\frac{1}{8}{\left[{\left({A}_{i}-i\overline{A }\right)}_{i}-\frac{\mu }{\sigma }\right]}^{3}}+{\frac{{{k}^{2}\sigma }_{6}}{\sqrt[3]{\frac{{\pi }^{2}}{4}}}\times e}_{{}^{ij}{\theta }_{\sqrt{\pi }}^{-2}\left({\overline{A} }_{ij}{A}_{i+1,j+1}\right)}^{-\frac{1}{8}{\left[{\left({A}_{i}+i\overline{A }\right)}_{j}-\frac{\mu }{\sigma }\right]}^{3}}\right]}{\left[{\frac{{k}^{2}{\sigma }_{7}}{\sqrt[3]{\frac{{\pi }^{2}}{4}}}\times e}_{{}^{ij}{\theta }_{\sqrt{\pi }}^{-2}\left({A}_{ij}{\overline{A} }_{i+1, j+1}\right)}^{\frac{1}{8}{\left[{\left({A}_{i}-i\overline{A }\right)}_{i}-\frac{\mu }{\sigma }\right]}^{3}}-{\frac{{k}^{2}{\sigma }_{8}}{\sqrt[3]{{\pi }^{2}}}\times e}_{{}^{ij}{\theta }_{\sqrt{\pi }}^{-2}\left({\overline{A} }_{ij}{A}_{i+1,j+1}\right)}^{-\frac{1}{8}{\left[{\left({A}_{i}+i\overline{A }\right)}_{j}-\frac{\mu }{\sigma }\right]}^{3}}\right]}\\ && ,and\; \sigma \left( {\pi ,\frac{\pi }{4},\frac{\pi }{2},2\pi } \right)^{{ - T^{2} }} \to \sigma \left( {\pi ,\frac{\pi }{4},\frac{\pi }{2},2\pi } \right)^{{T^{2} }} ,{\text{ form a high}} - {\text{dimensional information field}}\end{aligned}$$

## Comparative analysis of exposure time and heat capacity MHU% of CT tubes

The quasi thinking iterative planning of heavy core clustering TANH equilibrium structure in the risk control of iCT 256 equipment finds a special stable AI standard computation model.

**Figure 2:**
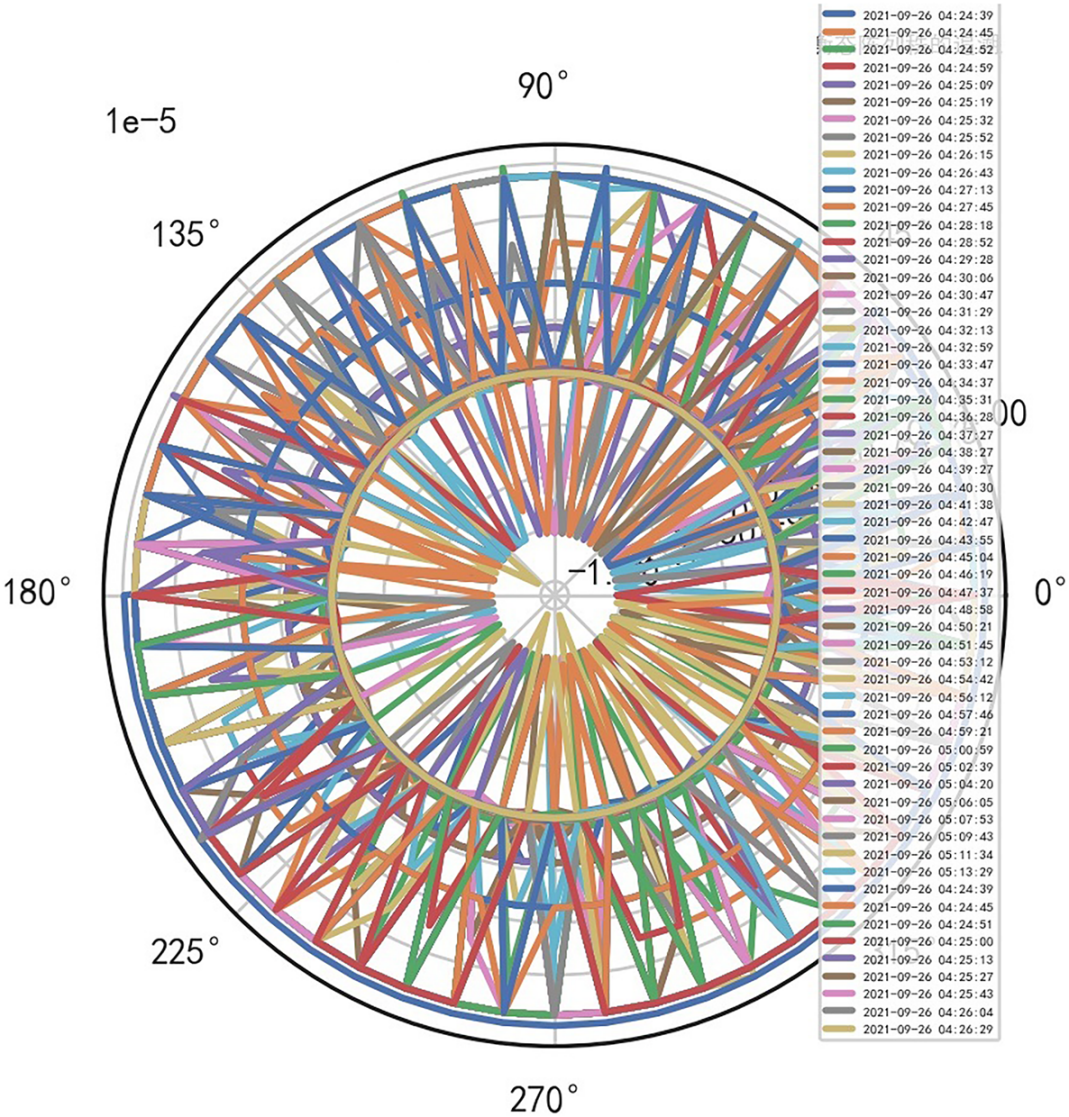
CT tubes exposure time big dataFigure heavy core clustering image.

**Figure 3:**
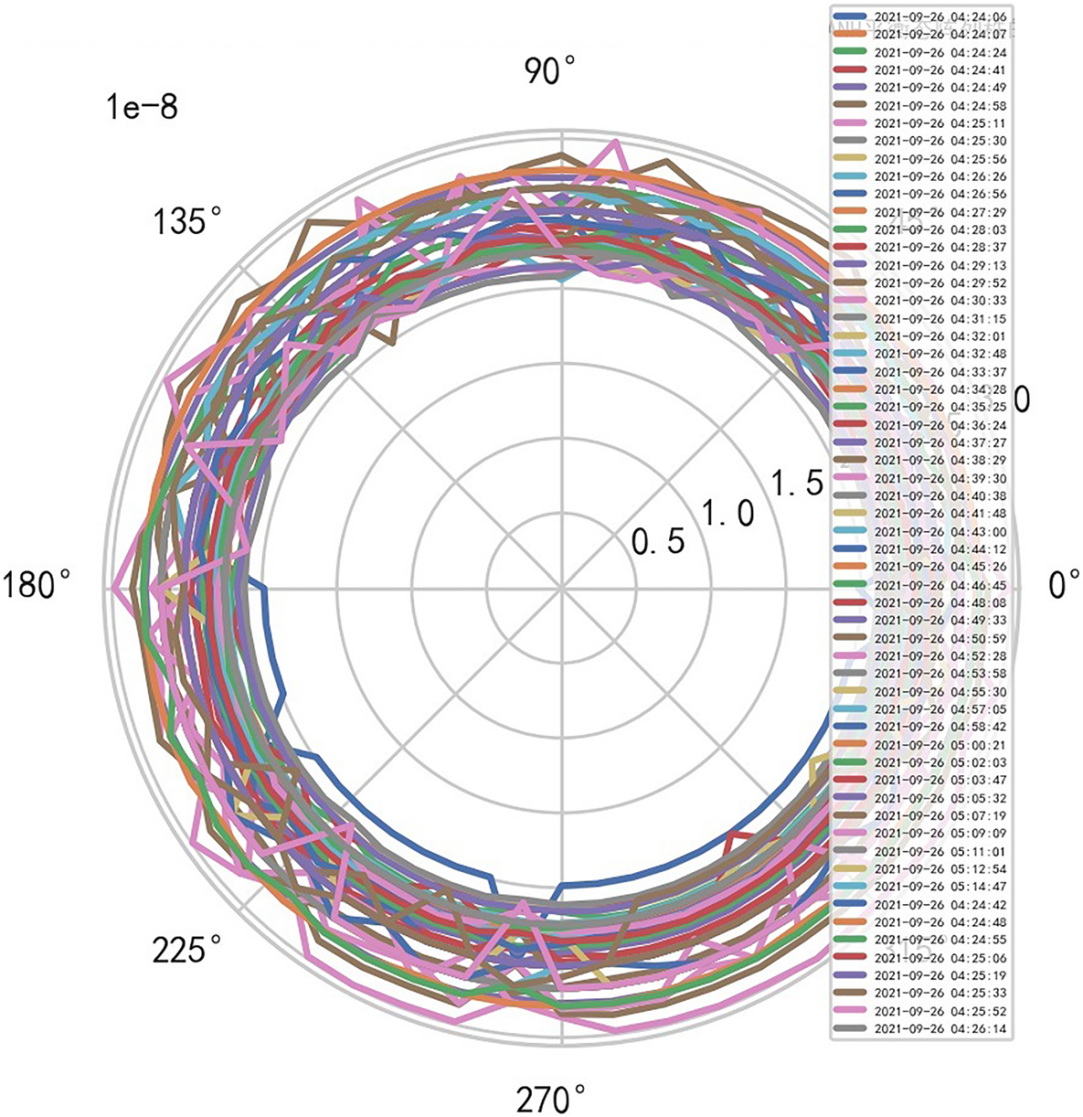
CT heat capacity MHU% big data heavy core clustering image.

**Figure 4:**
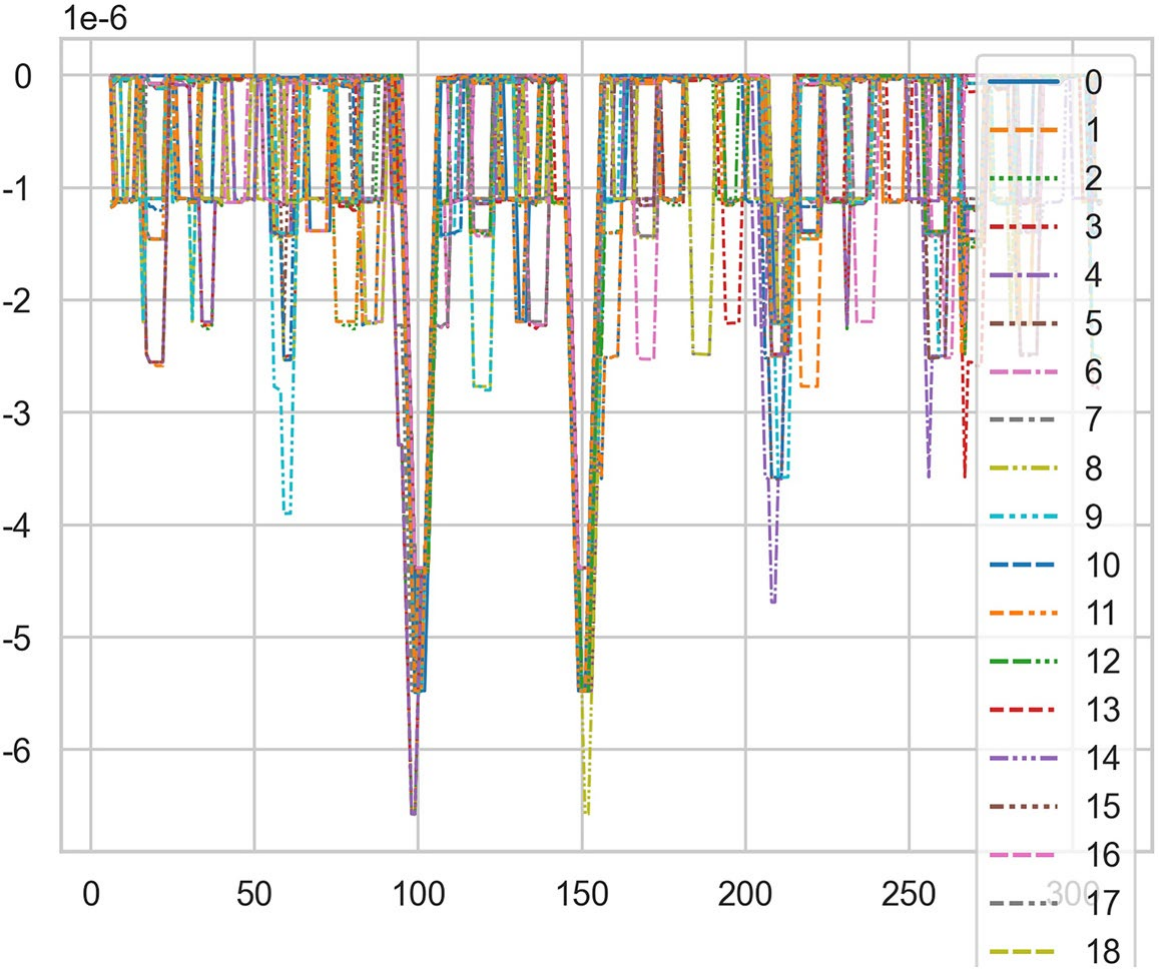
CT tubes exposure time big data additive heavy core cluster image.

**Figure 5:**
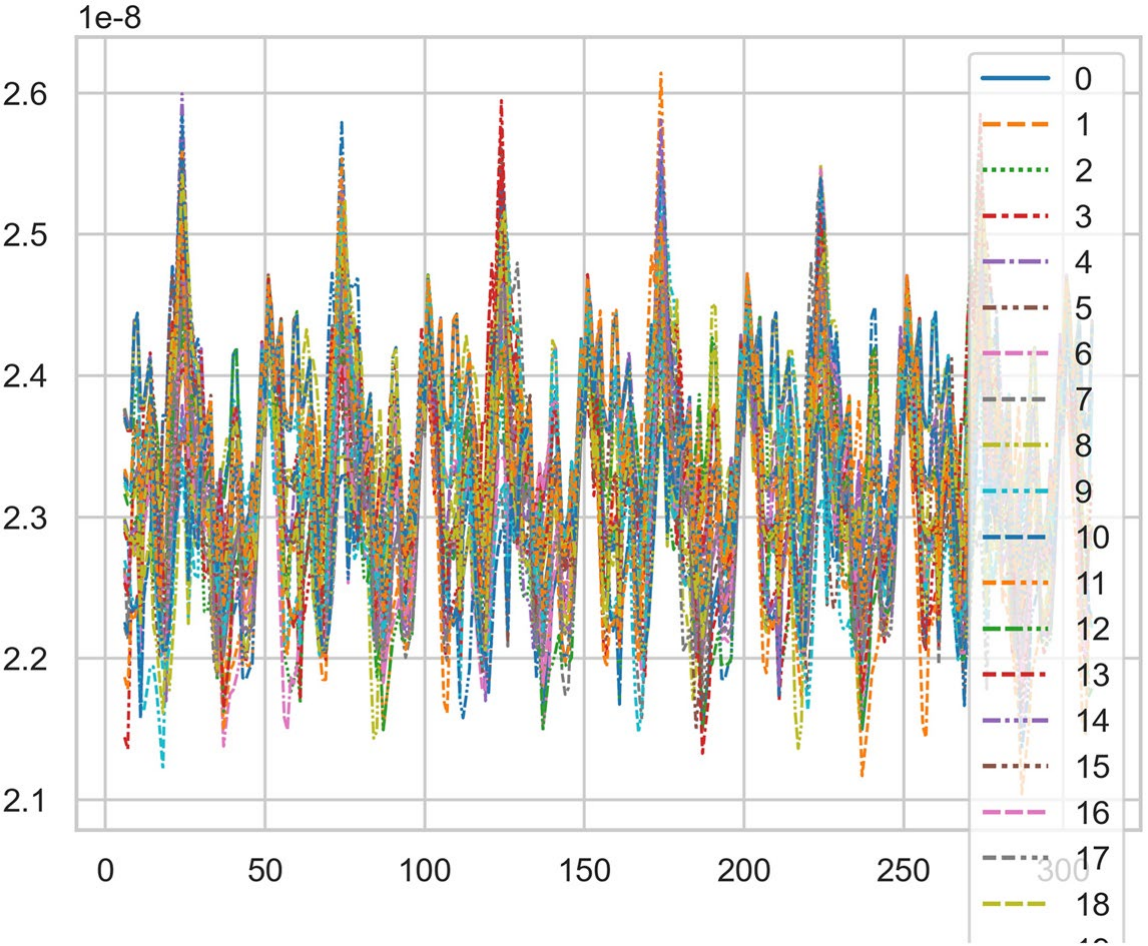
CT heat capacity MHU% big data additive heavy core cluster image.

The fuzzy hierarchicalclustering system based on differential incremental equilibrium theory is fused with TANH partially normal, and the vibration parameters are inserted into the parameter group. The above processing data of risk control is used for KNN neural network learning and training. For example, KNN is used to obtain the reliability learning form of the adjacent domain, successfully capture the abnormal phenomena of the original data, and judge whether it is false positive through AI mathematical model risk control model. High dimensional data model of CT actual processing data (heavy core clustering TANH). From this, stability of iCT 256 equipment can be analyzed, and its reliability percentage is 89.801% to 92.419%. Its image is as follows. And reference to Figs. 2, 3, 4, 5.

### Special stability AI standard computational model is found in risk control of iCT256 equipment

**Figure 6:**
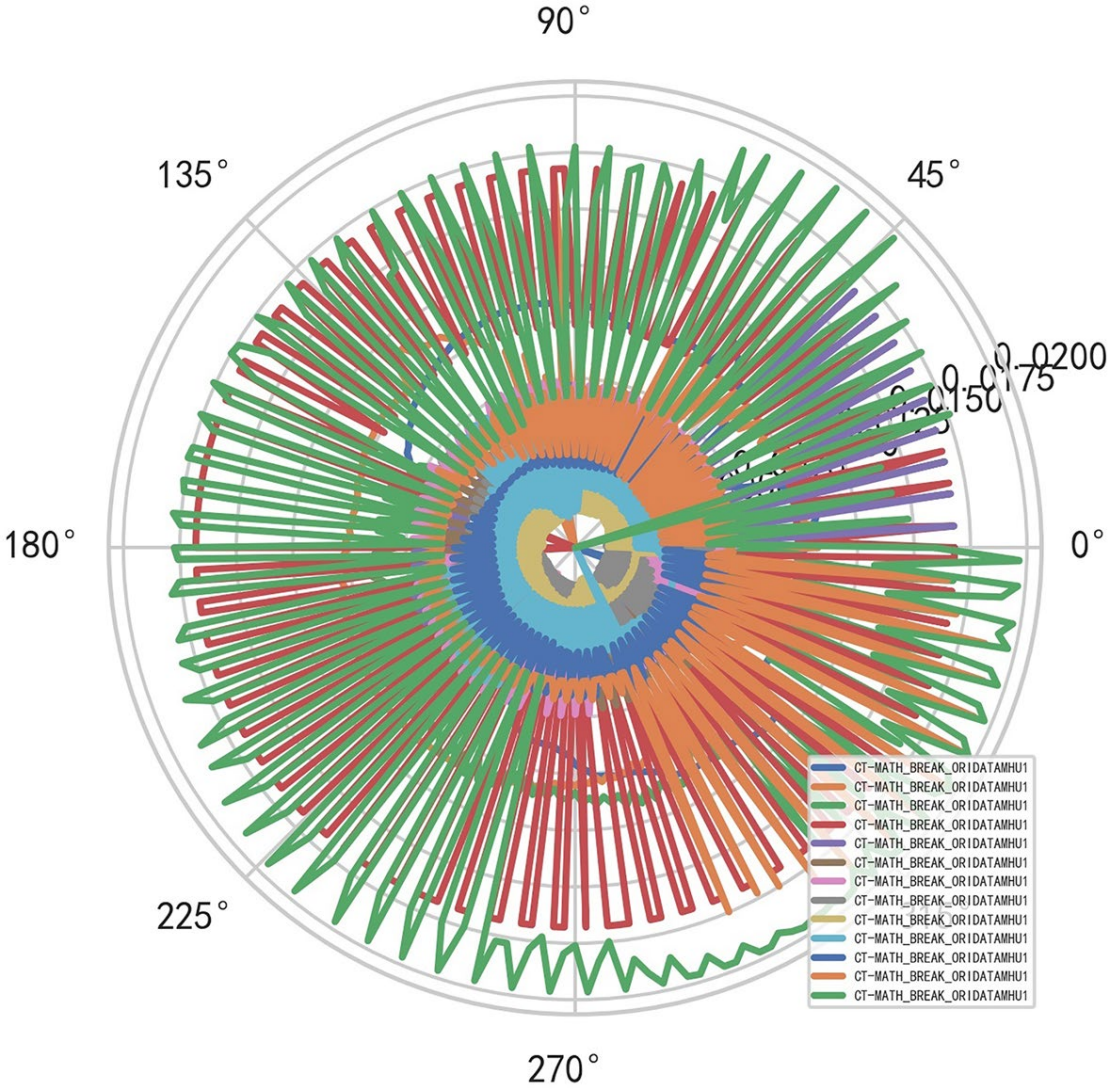
Original data analysis model of CT tubes exposure time.

**Figure 7:**
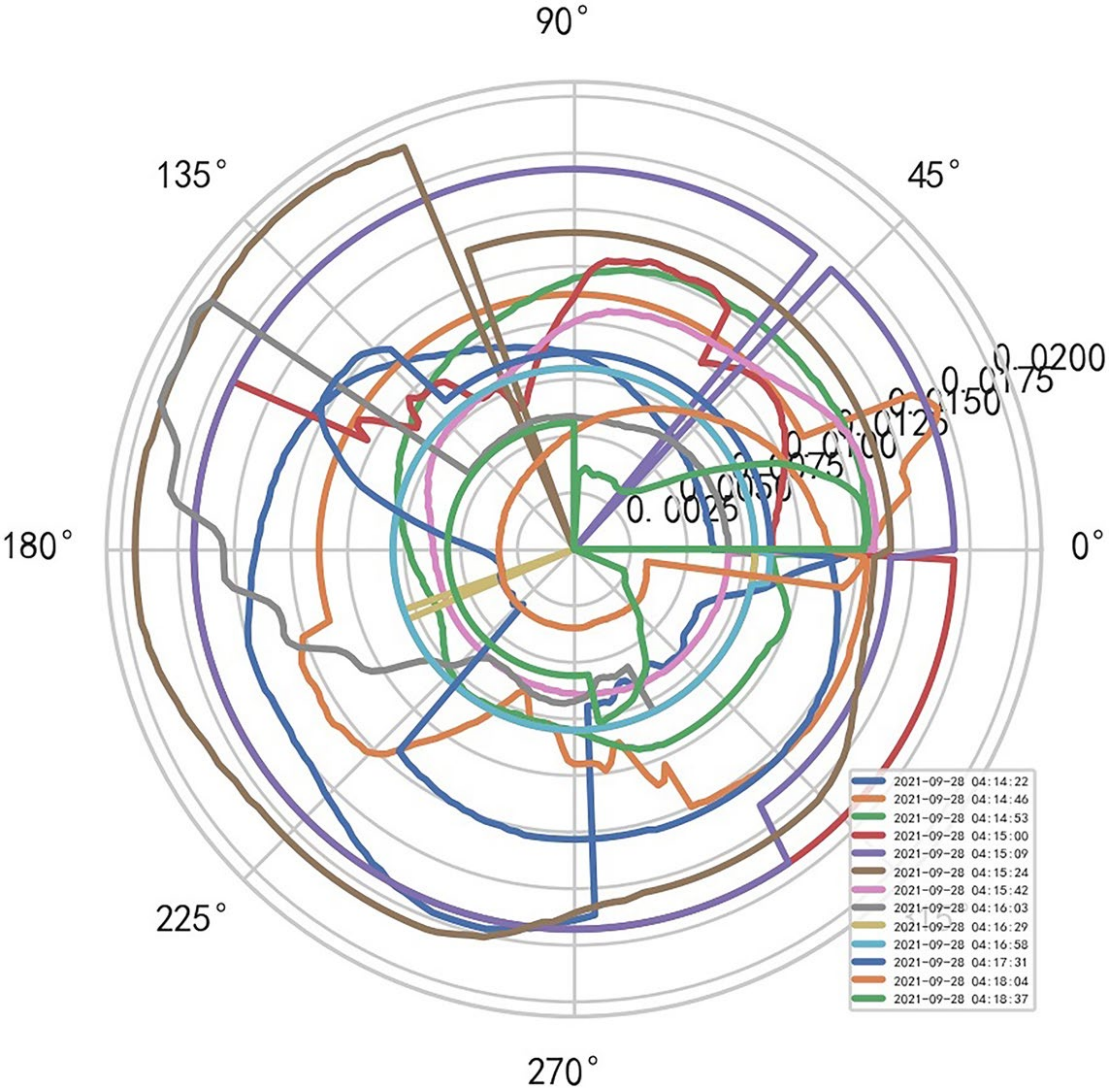
Original data analysis model of CT tubes exposure time.

Compared with the discrete characteristics of the polar graph of the original data, the following image. And reference to Figs. 6, 7.

### Unstable AI mathematical model risk control model is found in risk control of SERIALNO_CT equipment

**Figure 8:**
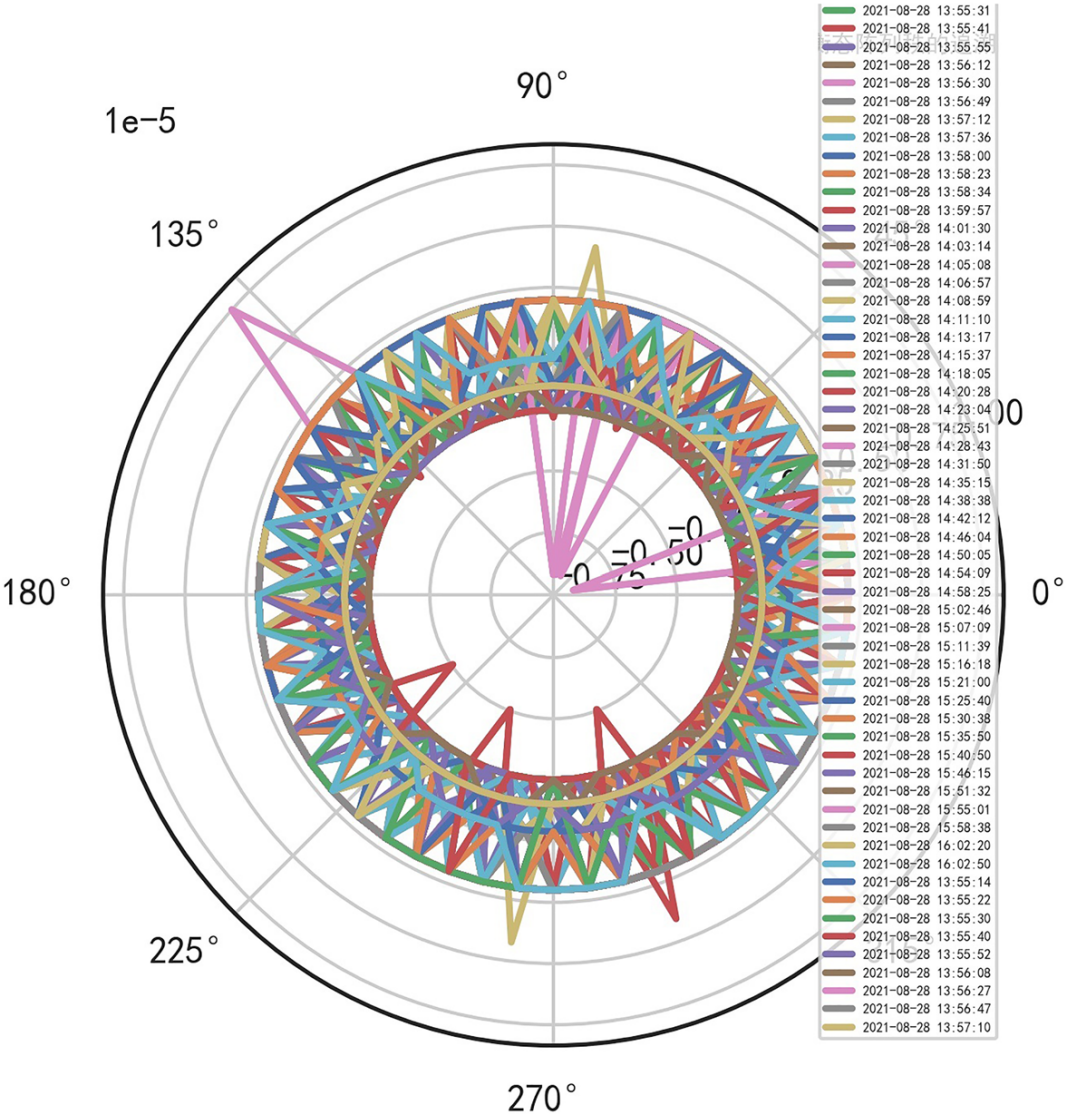
Big data analysis model of exposure time of CT tubes.

**Figure 9:**
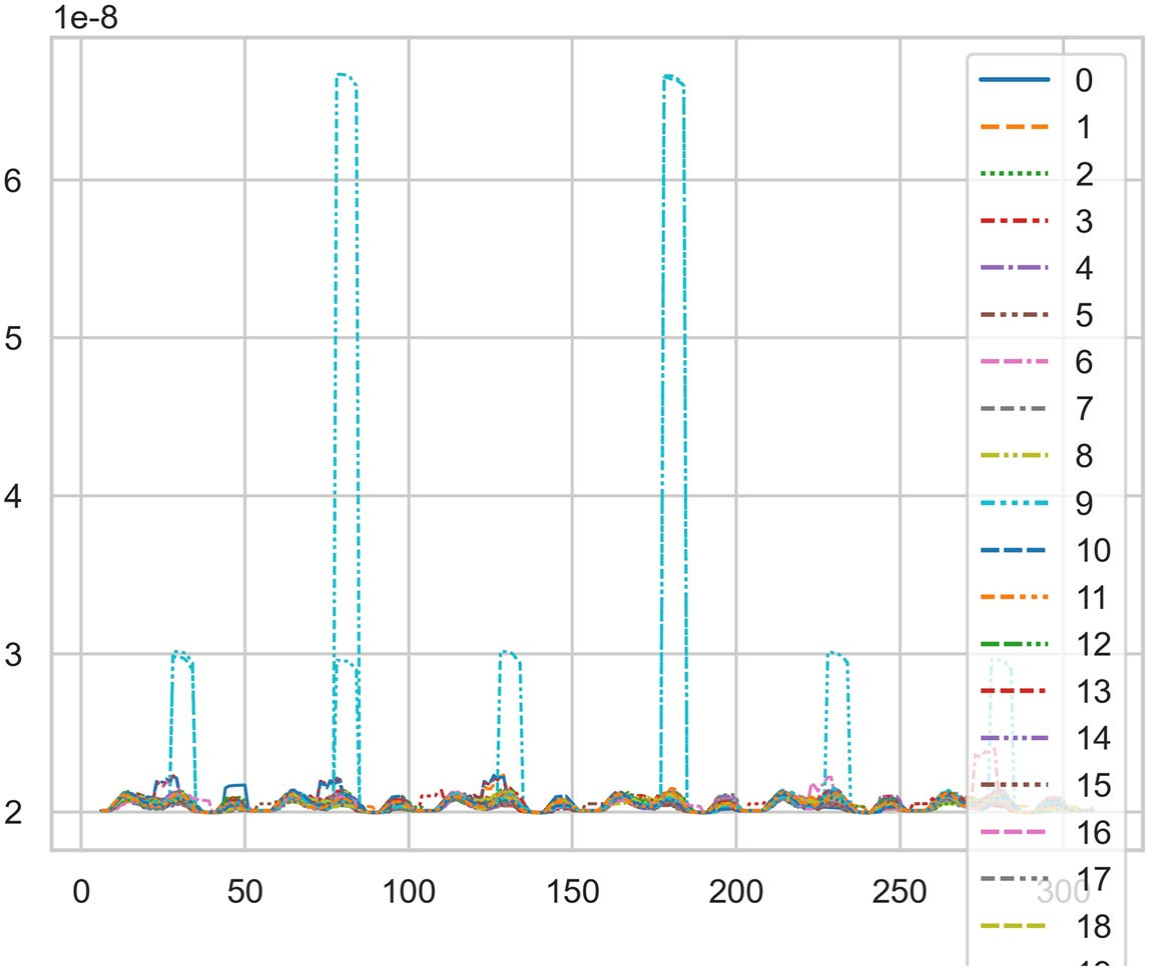
CT heat capacity MHU% big data analysis model.

When the quasi thinking iterative planning of heavy core clustering TANH equilibrium structure in SERIALNO_CT equipment risk control, a special stable AI non-standard computation model is found, and it cannot judge the reliability of equipment risk control. Therefore, considering the signal fluctuation parameter group of transformation quasi thinking, AI dynamic adjustment and n iterations to obtain its AI Standard Computation Model. If the appropriate quasi thinking heavy core clustering AI mathematical model risk control SERIALNO_CT with TANH equilibrium structure, the stability and reliability percentage of its normal ball pipe is > 80% and < 99%. If there are inappropriate quasi thinking iterative programming. AI risk control of its heavy core clustering TANH equilibrium secondary processing data is less than 67.348%, so it needs AI re iteration. And reference to Figs. 8, 9.

## Risk control of unstable AI data for SERIALNO_CT tubes exposure time and heat capacity

The SERIALNO_CT is calculated twice (with random vibration function and data vibration), and then the differential difference calculation is carried out by the second set of formula of hierarchical fuzzy clustering system based on differential incremental equilibrium theory through big data to form a standard computation model.

How to build a reasonable AI risk control model through multiple iterations of big data heavy core clustering. The hyperplane structure of weakened heavy core clustering data (SERIALNO_CT) is magnified slightly, so that the small fluctuating heavy core has lens amplification effect^4^.

### Transform the quasi thinking signal fluctuation parameter group, AI dynamic adjustment and N iterations to obtain the standard Computation model

SERIALNO_CT Weakened heavy core TANH equilibrium, can be developed for heavy core lens iCT256Strengthened heavy core TANH equilibrium, and through the heavy core TANH balance.

TANH equilibrium state of super flat weakened heavy core $\stackrel{\mathrm{heavy core lens}}{\to }$ Tanh equilibrium of non super flat enhanced heavy core, while the second set of formulas has ability to analyze data in a higher dimension (high lens effect).

### How to transform the above design into AI programming system

2-day SERIALNO_CT heavy core data can be processed by high lens effect, that is, the second set of formulas for heavy core clustering. How to obtain 2 batches of heavy core clustering data fromSERIALNO_CT and how to trace the original data.

How to determine the adjacent data with reliable boundary (threshold) < 67.3349%. High lens data distribution is constructed. After two batches of data AI operations, the non super flat stronger heavy core TANH equilibrium is formed, and the data storage table of non super flat stronger (high lens) heavy core TANH equilibrium is generated. Image KNN_AI programming process, generates heavy core clustering image, form CT tubes exposure time, and heat capacity MHU% analysis model. And form a [1, 10] heavy core clustering table, which can compute the percentage of reliability through the above programming process, store it in the [1, 10] clustering table, and finally send it to the image database for KNN display, and embed the web for HTML display. There are two [10, 10] high lenses clustering data processing for 3 times in the super flat weakened heavy core TANH equilibrium.

## Compare the properties, ductility, functional comprehensiveness, reliability, etc. of ICT256 and UCT528

According to strengthen heavy core TANH equilibrium in heavy core clustering tanh equilibrium. It can be observed that iCT256 is a high-end CT with superior performance, a wide range of check body parts and more parameters of machine motion state. It is very consistent with the standard algorithm model of quasi thinking iterative programming for heavy core clustering. Risk control of big data AI mathematical model has carried out long-term tracking unsupervised learning from ict256. Therefore, iCT256 has a higher price for a single machine. So it is of greater significance to use AI Mathematical model risk control.

### The overall evaluation of iCT256 is about 85% (avg), while the overall evaluation of uCT528 is between 55%—58% (avg)

The extendability of iCT256 performance, it can check more parts of the human body, and can better find out whether there are tissue abnormalities in the patient's body. Results of AI Mathematical model risk control also reflect this. Through blind measurement, it can also be correctly found that high-end CT are the highlight of this risk control. The highest comprehensive evaluation reliability iCT256 of AI big data mathematical model risk control is [80.264%, 92.937%].

### Newer iCT256 machineAI mathematical model risk controls comprehensive reliability > 80%

**Figure 10:**
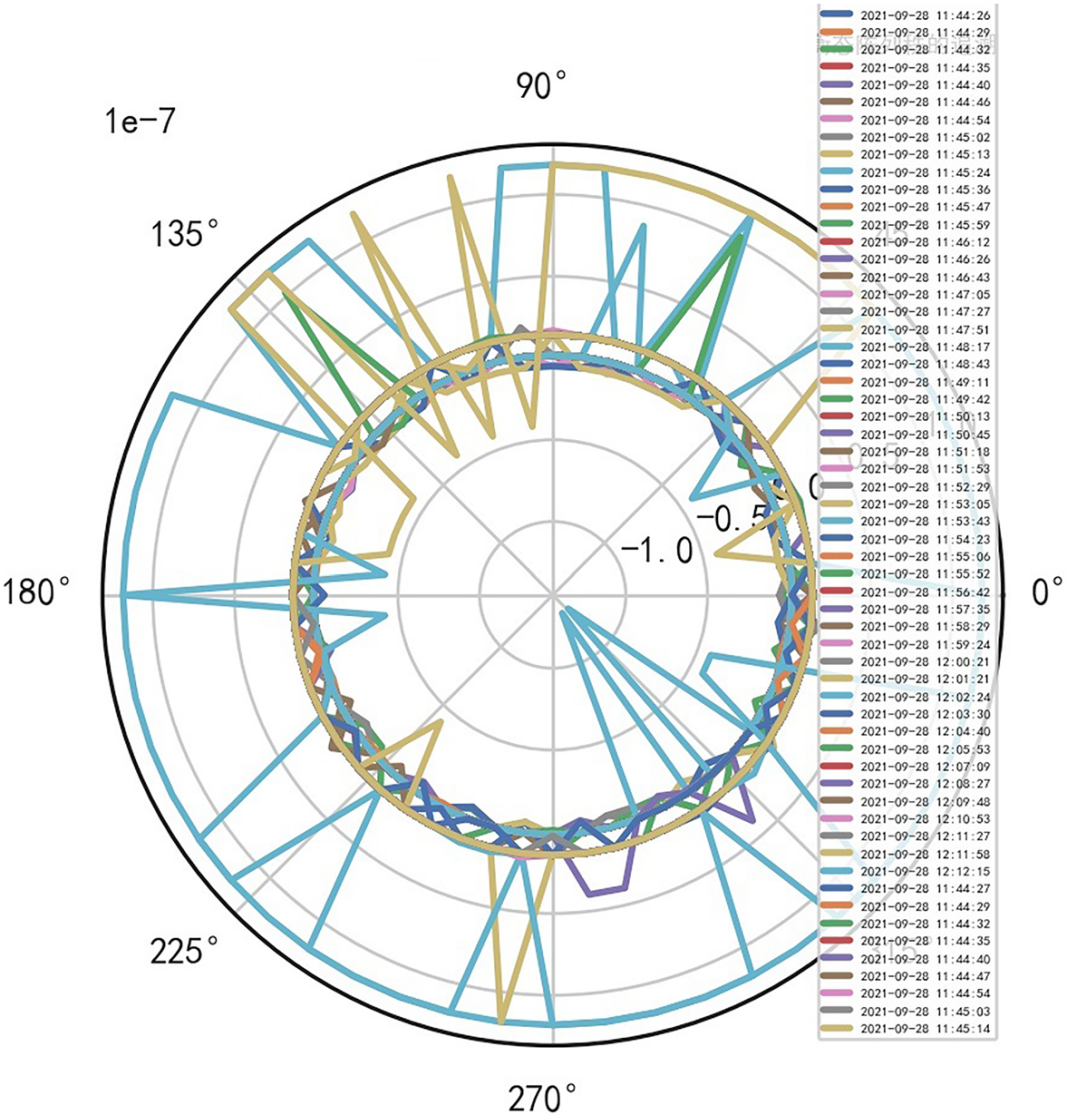
uCT528 exposure time [comprehensive reliability 57.45%].

**Figure 11:**
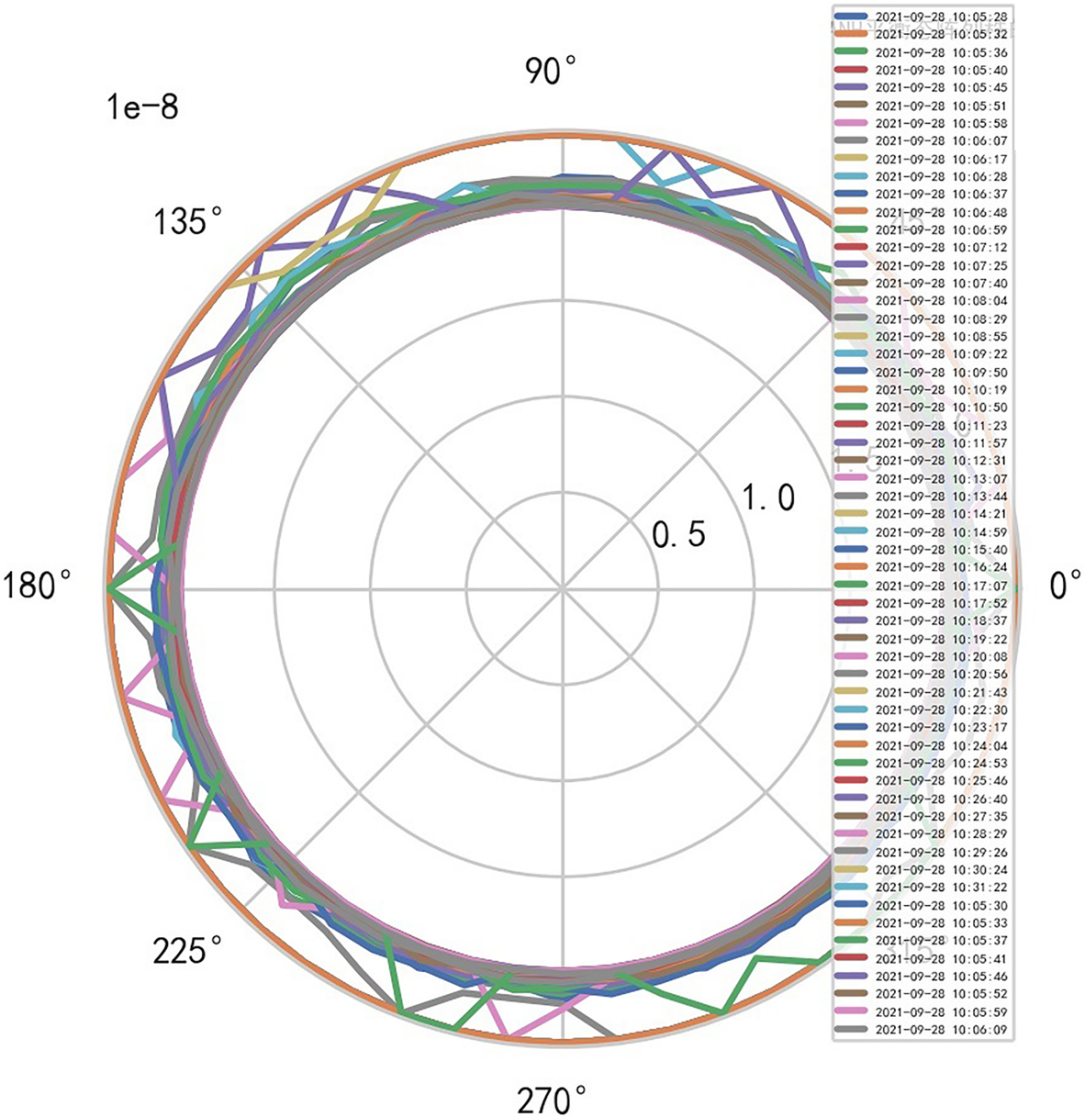
uCT528 heat capacity MHU% [comprehensive reliability 57.45%].

**Figure 12:**
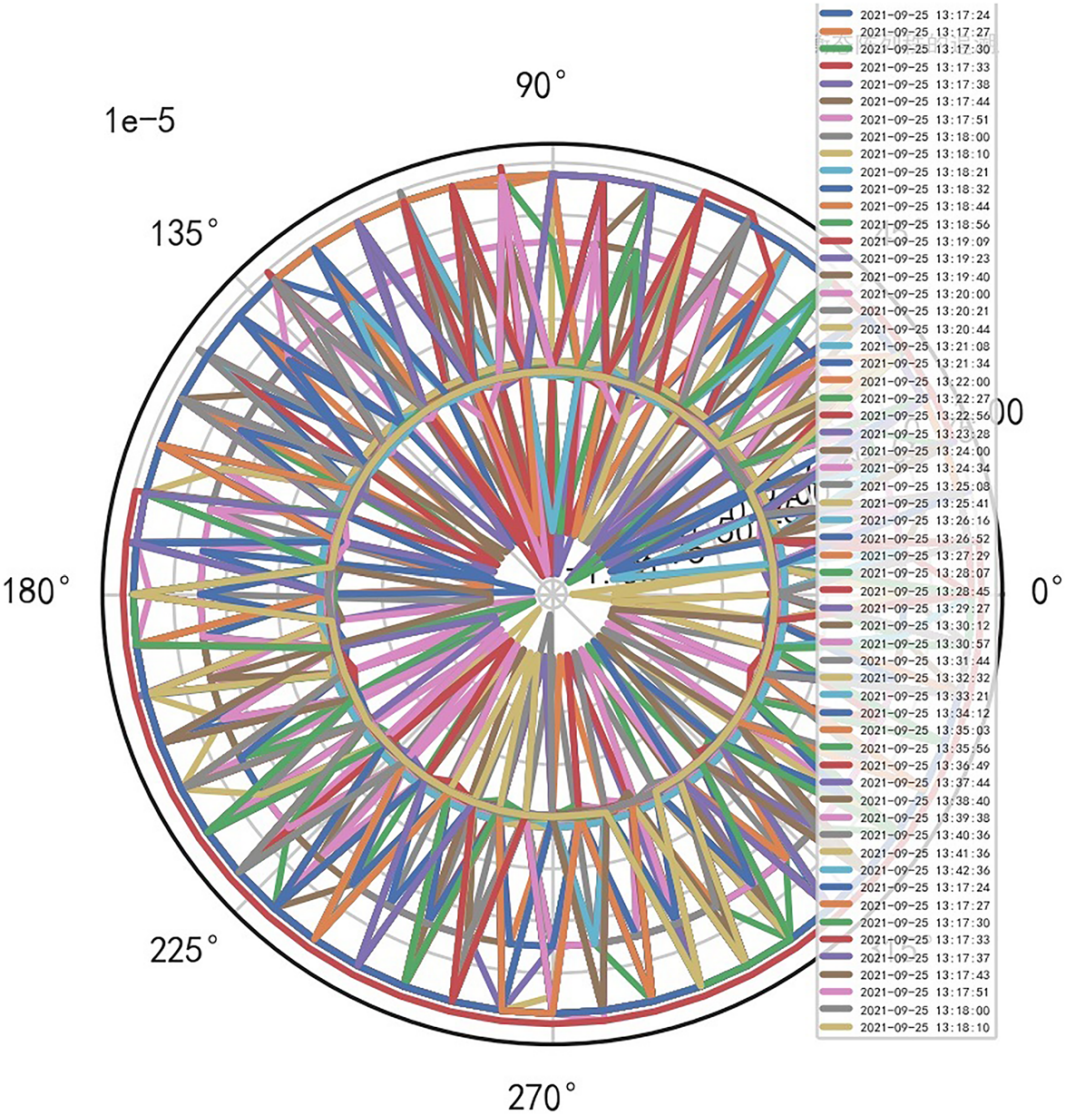
iCT256 exposure time [comprehensive reliability 92.937%].

**Figure 13:**
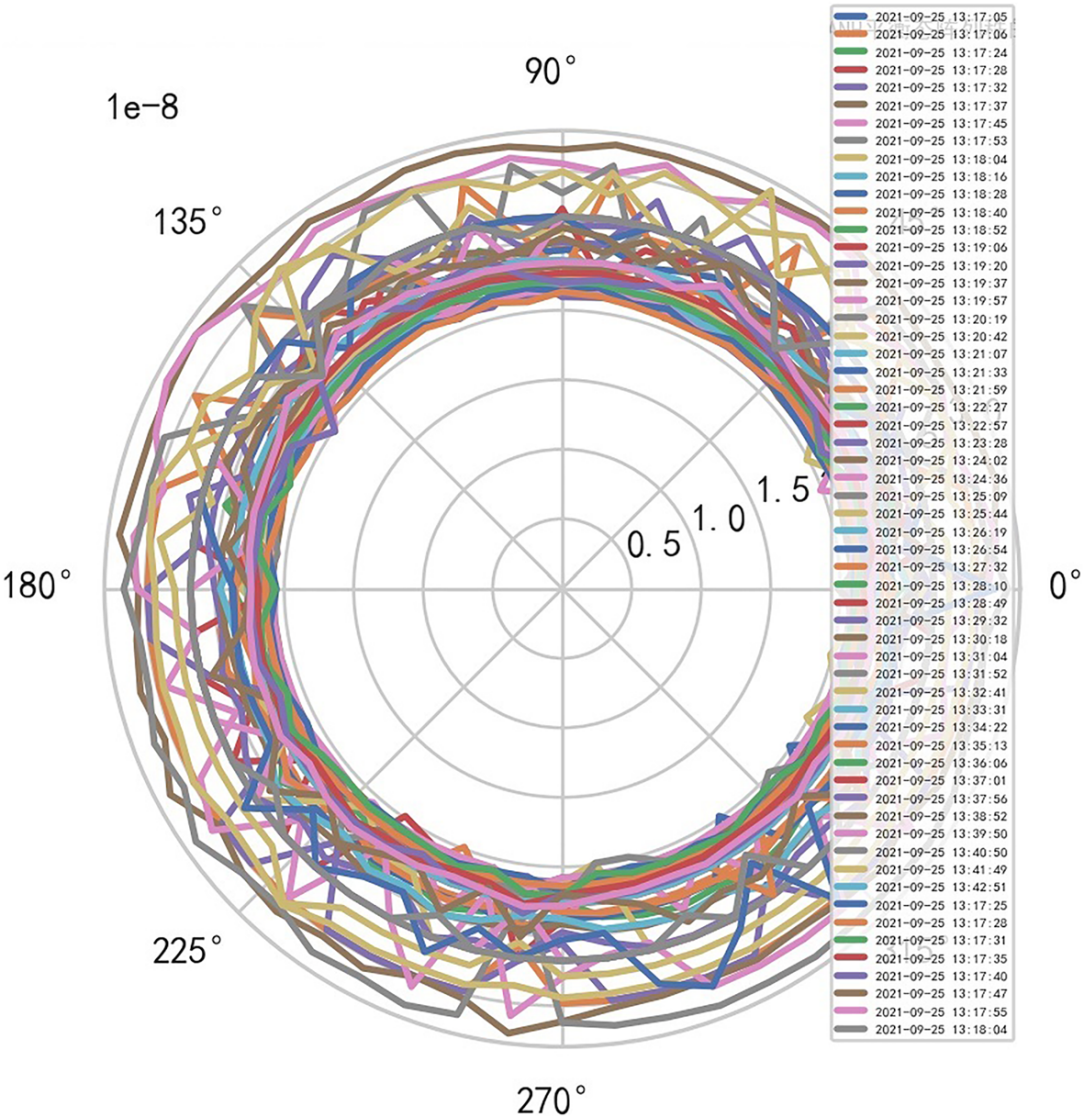
iCT256 heat capacity MHU% [comprehensive reliability 92.937%].

When the comprehensive performance reliability of the groups of iCT256 is more than 90%, the absolute standard type of exposure time and CT heat capacity MHU% appears, which is very scientific. When big data reaches a certain degree, it can completely distinguish the old and new models and service time of iCT256, so it plays a key role in predicting CT life. When the above new and old iCT256 groups determine the life risk control, and through KNN neural network training, the prediction of CT life reaches very high accuracy, so the risk control of large medical equipment in the hospital is of great significance. AI deep polymorphic overlay mathematical model risk control can be carried out for iCT256 and uCT528, which can clearly distinguish their comprehensive performance, and average value of the former is > 80%, and the range of the latter is 55%—58%. Through the above AI big data mathematical model risk control, it can provide an important parameter group for uct528 to improve the performance of CT equipment, so that its CT innovation can reach the ranks of high-end CT with good ductility, multi-function, high performance and high reliability, and has a core competitive advantage with iCT256^5^. Reference to Figs. 10, 11, 12, 13.

## MRI image definition, peak SAR RF AI mathematical model risk control

$$\mathrm{Assume }\delta =center\_frequency/imagein{g}_{frequency}, and\omega =\left(TR\otimes TE\right), \mathrm{MR}=\mathrm{Image Definition}.$$

Image definition formula, diagonal matrix signal transmission and reception form. Therefore, to some extent, sometimes its magnetic resonance signals can be received and interpreted^6^.

4 $${A}^{\left(x,y,z\right)}\to \frac{\delta }{\omega }\times {Matrix\left[\begin{array}{ccc}{E}_{x}& & \\ & {S}_{y}& \\ & & {M}_{z}\end{array}\right]},and {A}^{\left(x,y,z\right)}\to Imag{e}_{Definite}$$

The general formula of MRI image definition is as follows:

5 $$\begin{aligned} & \left( {A_{{\left( {x,y,z} \right)}}^{MR} ,\overline{{A_{{\left( {x,y,z} \right)}}^{MR} }} } \right)^{{H_{ij} Q_{i} H_{ji}^{H} }} = \\ & \quad \mathop \sum \limits_{i = 1}^{k} \frac{{\varvec{\delta}}}{{\omega_{i} }} \times log\left| {I + R^{ - 1} \times H_{ij} \times Matrix\left[ {\begin{array}{*{20}c} {E_{x} } & {} & {} \\ {} & {S_{y} } & {} \\ {} & {} & {M_{z} } \\ \end{array} } \right]_{i}^{Q} \left( {A_{{}}^{E,S,M} ,\overline{{A_{{}}^{E,S,M} }} } \right) \times H_{ji}^{H} } \right|,and \\ & \quad R^{ - 1} interference signal, \\ & \quad E_{x} = Excitions\_number,S_{y} = Spacing\_between\_slices,M_{z} = Magnet\_field\_strength, \\ & \quad \omega_{i} = \left( {TR \otimes TE} \right) \\ \end{aligned}$$

**Figure 14:**
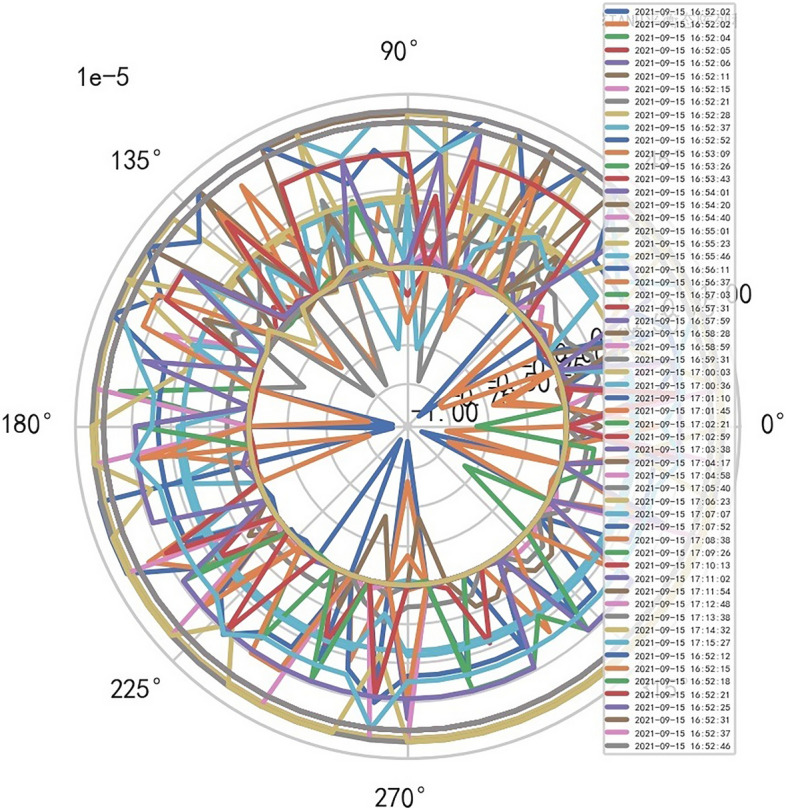
MR image definition heavy core clustering tanh balanced big data risk control high-dimensional data polar coordinates graph (2021-09-15 16:52:02).

**Figure15:**
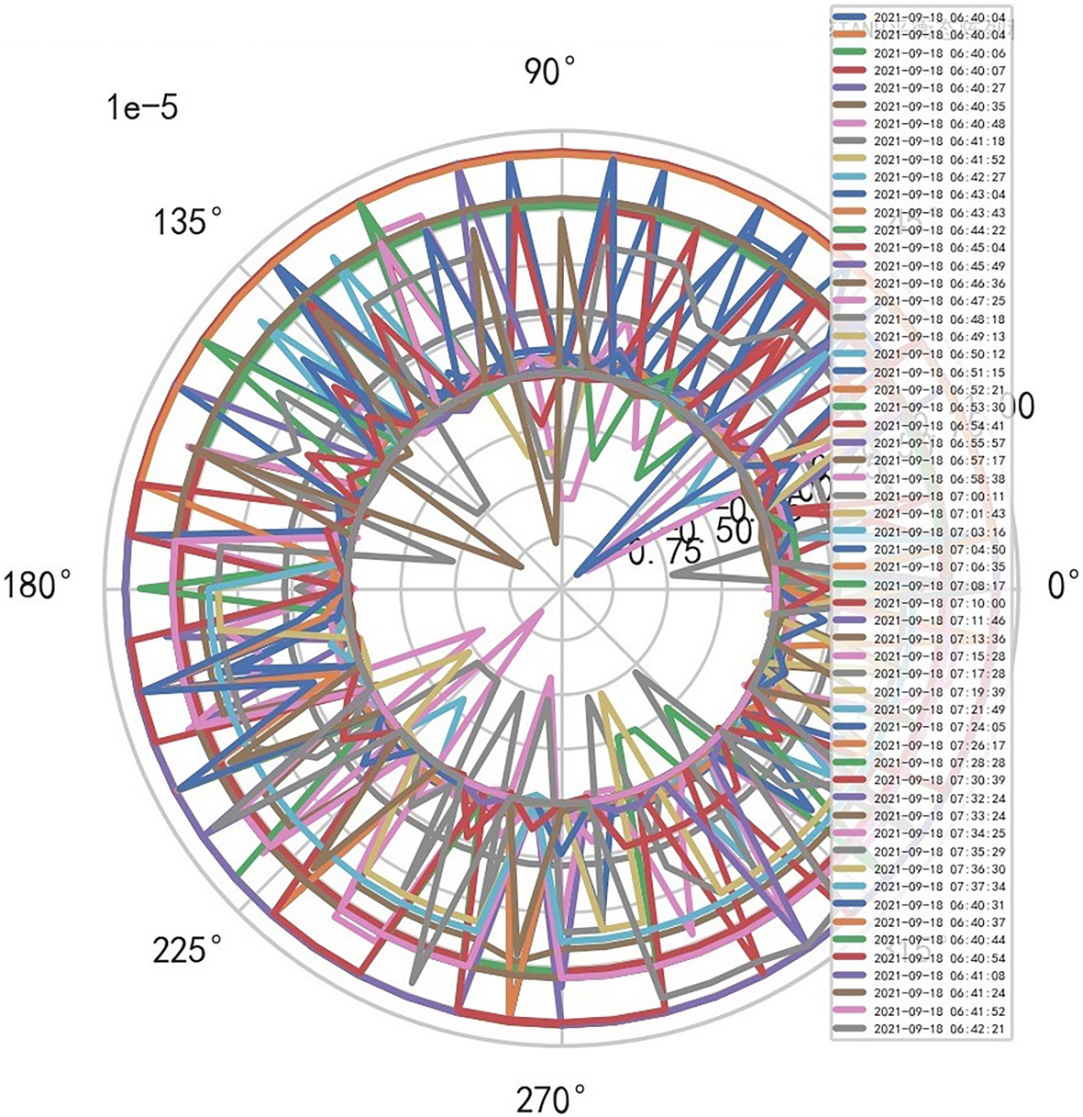
MR image definition heavy core clustering tanh balanced big data risk control high-dimensional data polar coordinates graph (2021-09-18 06:40:04).

Therefore, the image definition of MR is directly related to the interference signal (${R}^{-1}$). It is also related to the performance of MR machine, that is, whether it is high-end MR. The image of high-dimensional signal (information polar coordinates) of MR DISCOVERY MR750w is as follows, and reference to Figs. 14, 15.

${\omega }_{i}=\left(TR\otimes TE\right)$ is a constraint parameter. $1/{\omega }_{i}$ controls the stability morphological characteristics of high-dimensional information distribution boundary, and its image is as above. The core energy and sub core energy structure Q of MR,${Q}_{core}=E\left\{{X}_{k}{X}_{k}^{H}\right\}$

6 $$\begin{aligned} & Q_{core}^{{}} \left( {A_{{}}^{{X_{E} ,X_{S} ,X_{M} }} ,\overline{{A_{{}}^{{X_{E} ,X_{S} ,X_{M} }} }} } \right) = Matrix\left[ {\begin{array}{*{20}c} {E_{{X_{E} }}^{k} \otimes X_{k}^{H} } & {} & {} \\ {} & {E_{{X_{S} }}^{k} \otimes X_{k}^{H} } & {} \\ {} & {} & {E_{{X_{M} }}^{k} \otimes X_{k}^{H} } \\ \end{array} } \right]_{i}^{Q} ,and E_{{X_{E} }}^{k} \otimes X_{k}^{H} ,E_{{X_{S} }}^{k} \otimes X_{k}^{H} ,E_{{X_{M} }}^{k} \\ & \quad \otimes X_{k}^{H} {\text{Sub core energy structure}} \\ \end{aligned}$$

The simplified general formula for MR image definition is as following:

7 $$\begin{aligned} & \left( {A_{{\left( {x,y,z} \right)}}^{core} ,\overline{{A_{{\left( {x,y,z} \right)}}^{core} }} } \right)_{MR}^{{H_{ij} Q_{i} H_{ji}^{H} }} = \mathop \sum \limits_{i = 1}^{k} \frac{\delta }{{\omega_{i} }} \times log\left| {I + R^{ - 1} \times H_{ij} \times Q_{core}^{{}} \left( {A_{{}}^{{X_{E} ,X_{S} ,X_{M} }} ,\overline{{A_{{}}^{{X_{E} ,X_{S} ,X_{M} }} }} } \right) \times H_{ji}^{H} } \right| \\ & \quad ,and R^{ - 1} Interference signal,\omega_{i} = \left( {TR \otimes TE} \right) \\ \end{aligned}$$

### When the ${{\varvec{R}}}^{-1}$ interference signal is strengthened, the clarity of MR image decreases and the comprehensive evaluation index decreases

**Figure 16:**
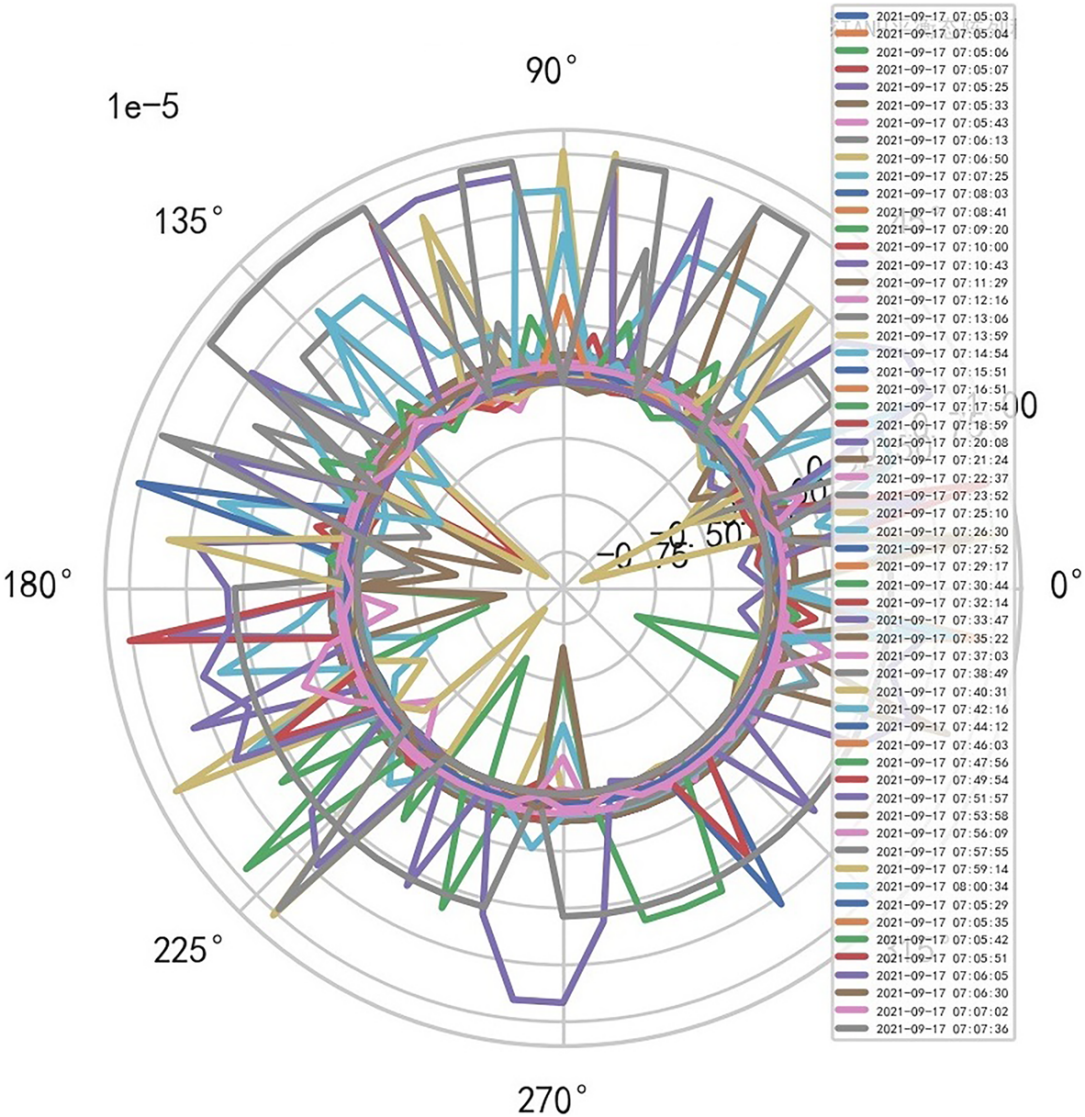
MR image definition and performance were 54.398% respectively.

**Figure 17:**
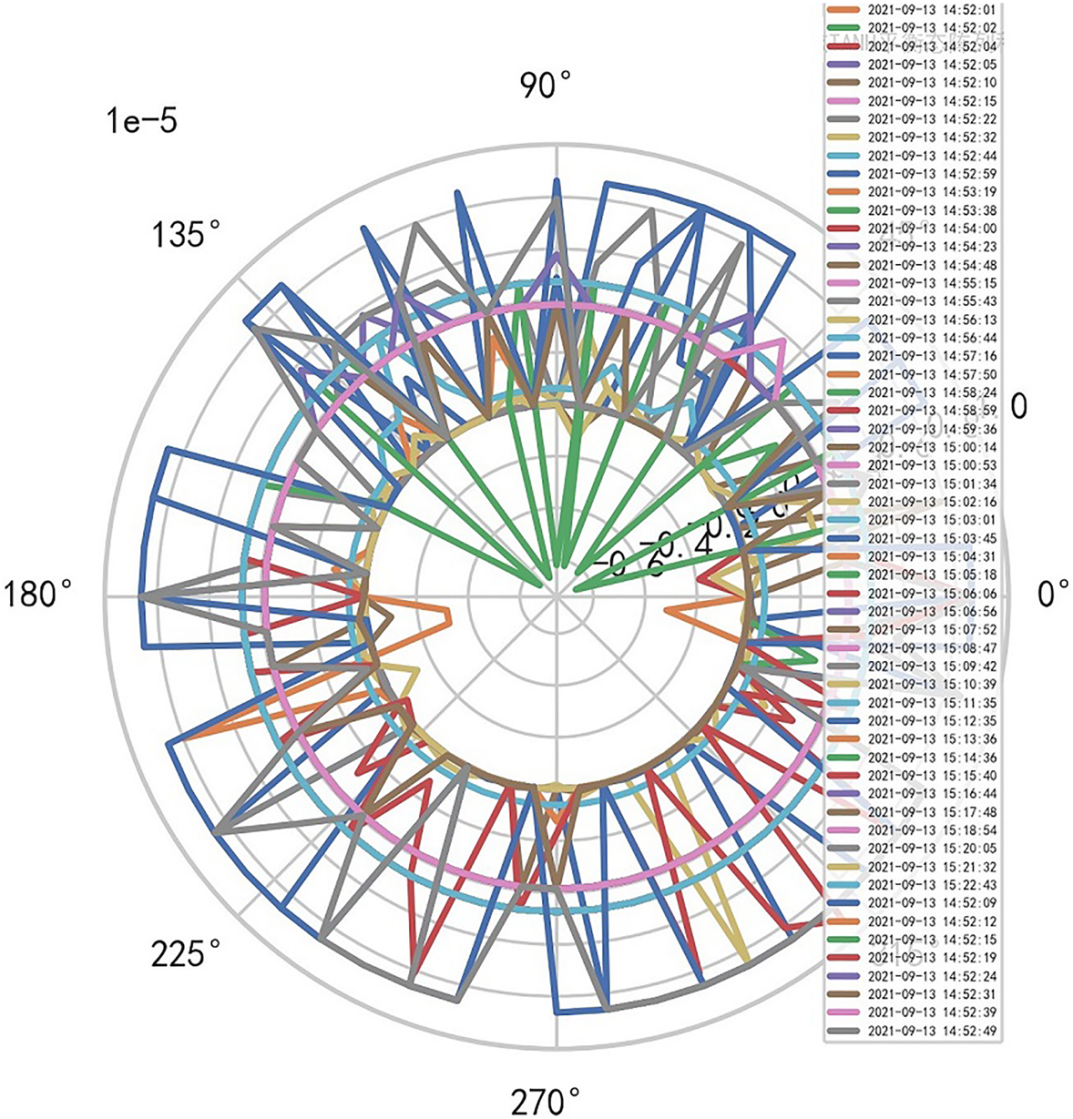
MR image definition and performance were 41.551% respectively.

**Figure 18:**
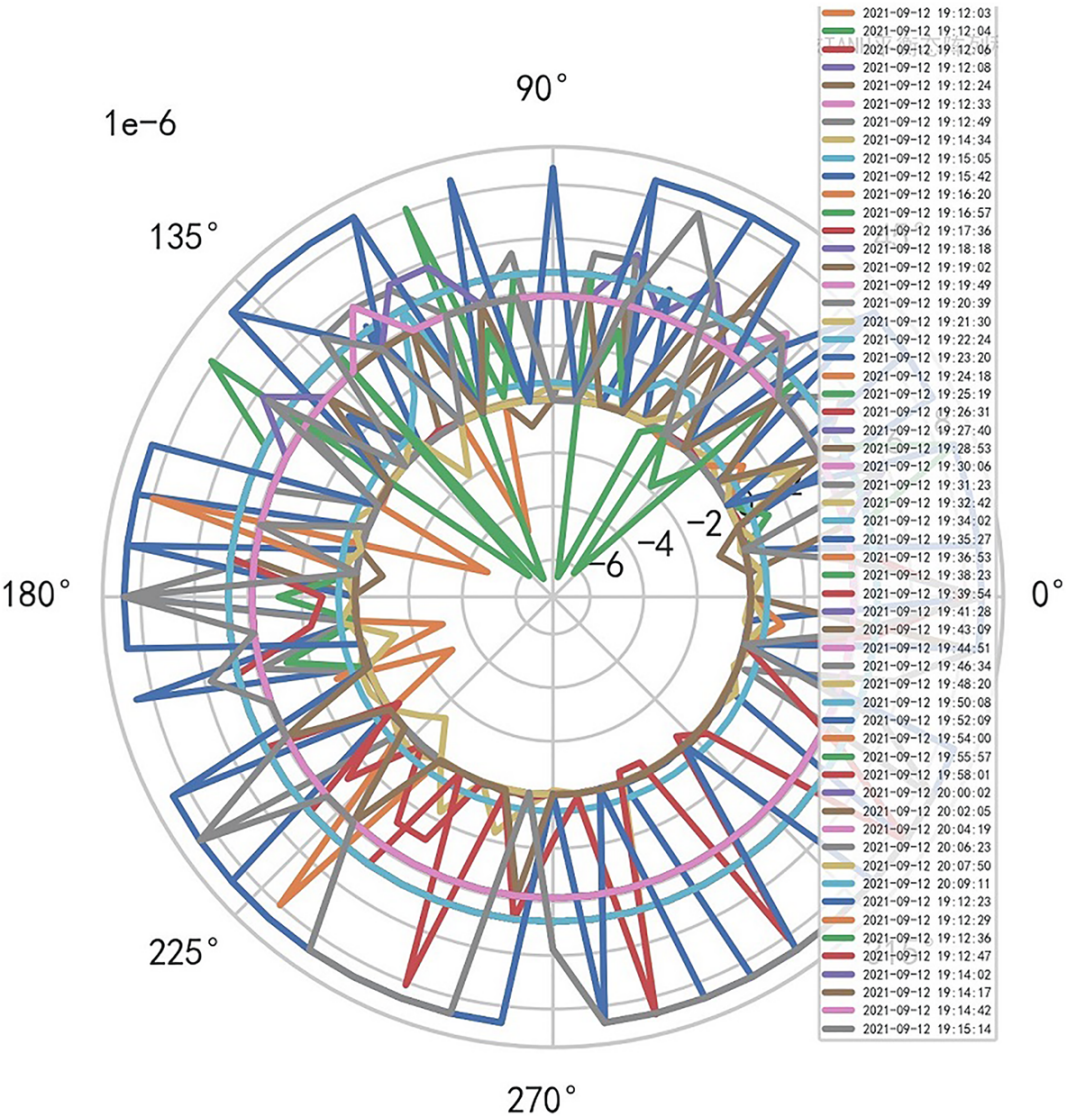
MR image definition and performance were 45.473% respectively . Comprehensive evaluation indexes: 54.398%, 41.551%, 45.473%, its core boundary is 40.01%, and the scientifically of the image are reduced.

The MR parameter is related to the machine parameter $\upomega =\left(\mathrm{TR}\otimes \mathrm{TE}\right)$, excitions_number, spacing_between_slices, Magnet_field_strength, SAR. Reference to Figs. 16, 17 and 18.

### When ${\mathbf{R}}^{-1}$ interference signals decreases, MR image clarity increases and comprehensive evaluation index increases

**Figure 19:**
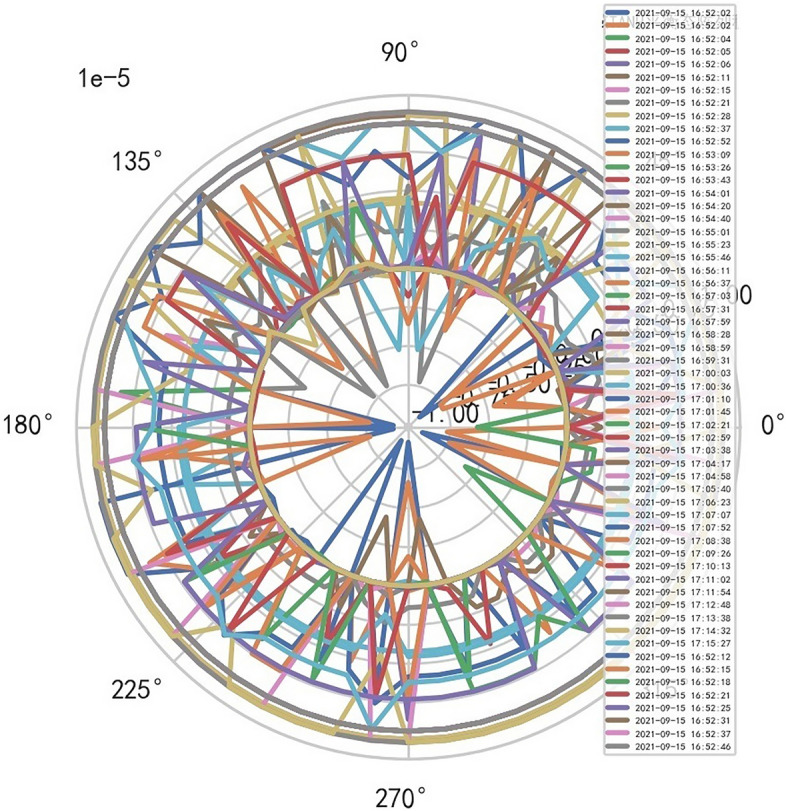
MR image definition and performance were 69.730% respectively.

**Figure 20:**
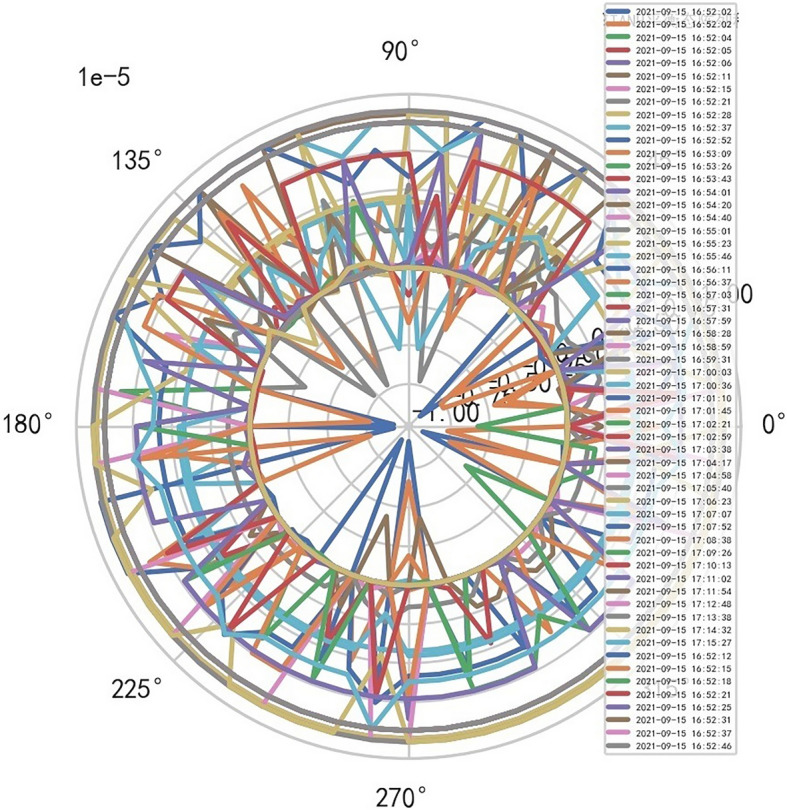
MR image definition and performance were 62.940% respectively.

**Figure 21:**
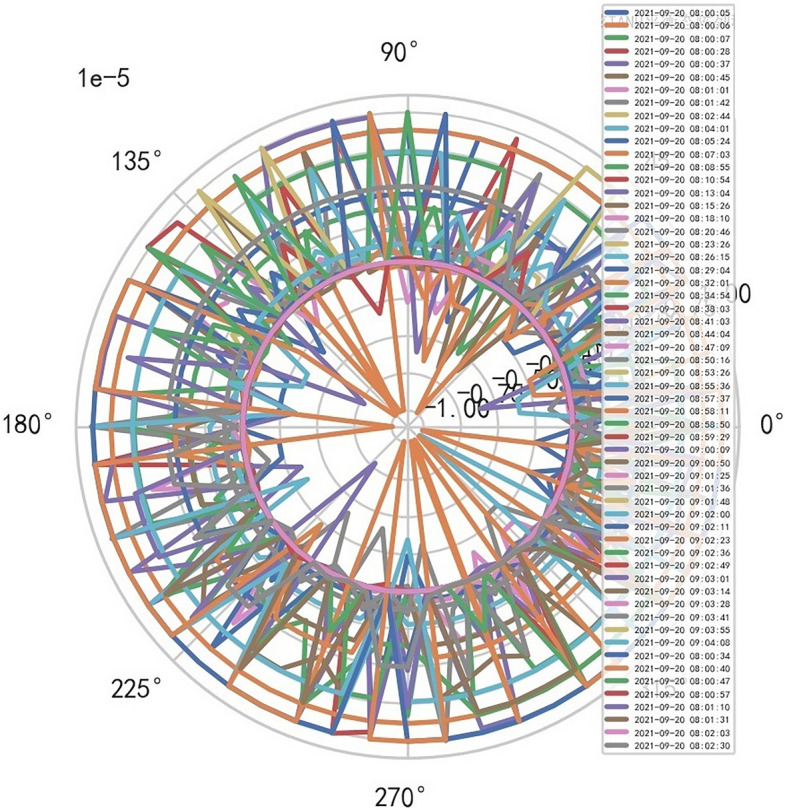
MR image definition and performance were 74.716% respectively.

Comprehensive evaluation indexes: 69.730%, 62.940%, 74.716%, its core boundary is 40.01%, and the image is more scientific. And reference to Figs. 19, 20, 21.

### MR peak SAR RF (similar to CT exposure time high-dimensional data heavy core clustering mathematical model)

If SAR > 11.2 then MR stops, when SAR drops down, start MR again. MR does not need to set the domain value, because AI Mathematical model risk control can dynamically find the domain value and boundary of various internal indicators of MR machine. This is the advantage of AI system, and adopts the most cutting-edge and advanced original innovative mathematics to combine with AI. Medical equipment management is characterized by high professionalism, high compliance requirements, diverse types and uses, scattered applicable standards and regulations, and large time and space span of equipment management^7^.

**Figure 22:**
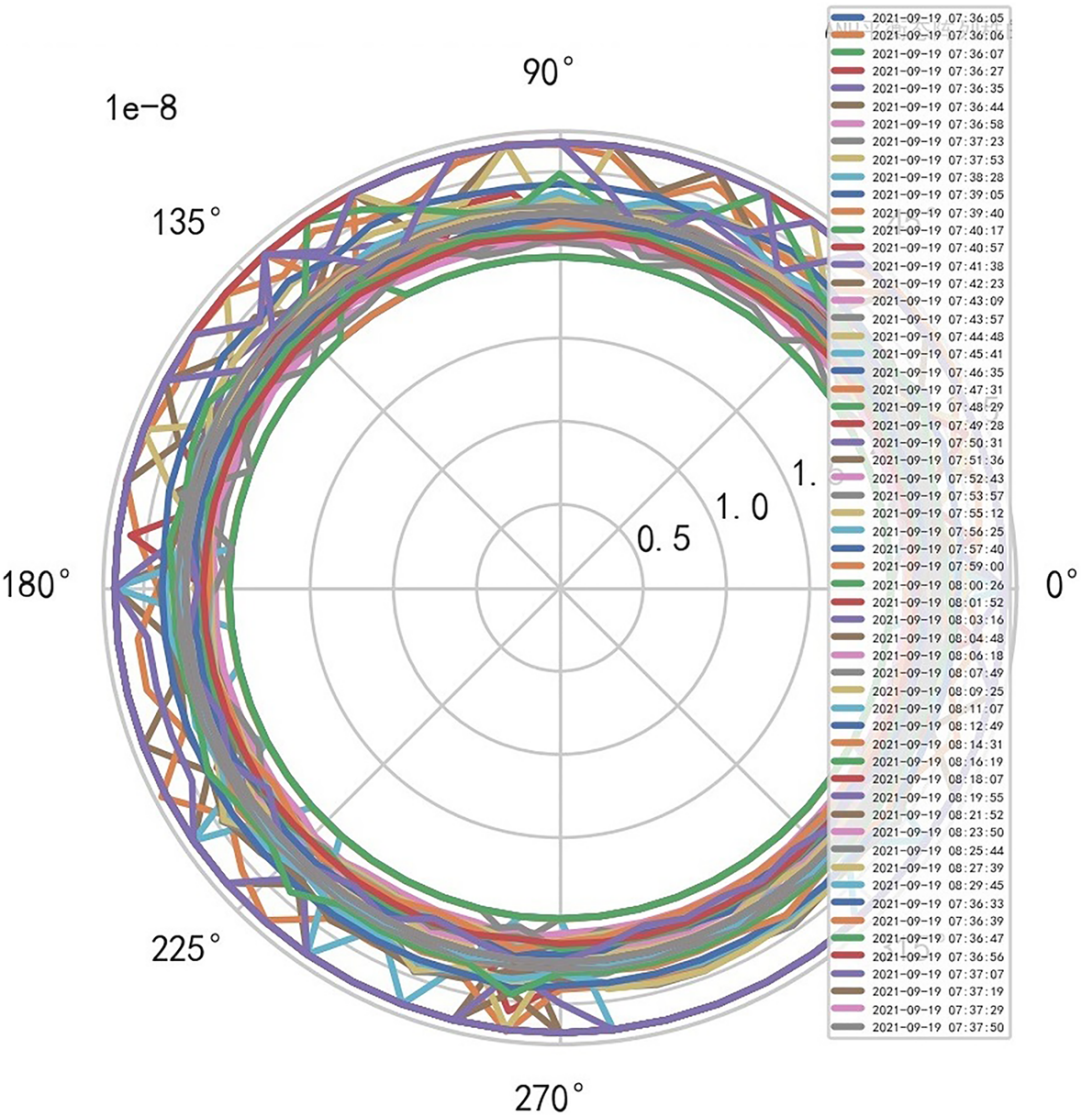
Heat capacity machine internal index and domain value of CT [weight kernel clustering tanh equilibrium big data risk control high-dimensional data] polar graph (2021-09-19 07:36:05).

**Figure 23:**
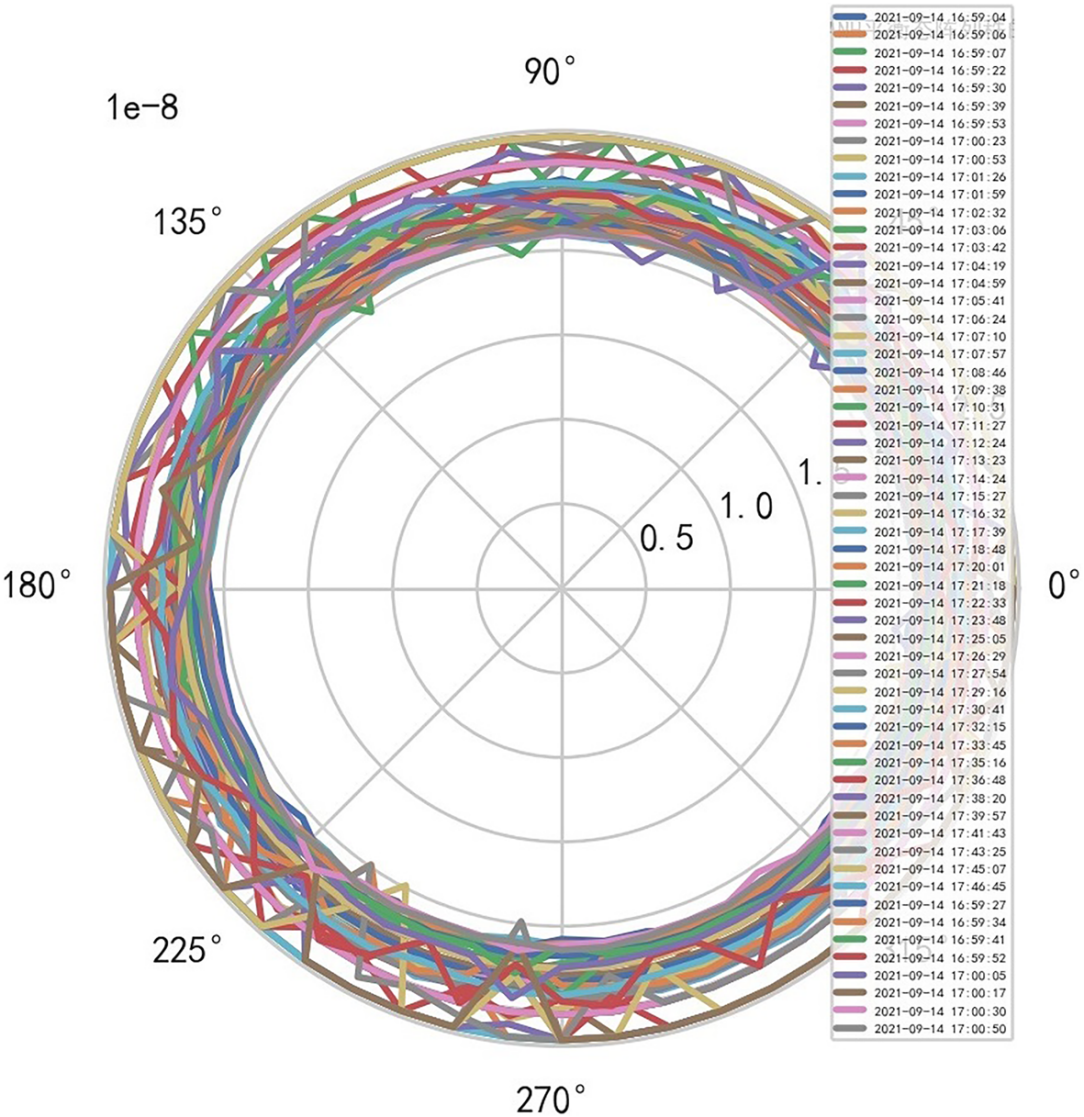
Heat capacity machine internal index and domain value of CT [weight kernel clustering tanh equilibrium big data risk control high-dimensional data] polar graph (2021-09-14 16:59:04).

AI Mathematical model risk control can automatically and dynamically find the domain values and boundaries of various medical equipment indexs, such as the domain values and boundaries of CT's heat capacity and internal indexs of the machine. And reference to Figs. 22, 23.

**Figure 24:**
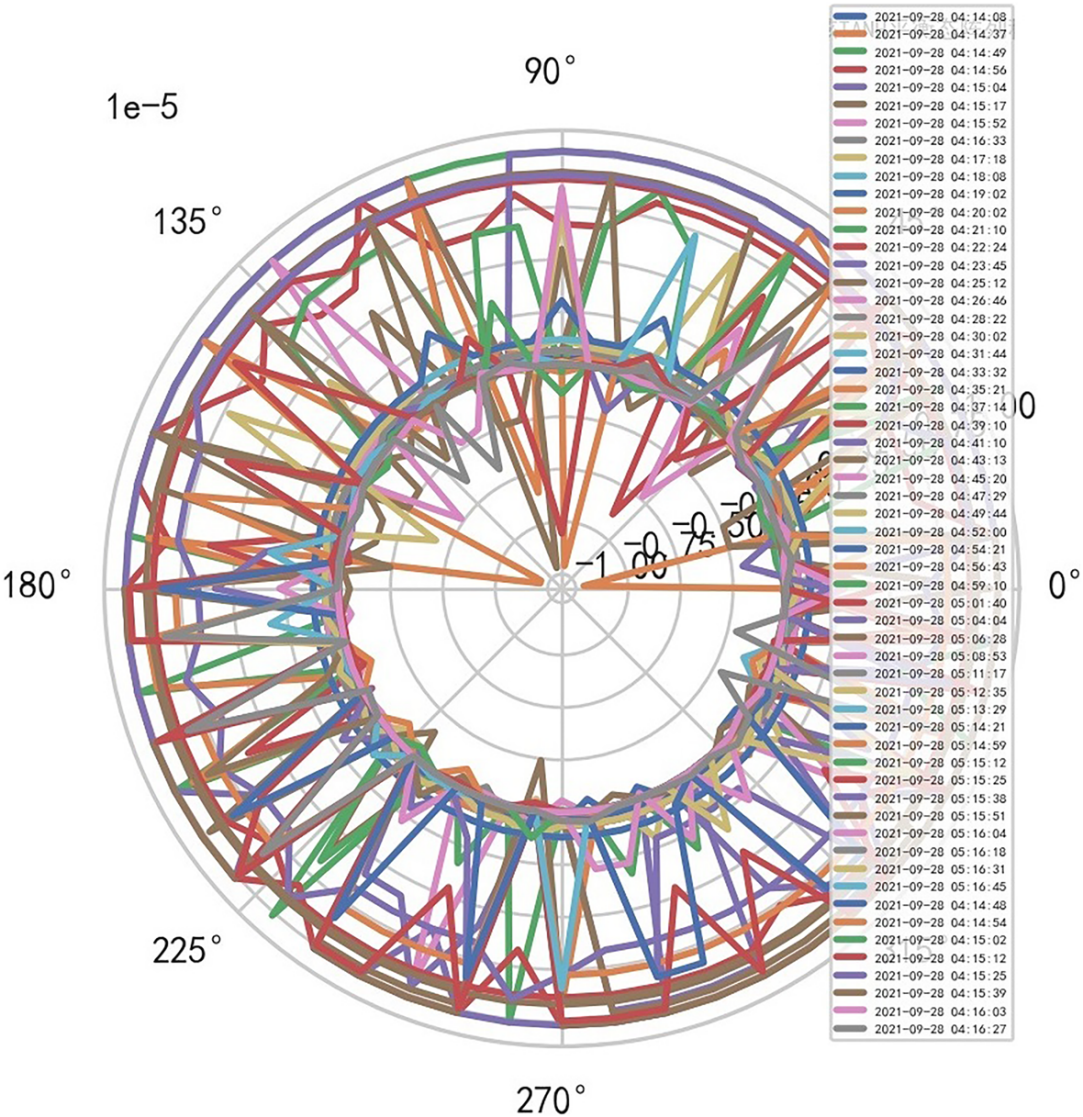
Machine internal index domain value of MR image clarity measurement [weight kernel clustering tanh equilibrium big data risk control high-dimensional data] polar graph (2021-09-28 04:14:08).

**Figure 25:**
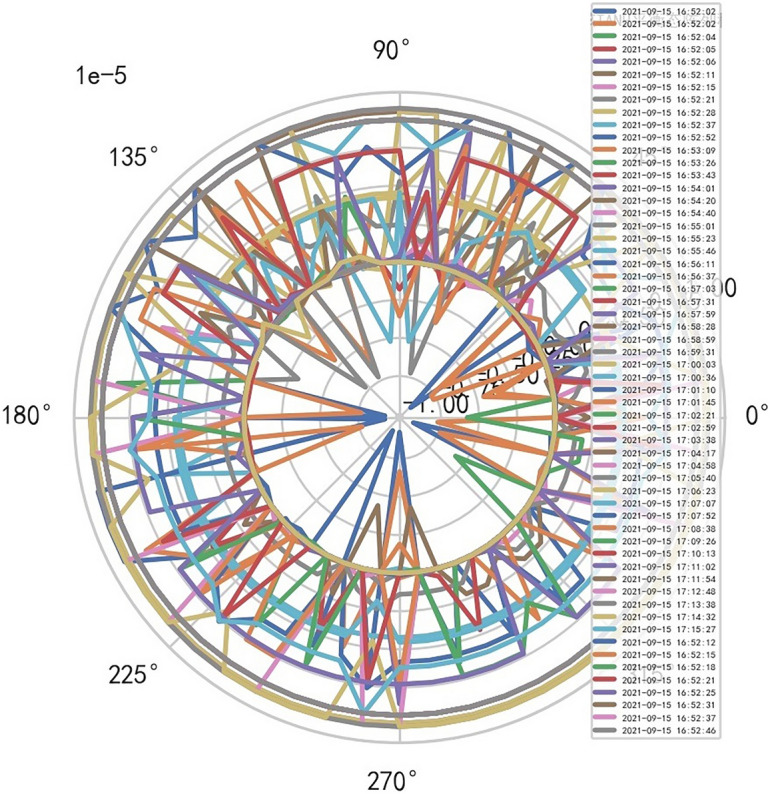
Machine internal index domain value of MR image clarity measurement [weight kernel clustering tanh equilibrium big data risk control high-dimensional data] polar graph (2021-09-15 16:52:02).

AI Mathematical model risk control automatically and dynamically finds the index domain values and boundaries of various medical equipment. Such as MR peakSAR RF, image definition, internal index domain value and boundary of the machine. And reference to Figs. 24, 25.

### Application scenario of non super flat enhanced heavy core TANH equilibrium state

**Figure 26:**
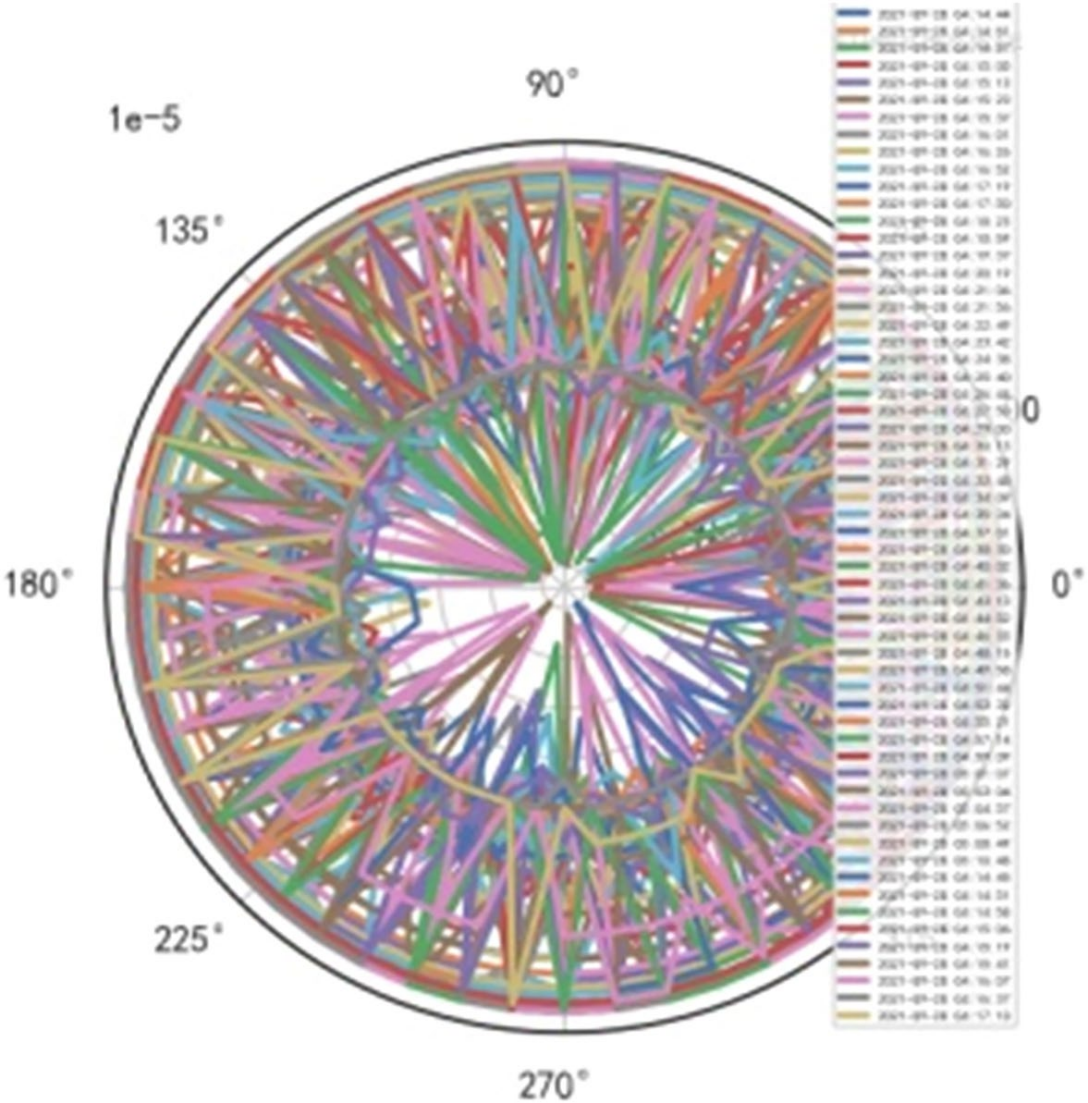
MR peak SAR heavy core clustering tanh balanced big data risk control high-dimensional data polar coordinates graph (2021-09-28 04:14:44).

**Figure 27:**
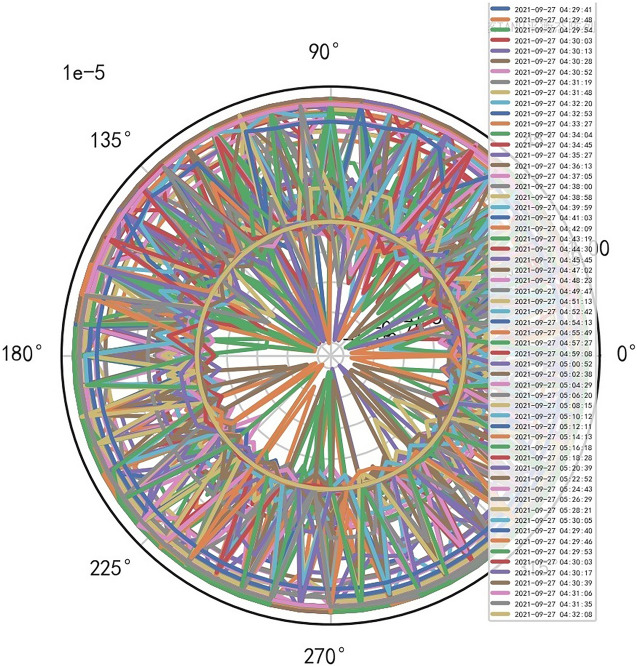
MR peak SAR heavy core clustering tanh balanced big data risk control high-dimensional data polar coordinates graph (2021-09-27 04:29:41).

**Figure 28:**
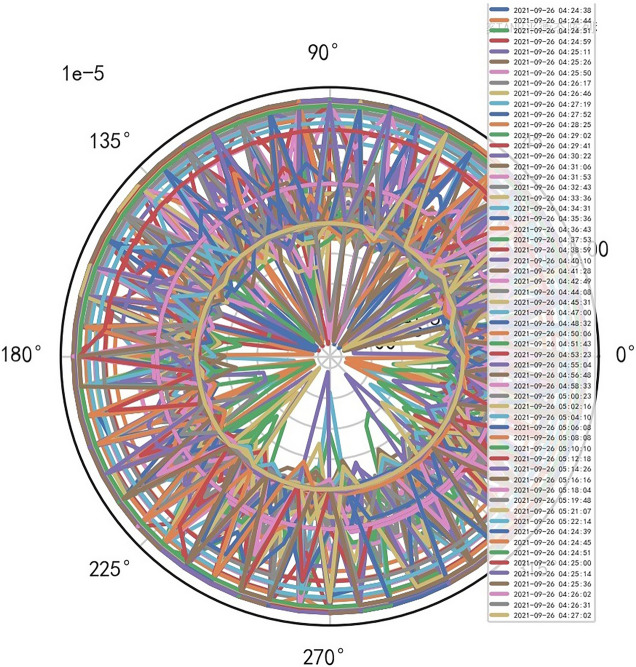
MR peak SAR heavy core clustering tanh balanced big data risk control high-dimensional data polar coordinates graph (2021-09-26 04:24:38).

Analyze the stability of DISCOVERY MR750w equipment. AI Mathematical model risk control big data found that the ductility, generality and high reliability of MR equipment DISCOVERY MR750w are also an important basis for judging whether it is a high-end MR. The reliability boundary is 40.01%, and reference to Figs. 26, 27, 28, which also reflects another important basis for high-end MR. MR peak SAR RF (core data of heavy core clustering TANH equilibrium state is similar to CT exposure time), high-dimensional signal image, and AI Mathematical model risk control image similar to CT exposure time.

### New generations of medical AI big data platform based on heavy core clustering quasi thinking iterative planning

Capture the most important quasi thinking wave curve (signal), and through the vibration of random function and AI operation, iterate and determine the condition, namely domain value. If possible, the fluctuation curve of human (thinking) brain wave signal, that is, the iterative evolution of brain like AI form on the reliability of risk control of the above large medical equipment from weak to strong^8^, can be used to provide a basis for obtaining risk control of CT large equipment. Reliability percentage data of risk control of large medical equipment are analyzed by long-time distribution curve. It can be learned and trained by KNN of AI neural network. Moreover, the heavy core data corresponding to this reliability <  + [1, 10]—[1, 10] > is KNN of dual core neural network, and the correct risk control successful data are marked through unsupervised learning.

## The clustering lens effect of tanh equilibrium heavy core of super flat weakened heavy core and non super flat enhanced heavy core

### Enhancement of TANH equilibrium lens effect by heavy core hypersphere

The distribution of particles in high dimensions can be observed from the heavy core lens effect on the hypersphere with nuclear magnetic resonance particle energy distribution^9^.

**Figure 29:**
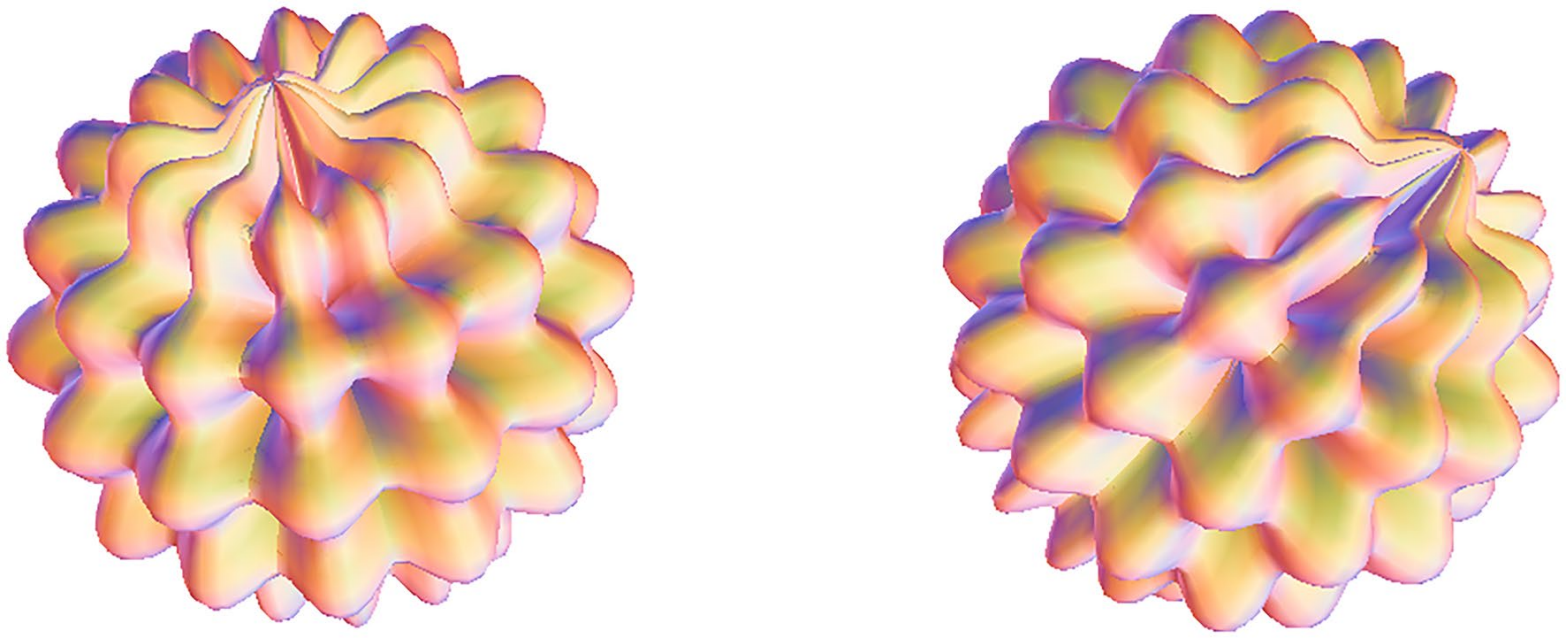
The RBF characteristic space of heavy core hypersphere enhancing tanh equilibrium lens effect is the Taylor series expansion of complex variable hypersphere kernel function.

**Figure 30:**
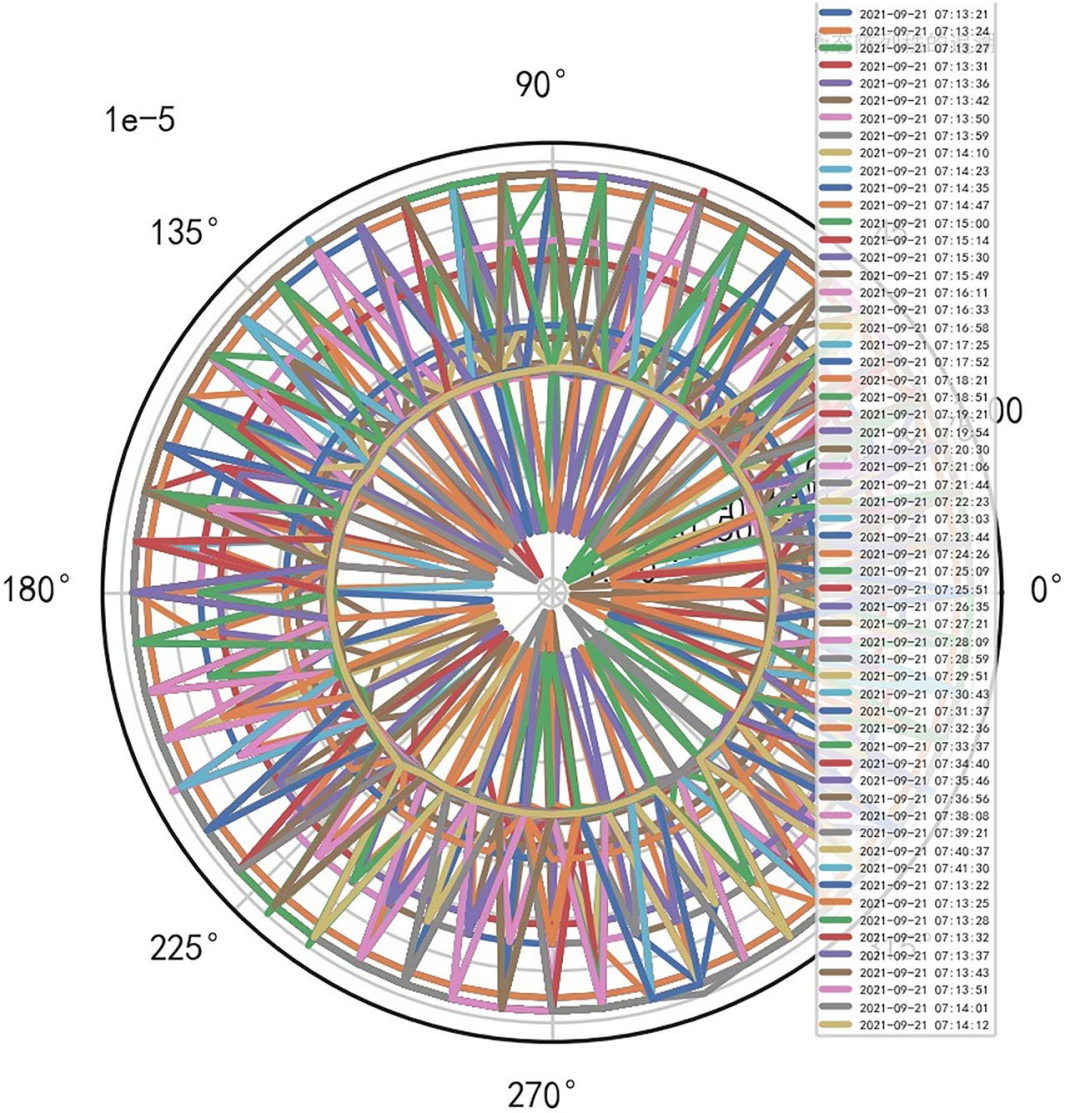
CT tubes exposure time big data heavy core clustering high-dimensional information maps.

**Figure 31:**
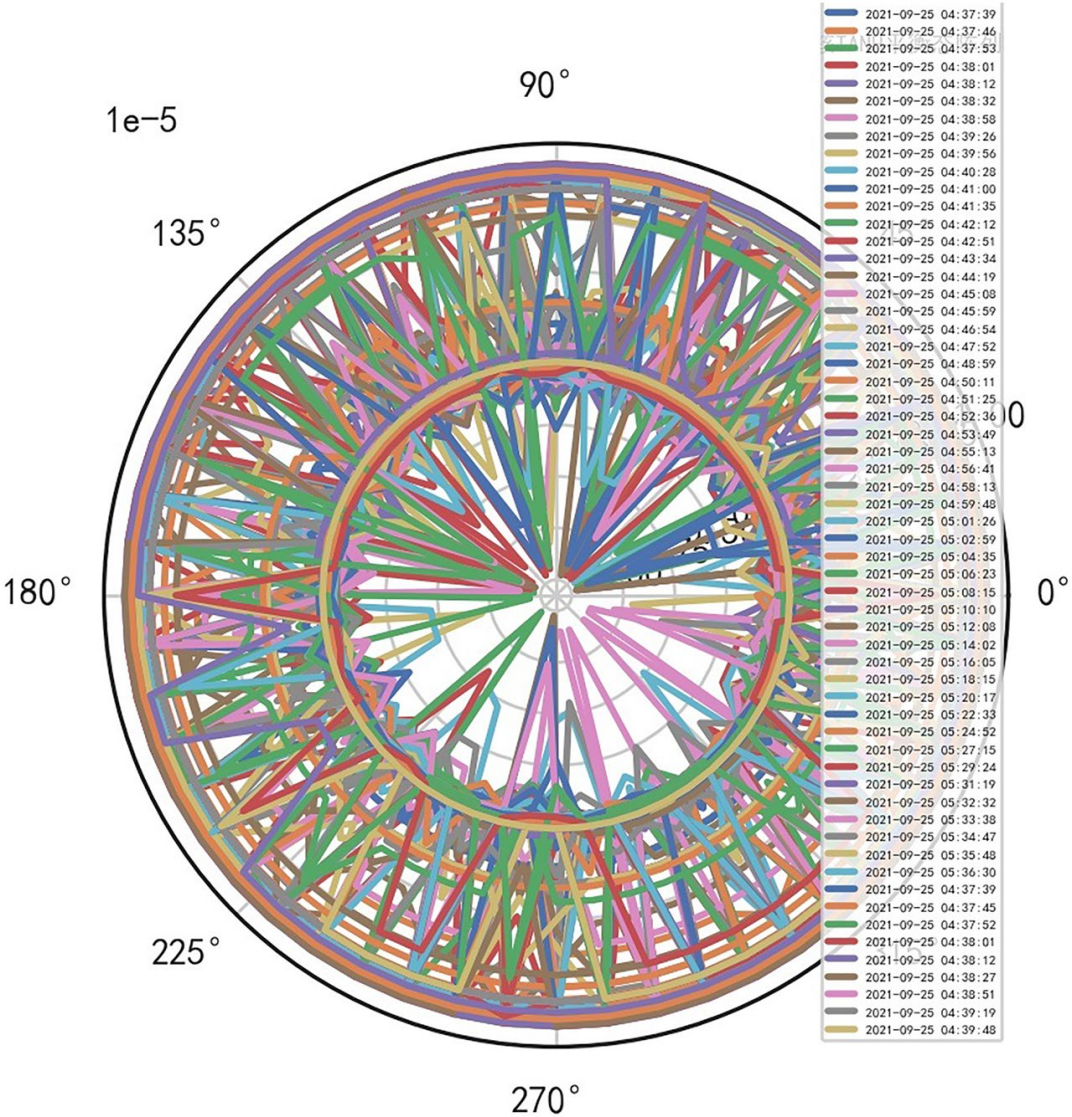
MR peak SAR big data heavy core clustering high-dimensional information maps.

$K=1-\frac{{\left[Ker\right]}_{{\left({A}_{i}\otimes {B}_{i}\right)}_{*}^{{\Delta }^{2}}}}{{S}^{2}}+\dots ,{\left[Ker\right]}_{{\left({A}_{i}\otimes {B}_{i}\right)}_{*}^{{\Delta }^{2}}}$ is the *s*-1 kernel of the 2-order hypersphere, so the *s*-1-dimensional kernel of the higher-order (2-order) hypersphere ${KER}_{core}^{P}$ kernel of the complex variable heavy kernel can be derived. And reference to Figs. 29, 30, 31.

$${KER}_{P}^{{i\cdot \left(Sin,Cos\right)}^{2}}=\left[{Sin}^{2}\left(\sum_{i=2}^{m}{A}_{i}+\sum_{i=1}^{m}i\cdot \frac{{A}_{i}}{2}\right)+{Cos}^{2}\left(\sum_{i=2}^{m}{B}_{i}+\sum_{i=1}^{m}i\cdot \frac{{B}_{i}}{2}\right)\right]$$

8 $$\left\{\begin{array}{c}K=1-i\cdot \frac{{\lambda }_{i}{\left[{KER}_{P}^{{i\cdot \left(Sin,Cos\right)}^{2}}\right]}^{s-1}}{{S}^{2}}-{i}^{2}\frac{{\lambda }_{i+1}{\left[{KER}_{P}^{{i\cdot \left(Sin,Cos\right)}^{2}}\right]}^{s-2}}{{S}^{2}}+{i}^{3}\cdot \frac{{\lambda }_{i+2}{\left[{KER}_{P}^{{i\cdot \left(Sin,Cos\right)}^{2}}\right]}^{s-3}}{{S}^{2}}-\dots \\ \\ K=1-\frac{{\lambda }_{i}{\left[{KER}_{P}^{{i\cdot \left(Cos,-Sin\right)}^{2}}\right]}^{s-1}}{{S}^{2}}-\frac{{\lambda }_{i+1}{\left[{KER}_{P}^{{i\cdot \left(Cos,-Sin\right)}^{2}}\right]}^{s-2}}{{S}^{2}}+\frac{{\lambda }_{i+2}{\left[{KER}_{P}^{{i\cdot \left(Cos,-Sin\right)}^{2}}\right]}^{s-3}}{{S}^{2}}-\dots \end{array}\right.$$

**Figure 32:**
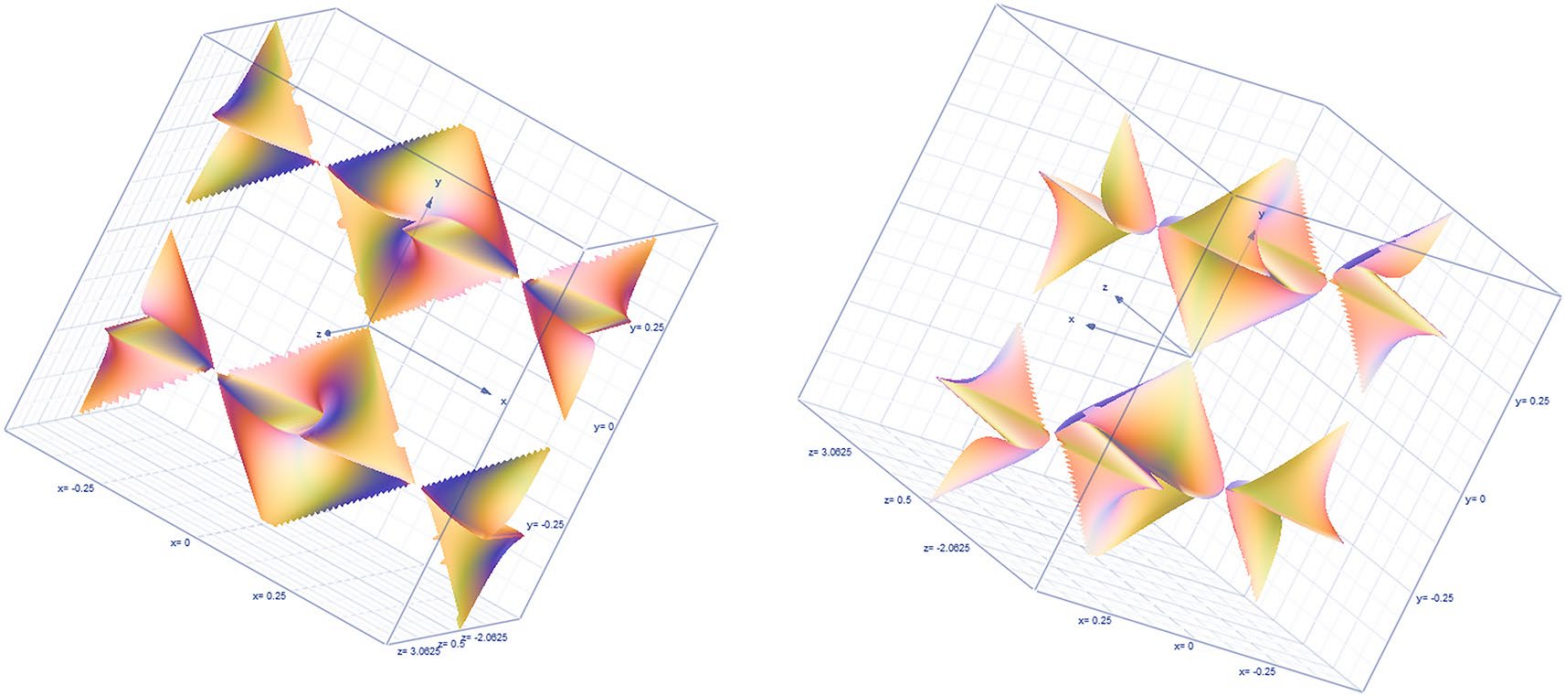
Hierarchical fuzzy clustering system based on differential incremental equilibrium theory, the orthogonal subkernel quadratic convolution norm of heavy core clustering lens.

The super flat structure of the weakened heavy core clustering data is enlarged slightly, and the small fluctuation heavy core is constructed, which has the effect of lens amplification^10^. And reference to Fig. 32,

$$\sum_{j=1}^{m}\sum_{i=1}^{m}{\nabla }_{ker}{P}_{(2)}^{+}\left({x}_{i},{y}_{j}\right)={\left[\frac{{}_{G}{P}_{{x}_{i}}^{2}}{{Cos}^{2}\left(\sum_{i=2}^{m}{x}_{i}+\sum_{i=1}^{m}i\cdot {x}_{i}/2\right)}+\frac{{}_{G}{P}_{{y}_{j}}^{2}}{{Sin}^{2}\left(\sum_{j=2}^{m}{y}_{j}+\sum_{j=1}^{m}j\cdot {y}_{j}/2\right)}\right]}_{(2)}^\frac{1}{2}$$

9 $$\times {arctg\left[\frac{Sin\left(\sum_{j=2}^{m}{y}_{j}+\sum_{j=1}^{m}j\cdot {y}_{j}/2\right)}{Cos\left(\sum_{i=2}^{m}{x}_{i}+\sum_{i=1}^{m}i\cdot {x}_{i}/2\right)}\right]}_{P}$$

10 $$1={\left[\frac{{}_{G}{P}_{{x}_{i}}^{2}}{{Cos}^{2}\left(\sum_{i=2}^{m}{x}_{i}+\sum_{i=1}^{m}i\cdot {x}_{i}/2\right)}+\frac{{}_{G}{P}_{{y}_{j}}^{2}}{{Sin}^{2}\left(\sum_{j=2}^{m}{y}_{j}+\sum_{j=1}^{m}j\cdot {y}_{j}/2\right)}\right]}_{(2)}^\frac{1}{2}{arctg\left[\frac{Sin\left(\sum_{j=2}^{m}{y}_{j}+\sum_{j=1}^{m}j\cdot {y}_{j}/2\right)}{Cos\left(\sum_{i=2}^{m}{x}_{i}+\sum_{i=1}^{m}i\cdot {x}_{i}/2\right)}\right]}_{P}$$

### Heat capacity weaken the TANH equilibrium state of the heavy core to the series expansion of the restricted RBF hypersphere heavy kernel function

Compute the comparative analysis of the percentage of iCT256 and uCT528 in the comprehensive evaluation of reliability. Through heavy kernel clustering lens orthogonal subkernel quadratic convolution, and form an embedded program. Making the uCT528 approaches the high-end iCT256.

**Figure 33:**
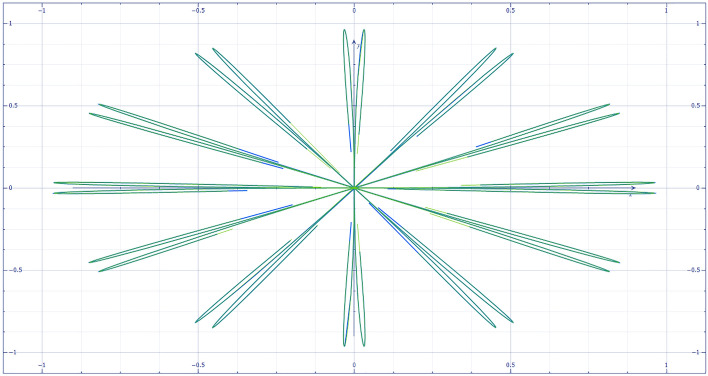
Orthogonal subkernel of heavy core clustering lens quadratic convolution norm RBF gradient s-dimensional subkernel function—harmonic function image of topological space.

According to the analysis of the following formula, the increment index of comprehensive evaluation directly affecting reliability is $arctg\phi \left({x}_{i},{y}_{j}\right)$. Therefore, the reliability increment index of uCT528 is controlled between (12.5%, 25%).${if arctg\left[\frac{Sin\left(\sum_{j=2}^{m}{y}_{j}+\sum_{j=1}^{m}i\cdot {y}_{j}/2\right)}{Cos\left(\sum_{i=2}^{m}{x}_{i}+\sum_{i=1}^{m}i\cdot {x}_{i}/2\right)}\right]}_{P}\in \left(\frac{\pi }{4},\frac{\pi }{2}\right)$ . And reference to Fig. 33.

11 $$1={\left[\frac{{}_{P}{G}_{{x}_{i}}^{2}}{{Cos}^{2}\left(\sum_{i=2}^{m}{x}_{i}+\sum_{i=1}^{m}i\cdot {x}_{i}/2\right)}+\frac{{}_{P}{G}_{{y}_{i}}^{2}}{{Sin}^{2}\left(\sum_{j=2}^{m}{y}_{j}+\sum_{j=1}^{m}j\cdot {y}_{j}/2\right)}\right]}_{(2)}^\frac{1}{2}\times {arctg\left[\frac{Sin\left(\sum_{j=2}^{m}{y}_{j}+\sum_{j=1}^{m}i\cdot {y}_{j}/2\right)}{Cos\left(\sum_{i=2}^{m}{x}_{i}+\sum_{i=1}^{m}i\cdot {x}_{i}/2\right)}\right]}_{P}$$

## Conclusion

Contactless medical equipment AI big data risk control and quasi thinking iterative planning. For the first time, this kind of general form is adopted to automatically carry out machine internal information big data AI Mathematical model risk control for all large equipment in the hospital, and it has well portability. Adding different equipment into the system will automatically detect and display the information and data of the internal operation of the risk control machine. AI big data risk control and quasi thinking iterative planning cross platform web software will be started automatically, and display the risk control data and image display of its whole life cycle. It has a predictable risk control system, that is, a big data intelligent platform for establishing the overall predictability maintenance system of medical institutions.

## Supplementary Information

Supplementary Information 1.

Supplementary Information 2.

## Data Availability

All data generated or analysed during this study are included in this published article [and its supplementary information files].
