# Supplementary material for: Contactless medical equipment AI big data risk control and quasi thinking iterative planning
Source: Sci Rep. 2022 Sep 3;12:15039. doi: 10.1038/s41598-022-18724-5 (PMC9440912; doi:10.1038/s41598-022-18724-5)
Supplement: Supplementary file 1 — Supplementary Information 1. [file 41598_2022_18724_MOESM1_ESM.pdf]

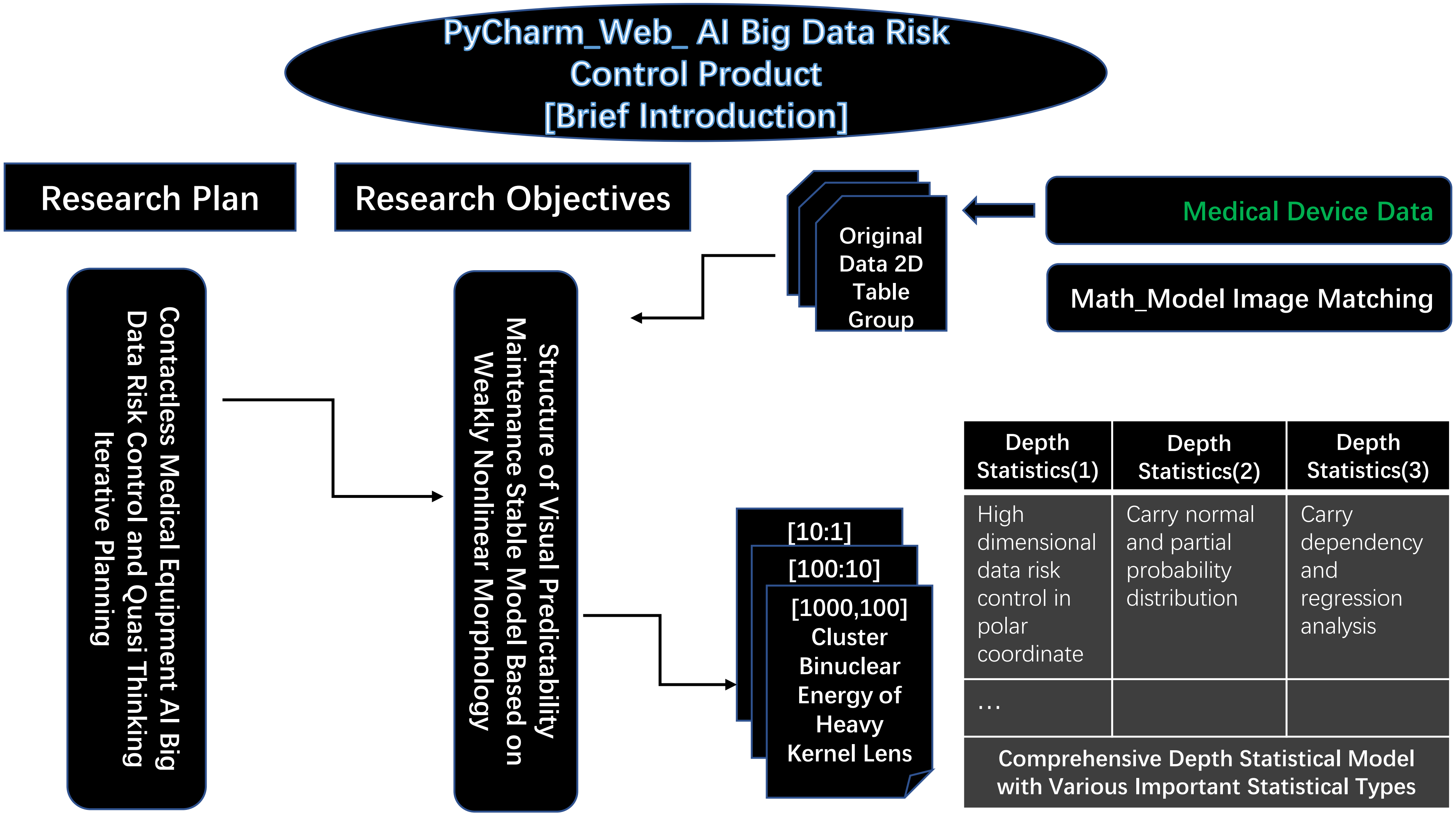
PyCharm_Web_ AI Big Data Risk Control Product [Brief Introduction]
Research Plan
Research Objectives
Original Data 2D Table Group
Medical Device Data
Math_Model Image Matching
Contactless Medical Equipment AI Big Data Risk Control and Quasi Thinking Iterative Planning
Structure of Visual Predictability Maintenance Stable Model Based on Weakly Nonlinear Morphology
[10:1]
[100:10]
[1000,100] Cluster Binuclear Energy of Heavy Kernel Lens
Depth Statistics(1)
Depth Statistics(2)
Depth Statistics(3)
High dimensional data risk control in polar coordinate
Carry normal and partial probability distribution
Carry dependency and regression analysis
…
Comprehensive Depth Statistical Model with Various Important Statistical Types

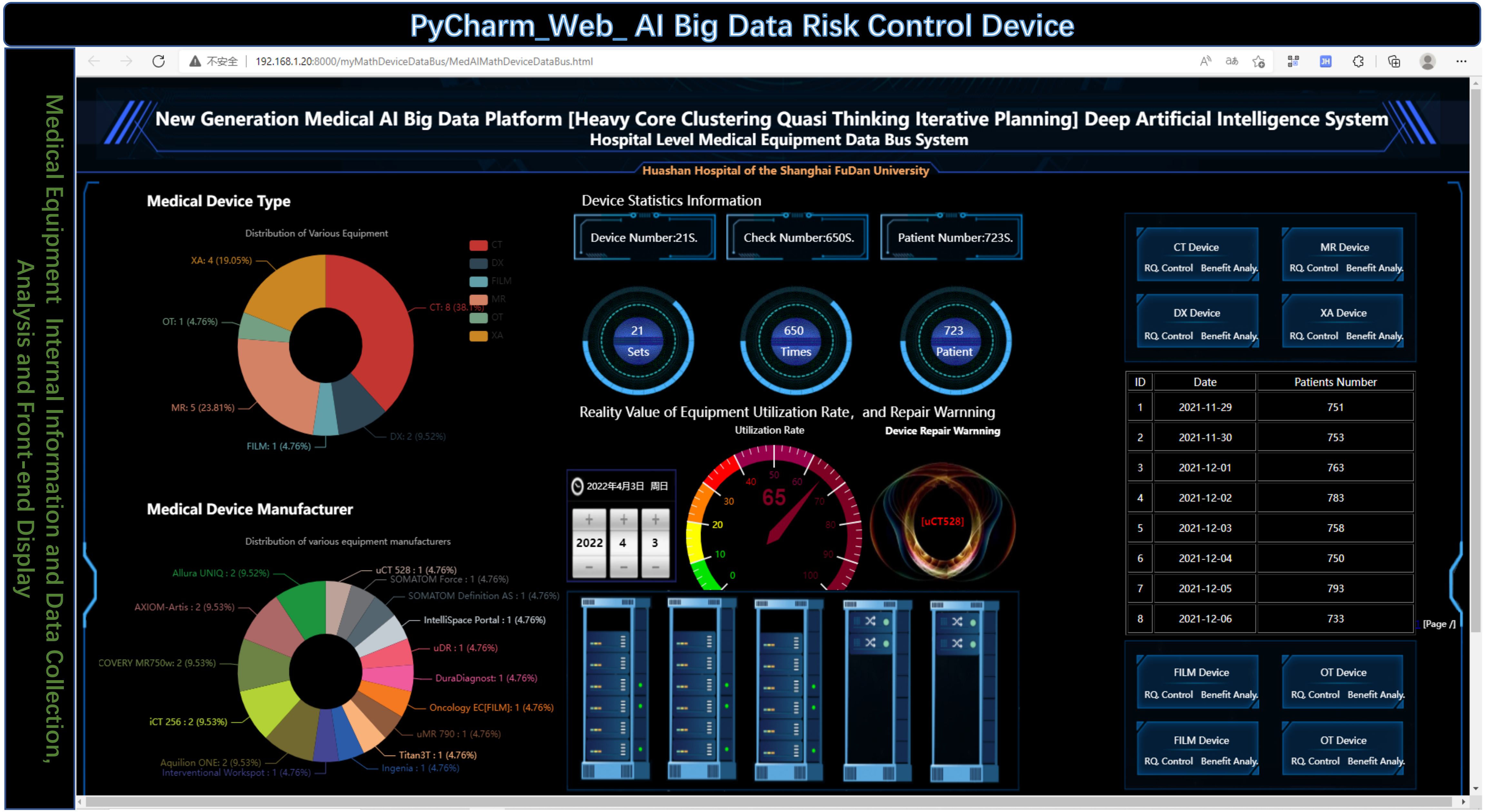
PyCharm_Web_ AI Big Data Risk Control Device
Medical Equipment Internal Information and Data Collection, Analysis and Front-end Display
192.168.1.20:8000/myMathDeviceDataBus/MedAIMathDeviceDataBus.html
New Generation Medical AI Big Data Platform [Heavy Core Clustering Quasi Thinking Iterative Planning] Deep Artificial Intelligence System
Hospital Level Medical Equipment Data Bus System
Huashan Hospital of the Shanghai FuDan University
Medical Device Type
Distribution of Various Equipment
CT
DX
FILM
MR
OT
XA
XA: 4 (19.05%)
CT: 8 (38.1%)
OT: 1 (4.76%)
MR: 5 (23.81%)
FILM: 1 (4.76%)
DX: 2 (9.52%)
Medical Device Manufacturer
Distribution of various equipment manufacturers
Allura UNIQ : 2 (9.52%)
uCT 528 : 1 (4.76%)
SOMATOM Force : 1 (4.76%)
SOMATOM Definition AS : 1 (4.76%)
AXIOM-Artis : 2 (9.53%)
IntelliSpace Portal : 1 (4.76%)
uDR : 1 (4.76%)
COVERY MR750w: 2 (9.53%)
DuraDiagnost: 1 (4.76%)
Oncology EC[FILM]: 1 (4.76%)
iCT 256 : 2 (9.53%)
uMR 790 : 1 (4.76%)
Titan3T : 1 (4.76%)
Aquilion ONE: 2 (9.53%)
Interventional Workspot : 1 (4.76%)
Ingenia : 1 (4.76%)
Device Statistics Information
Device Number:21S.
Check Number:650S.
Patient Number:723S.
21 Sets
650 Times
723 Patient
Reality Value of Equipment Utilization Rate, and Repair Warnning
Utilization Rate
Device Repair Warnning
2022年4月3日 周日
2022 4 3
65
[uCT528]
CT Device
RQ. Control Benefit Analy.
MR Device
RQ. Control Benefit Analy.
DX Device
RQ. Control Benefit Analy.
XA Device
RQ. Control Benefit Analy.
ID Date Patients Number
1 2021-11-29 751
2 2021-11-30 753
3 2021-12-01 763
4 2021-12-02 783
5 2021-12-03 758
6 2021-12-04 750
7 2021-12-05 793
8 2021-12-06 733
[Page /]
FILM Device
RQ. Control Benefit Analy.
OT Device
RQ. Control Benefit Analy.
FILM Device
RQ. Control Benefit Analy.
OT Device
RQ. Control Benefit Analy.

# PyCharm_Web_ AI Big Data Risk Control Product [Brief Introduction]

**Carry Big Data Comprehensive In-depth Statistics of Various Important Statistical Types and Internal Information Model of AI Risk Control Medical Equipment**

High Dimensional Data Risk Control in Polar Coordinate

Carry Normal and Partial Probability Distribution

Carry Dependency and Regression Analysis

......

Energy Fluctuation of Edge Lens with Double Core and Heavy Kernel

Structure and Function of Cluster Lens Mesh Screen [300000 / S Vibration, 30 Concurrent Operations]

First Set of Grid Primary Screening Structure [1000:100]

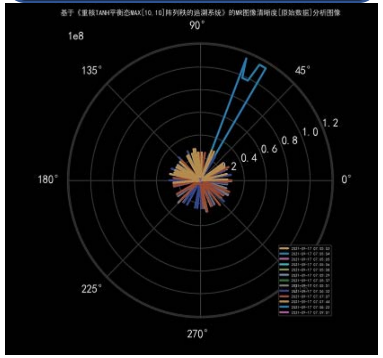

Second Set of Grid Screen Structure [100:10]

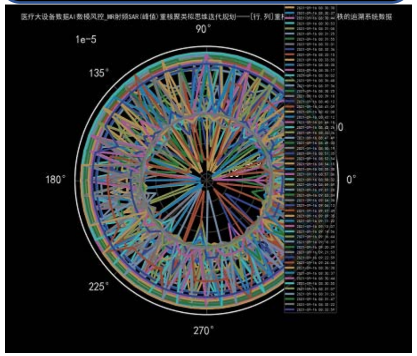

The Third Set of Grid Screen Structure [10:1] Core Lens

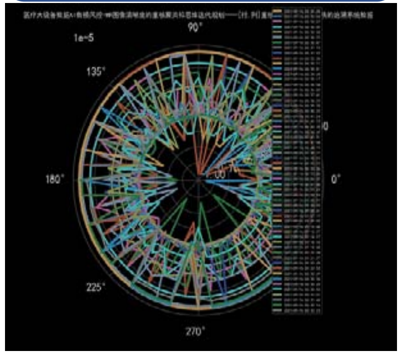

......

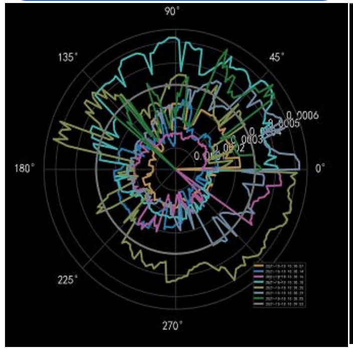

**Data Exception**

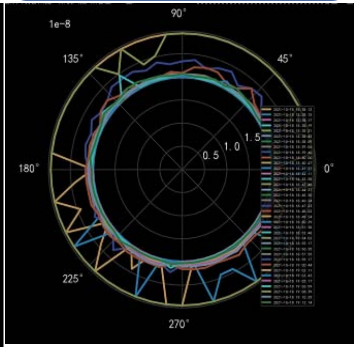

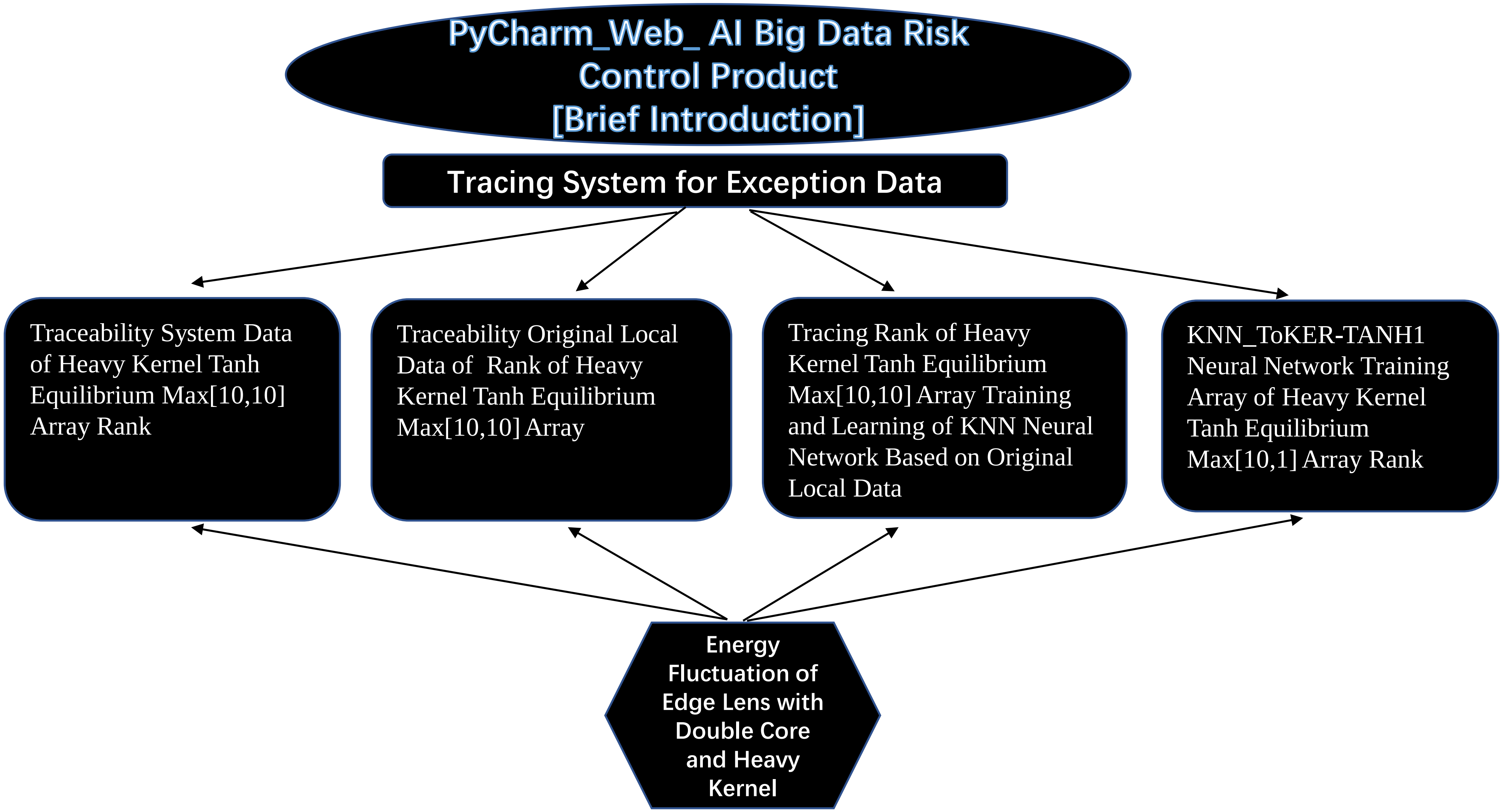
PyCharm_Web_ AI Big Data Risk Control Product [Brief Introduction]
Tracing System for Exception Data
Traceability System Data of Heavy Kernel Tanh Equilibrium Max[10,10] Array Rank
Traceability Original Local Data of  Rank of Heavy Kernel Tanh Equilibrium Max[10,10] Array
Tracing Rank of Heavy Kernel Tanh Equilibrium Max[10,10] Array Training and Learning of KNN Neural Network Based on Original Local Data
KNN_ToKER-TANH1 Neural Network Training Array of Heavy Kernel Tanh Equilibrium Max[10,1] Array Rank
Energy Fluctuation of Edge Lens with Double Core and Heavy Kernel

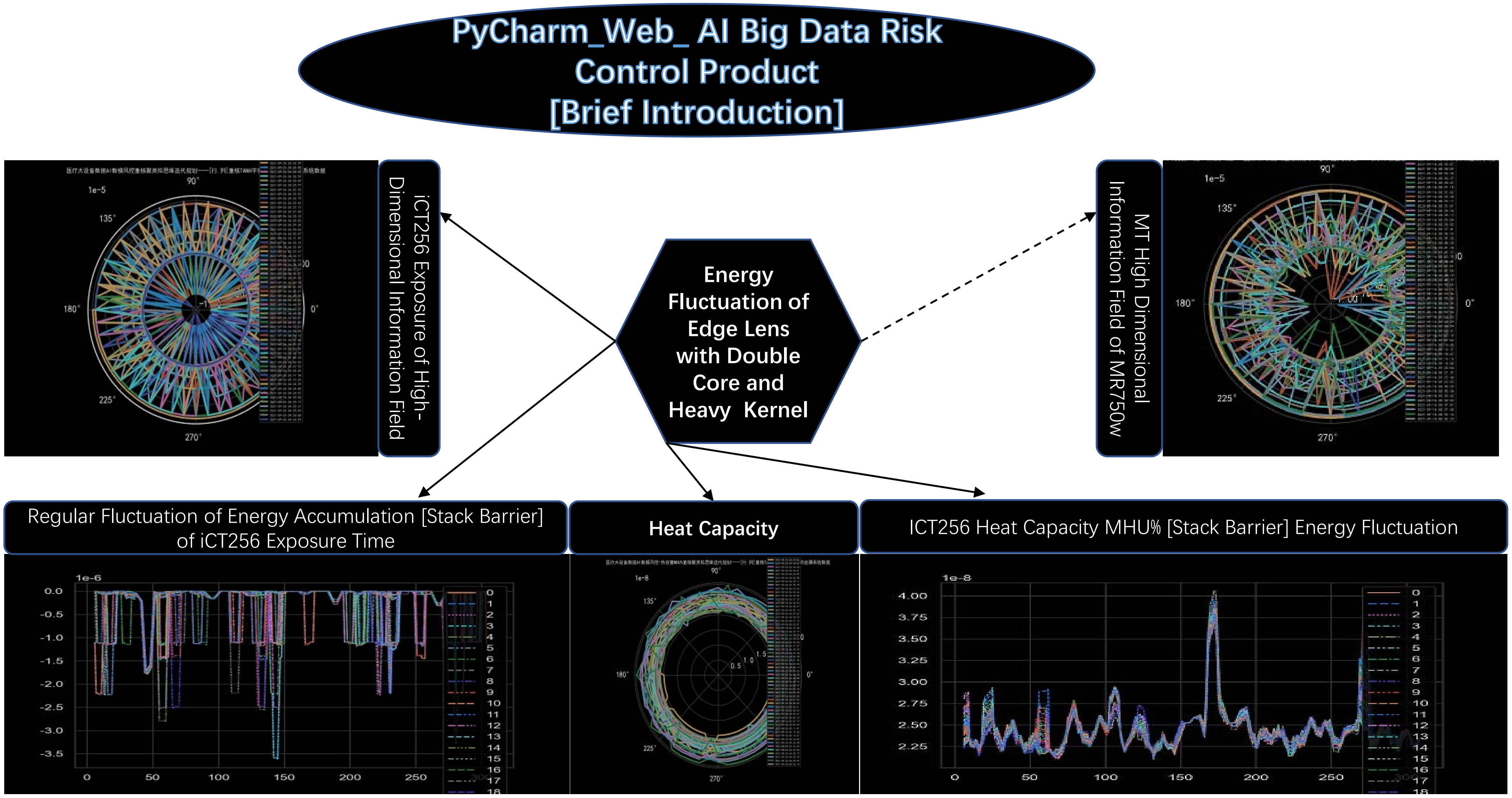
PyCharm_Web_ AI Big Data Risk Control Product [Brief Introduction]
iCT256 Exposure of High-Dimensional Information Field
Energy Fluctuation of Edge Lens with Double Core and Heavy Kernel
MT High Dimensional Information Field of MR750w
Regular Fluctuation of Energy Accumulation [Stack Barrier] of iCT256 Exposure Time
Heat Capacity
ICT256 Heat Capacity MHU% [Stack Barrier] Energy Fluctuation

# PyCharm_Web_ AI Big Data Risk Control Product [Brief Introduction]

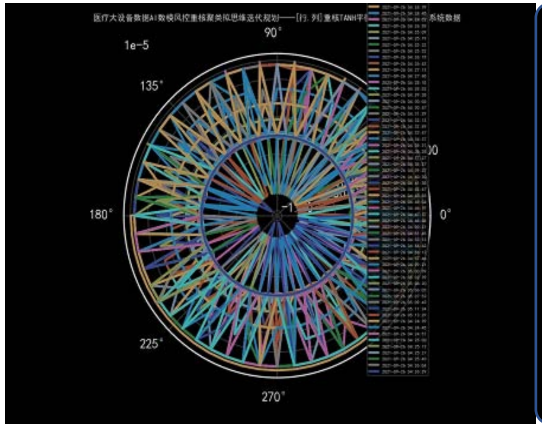


iCT256 Exposure of High-Dimensional Information Field

**Energy Fluctuation of Edge Lens with Double Core and Heavy Kernel**

MT High Dimensional Information Field of MR750w

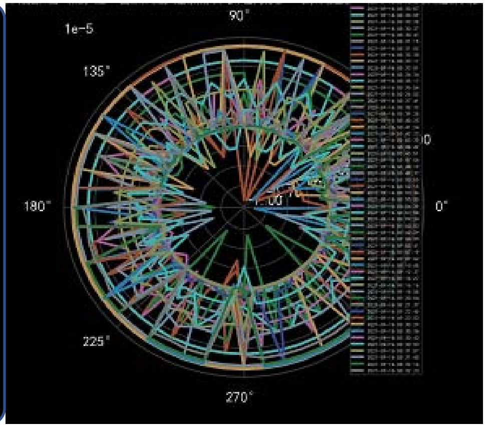


MR 750w RF SAR Energy Accumulation [Stack Barrier] Regular Fluctuation

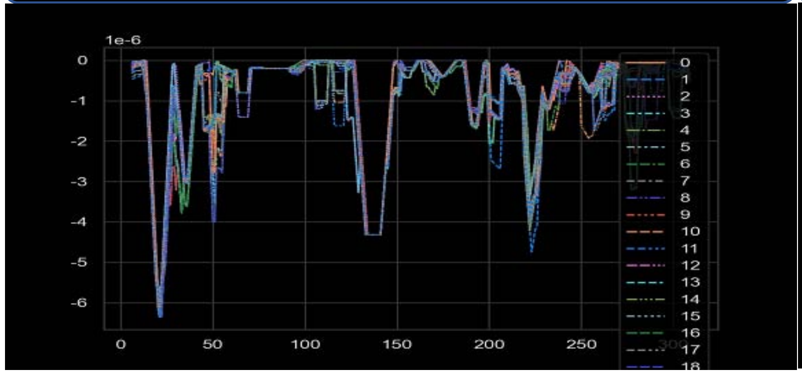


**MR Original Data**

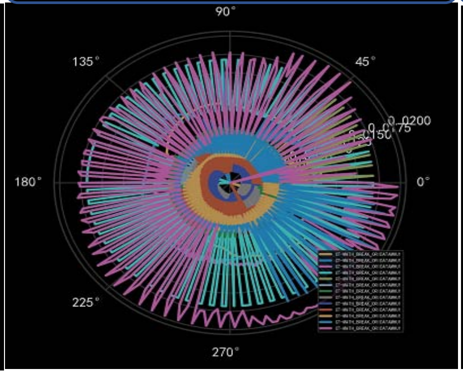


Energy Fluctuation of MR 750w Image Definition

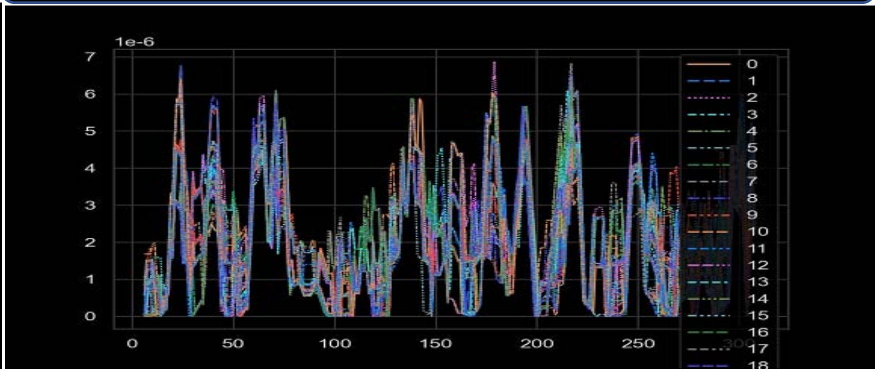

# PyCharm_Web_ AI Big Data Risk Control Product [Brief Introduction]

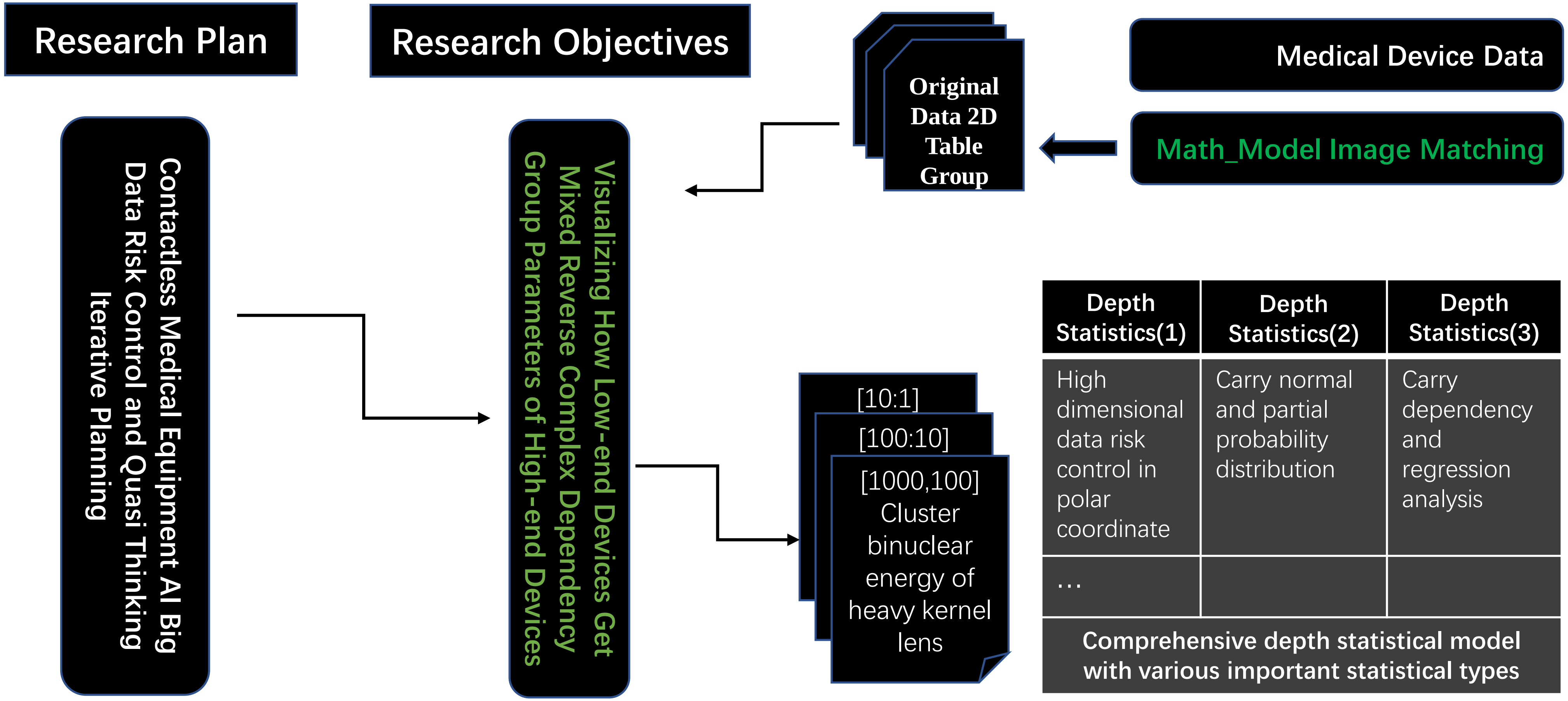


| Depth Statistics(1) | Depth Statistics(2) | Depth Statistics(3) |
|---|---|---|
| High dimensional data risk control in polar coordinate | Carry normal and partial probability distribution | Carry dependency and regression analysis |
| … | | |
| Comprehensive depth statistical model with various important statistical types | | |

# PyCharm_Web_ AI Big Data Risk Control Product [Brief Introduction]

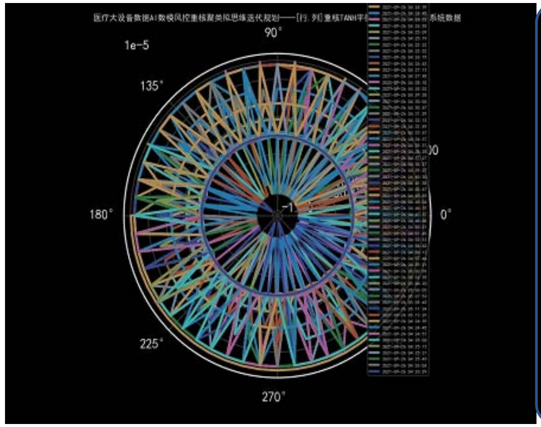


**High-end ICT256 Exposes High-Dimensional Information Field**

Energy Fluctuation of Edge Lens with Double Core and Heavy Kernel, **Repair Complex Dependent Parameter Group of Low-end to High-end CT Equipment**

**Low-end UCT528 Exposes High-Dimensional Information Field**

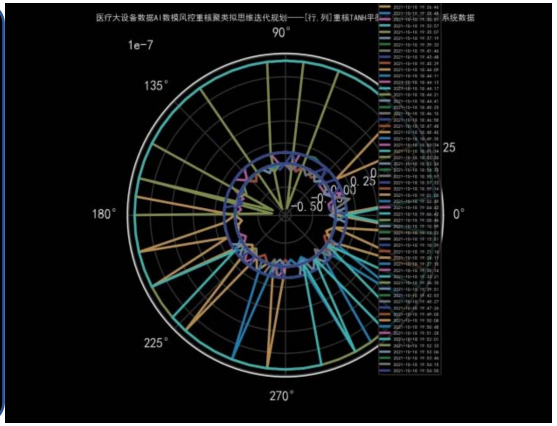


**High-end ICT256 MHU%**

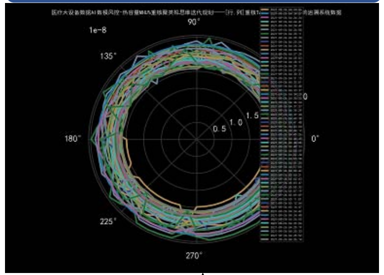


**Low-end UCT528 MHU%**

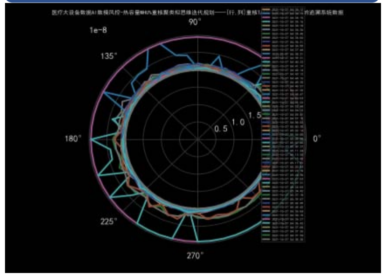


Energy Accumulation [Stack Barrier] Regular Fluctuation of High-end ICT256 Exposure Time

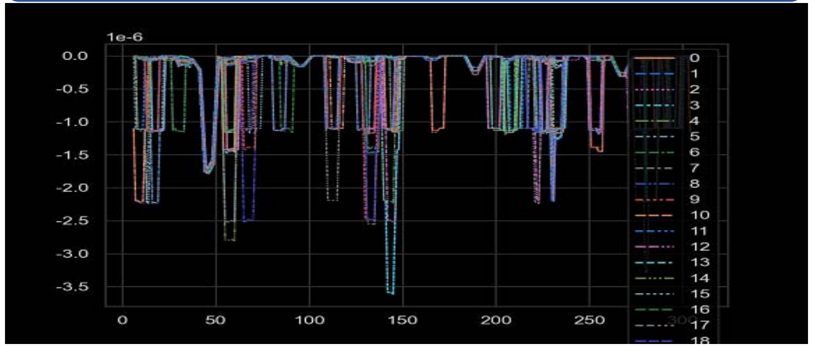


Energy Accumulation [Stack Barrier] Regular Fluctuation of Low-end UCT528 Exposure Time

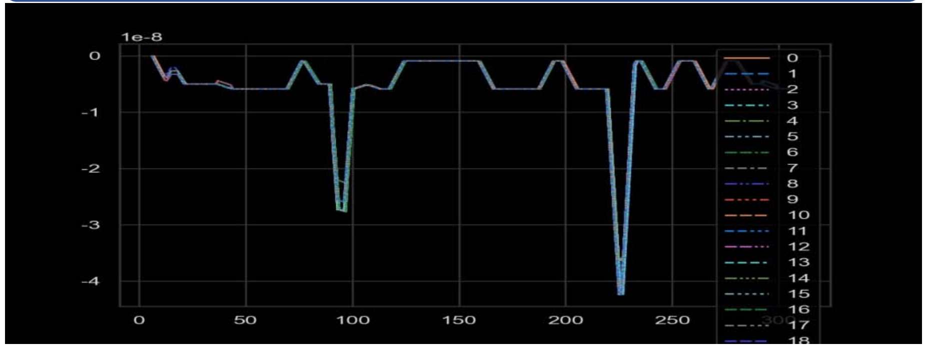

# PyCharm_Web_ AI Big Data Risk Control Product [Brief Introduction]

## AI Risk Control Equipment [V1.0] Version

Code Recording Chip

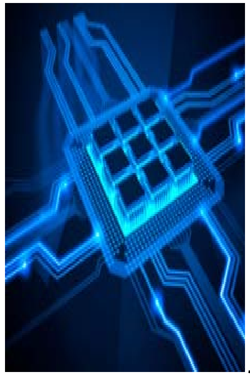

AI Product Shell

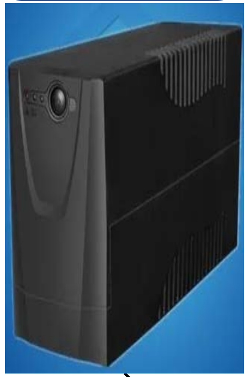

IOT Interface

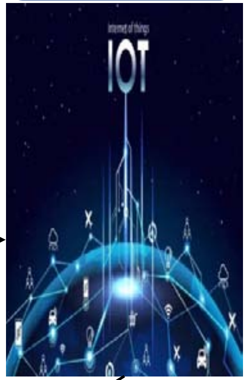


IOT Devices

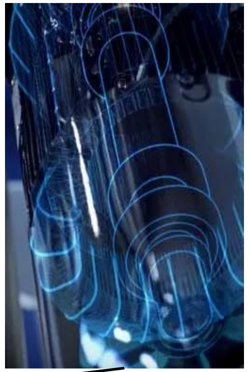

Early Warning System of Edge Lens with Double Core and Heavy Kernel
