# Supplementary material for: Contactless medical equipment AI big data risk control and quasi thinking iterative planning
Source: Sci Rep. 2022 Sep 3;12:15039. doi: 10.1038/s41598-022-18724-5 (PMC9440912; doi:10.1038/s41598-022-18724-5)

Contactless Medical Equipment AI Big Data Risk Control and Quasi Thinking Iterative Planning

Zhu Rongrong, Fudan University, Shanghai, China

rongrongzhu1969@163.com

Manuscript ID: 4b6901f7-42f6-4385-9375-ec6924e102b8

Manuscript Title: Contactless Medical Equipment AI Big Data Risk Control and Quasi Thinking Iterative Planning

1. Core design flow charts of crossing platform language development of "Contactless Medical Equipment AI Big Data Risk Control and Quasi Thinking Iterative Planning" .

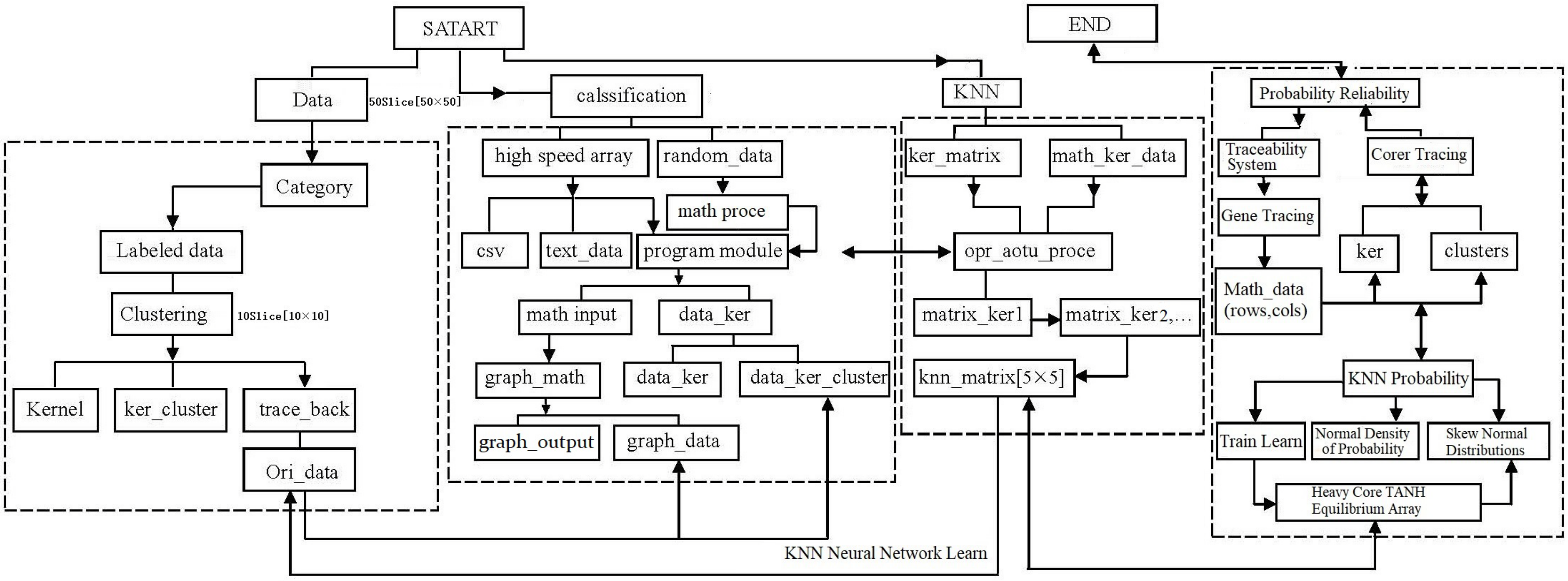

Supplementary Materials-A10.png

2. Dynamically execute the operation, completed the overall data of high-dimensional heavy core clustering big data, and start to generate the predictability and reliability analysis of iCT256,uCT528,DISCOVERY MR750W, as well as local tracing of abnormal with the original data. For example, CT exposure time of iCT256[1.1]=5353ms exceeded the standard, and that of uCT528[1.1]=1500ms exceeded the standard. DISCOVERY MR750W[1.1]=11 SAR RF peaked. The reliability analysis results are iCT256=85.911%,uCT528=69.793%,DISCOVERY MR750W=66.957%.

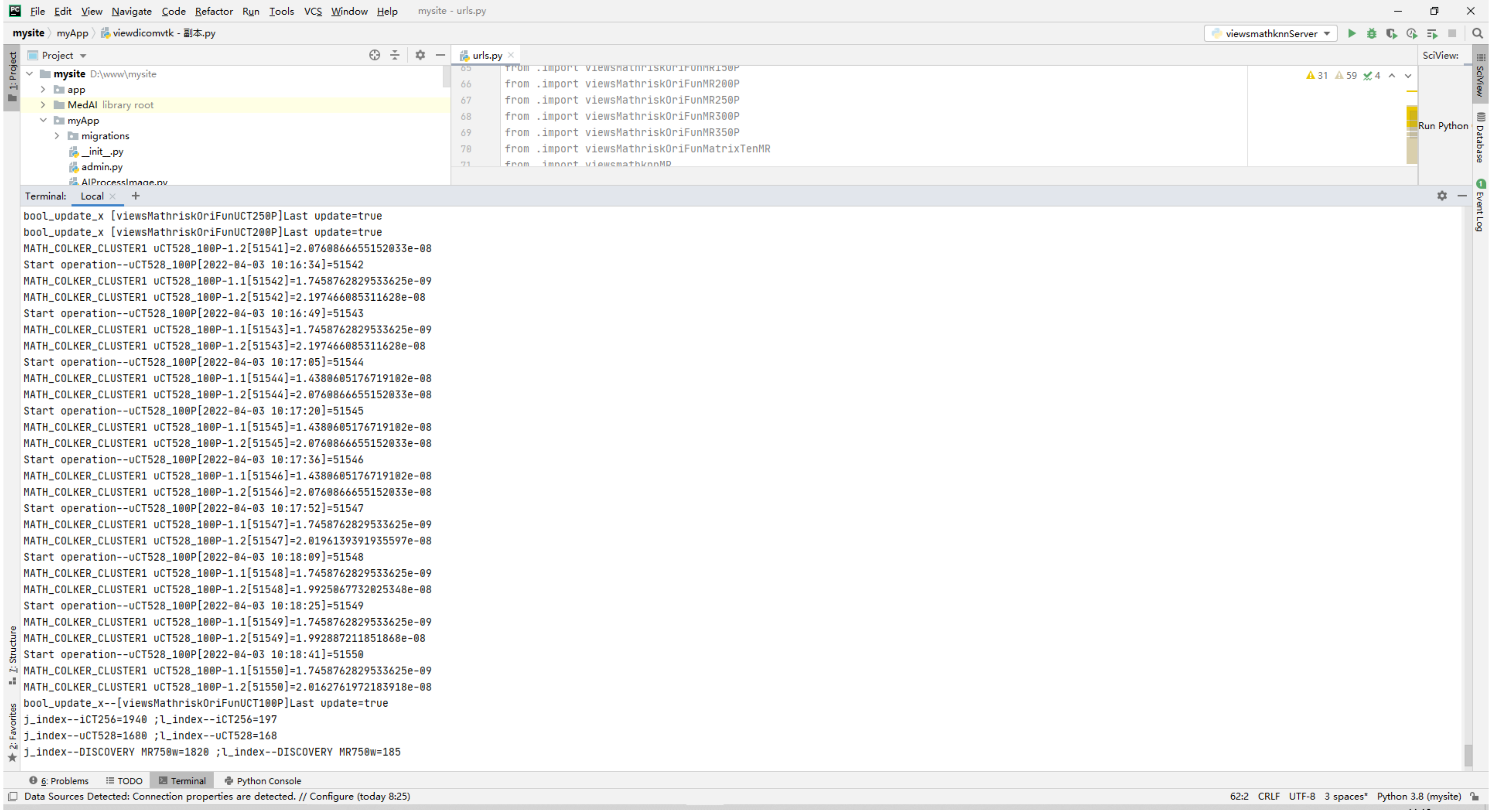

Supplementary Materials-A11.png

i. Generated CT exposure time of iCT256[1.1]=5353ms exceeded the standard, and that of uCT528[1.1]=1500ms exceeded the standard, and Generated DISCOVERY MR750w [1.1]=11 SAR RF peaked. The reliability analysis results are iCT256=85.911%,uCT528=69.793%,DISCOVERY MR750W=66.957%. And iCT256, uCT528, DISCOVERY MR750W dynamic tracking value.

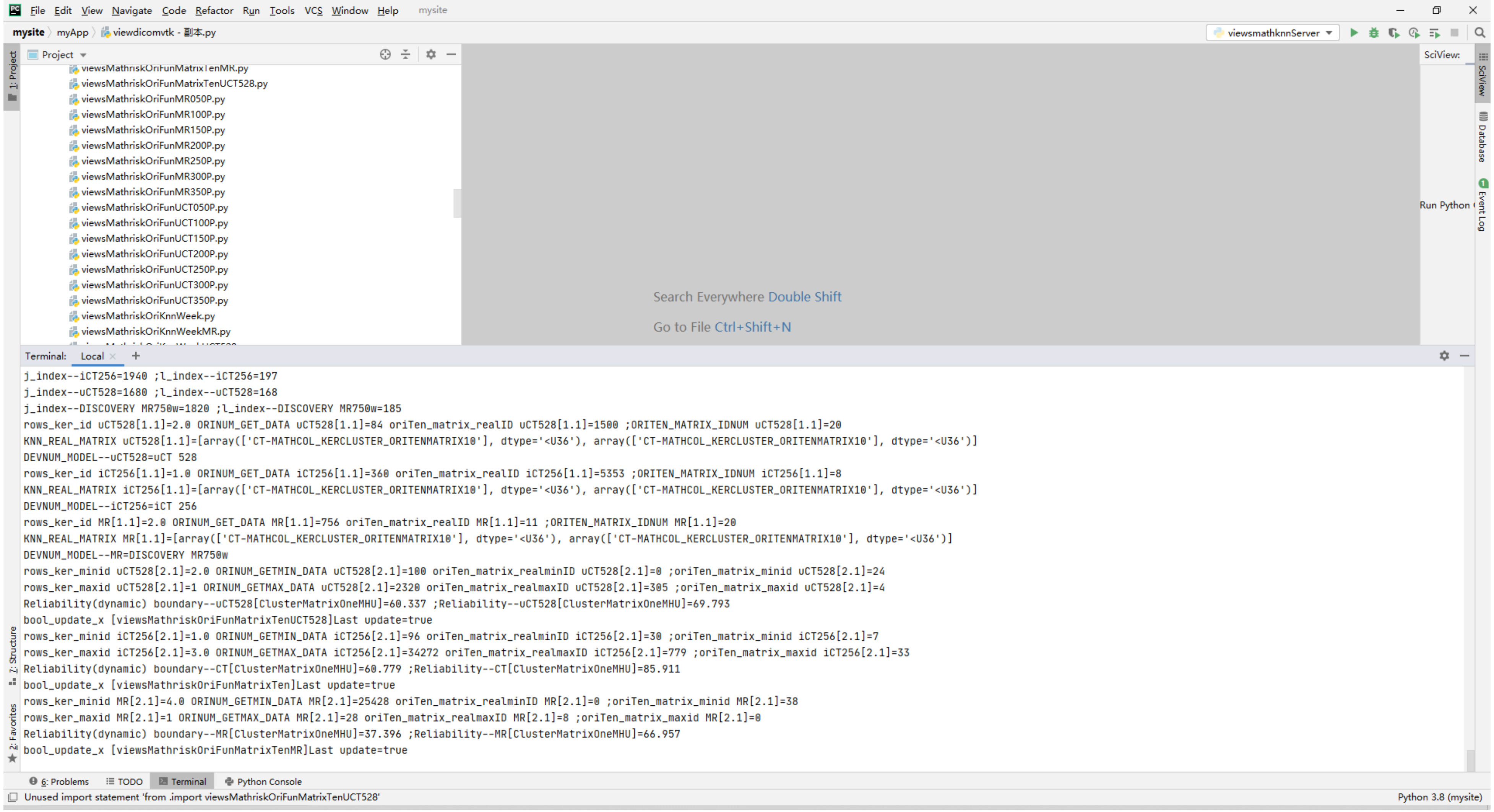

Supplementary Materials-A12.png

3. At the same time, risk control charts of iCT256, uCT528 and DISCOVERY MR750w with heavy core clustering for predictability maintenance are generated.

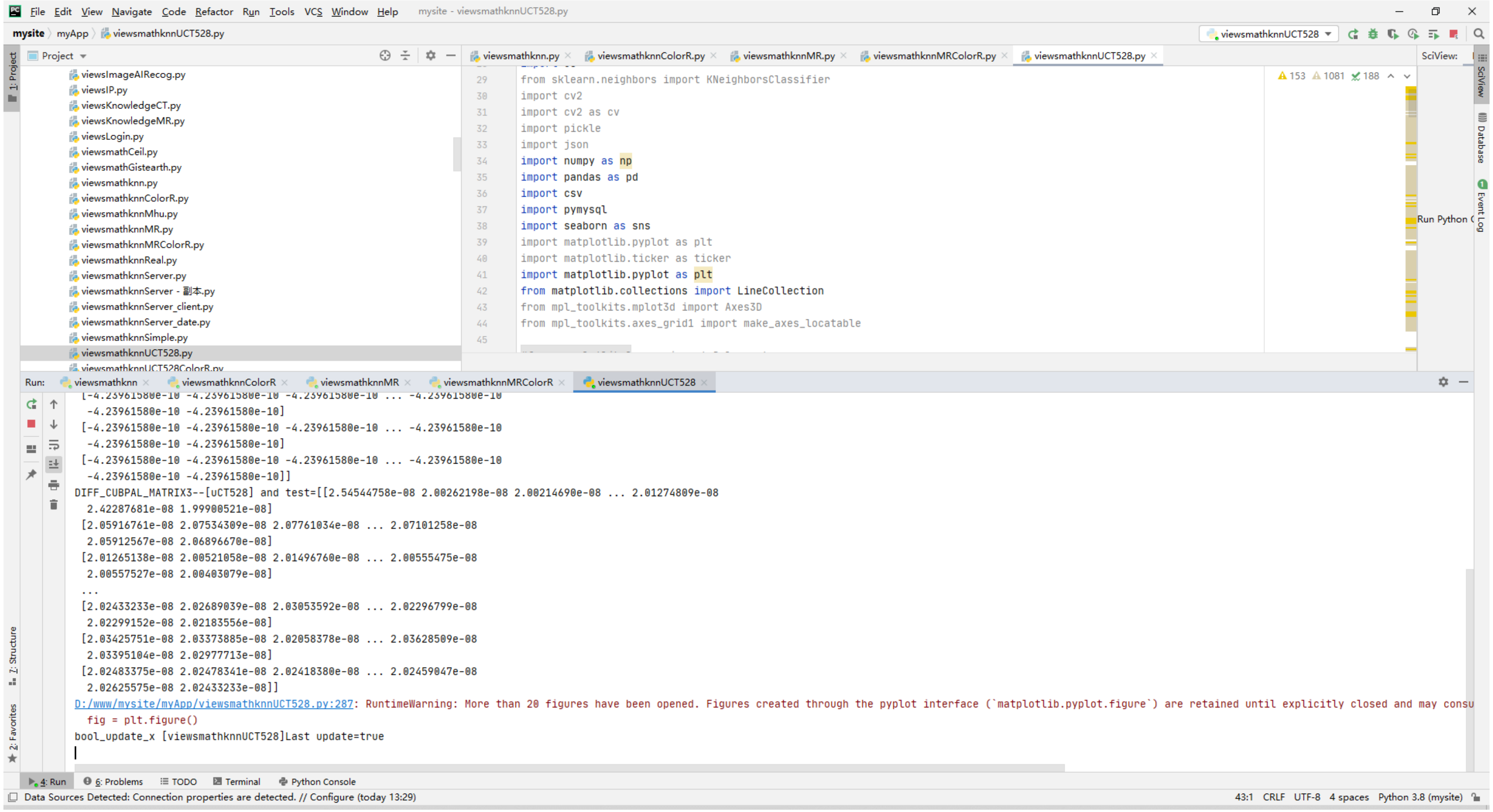

Supplementary Materials-A13.png

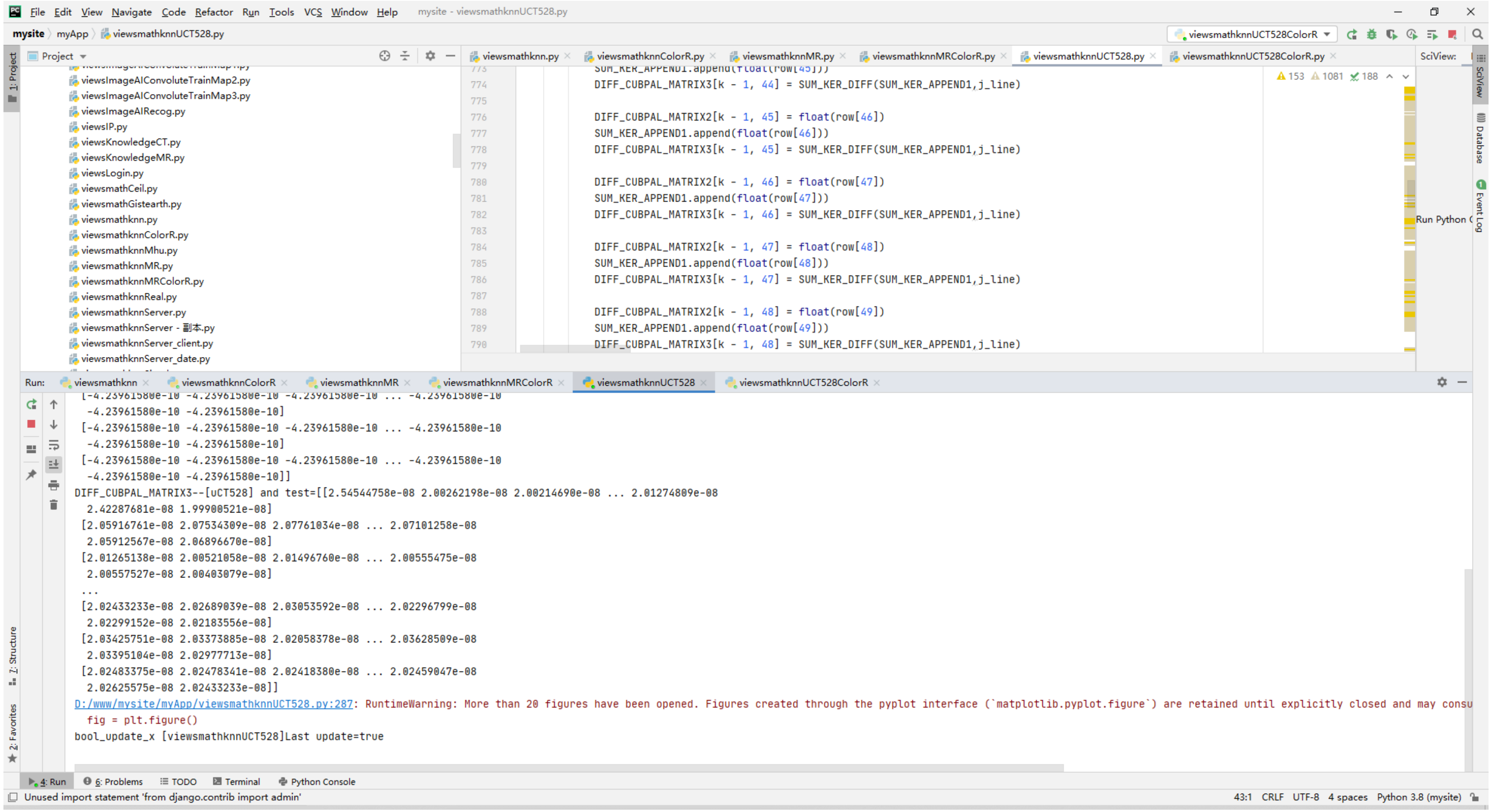

Supplementary Materials-A14.png

4. Heavy core clustering AI big data mathematical model risk controls medical equipment CT/MR clustering lens effected. And differential increment controllable equilibrium weak nonlinear disturbance class function was called local code.

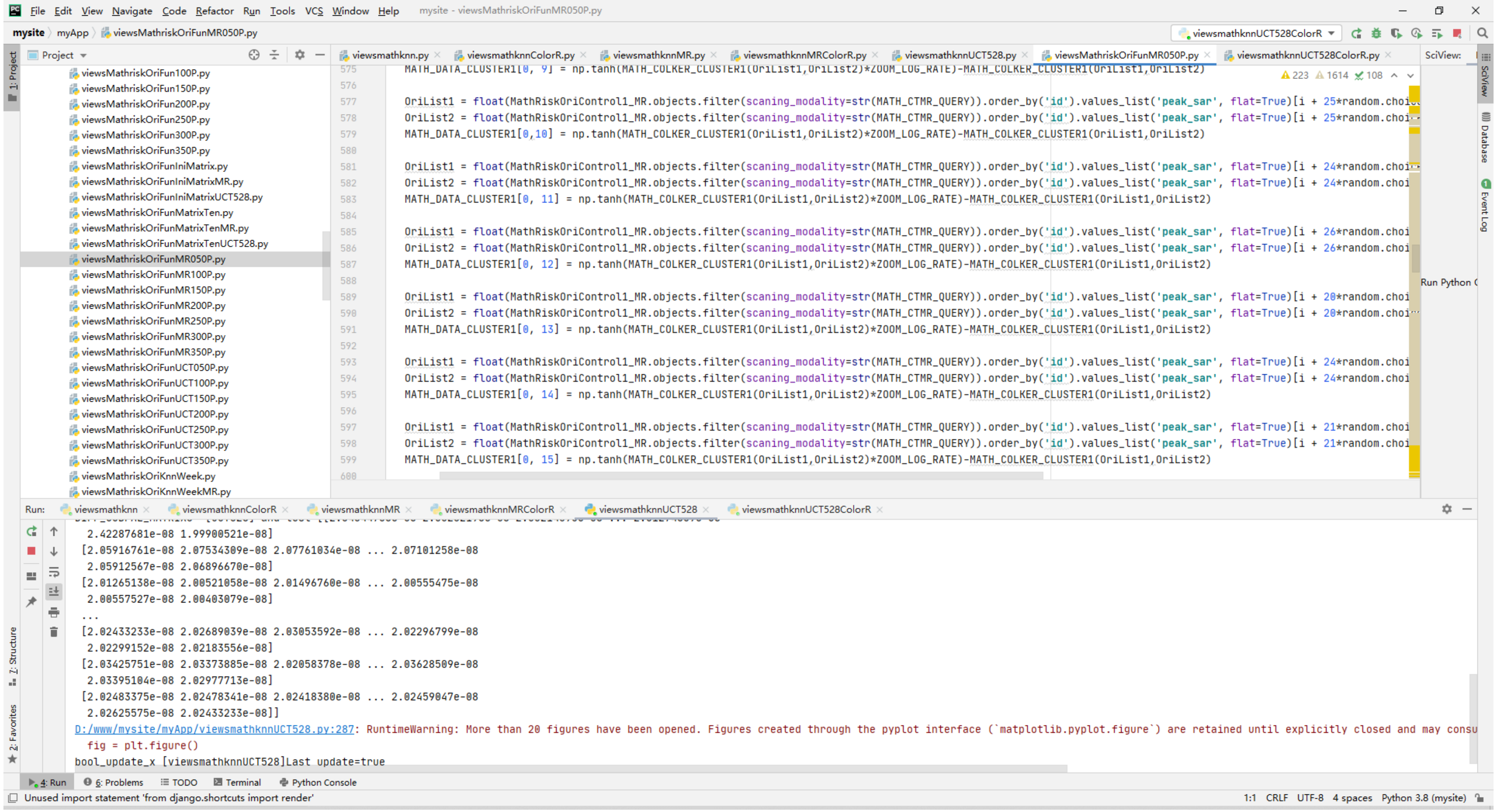

Supplementary Materials-A15.png

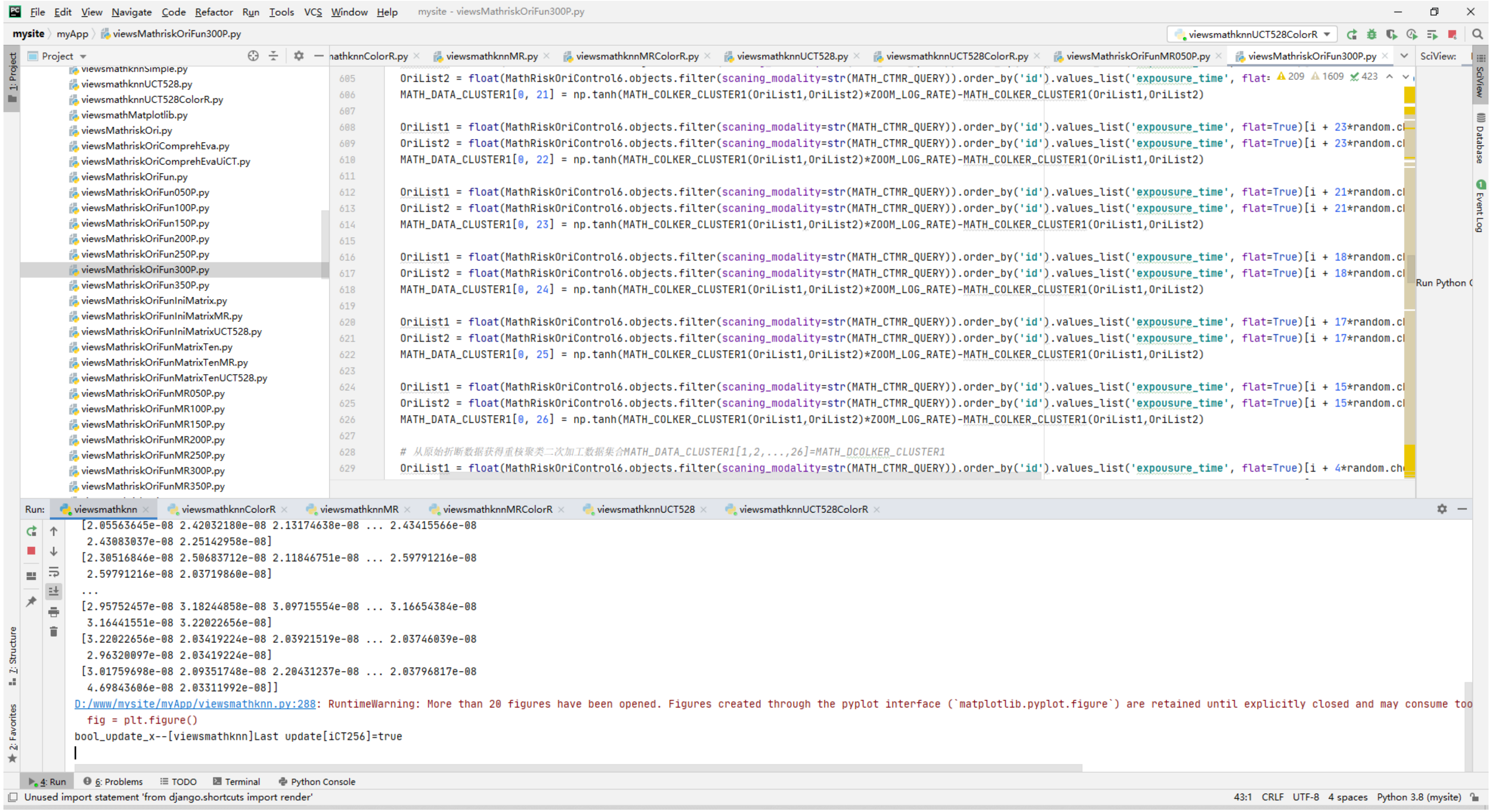

Supplementary Materials-A16.png

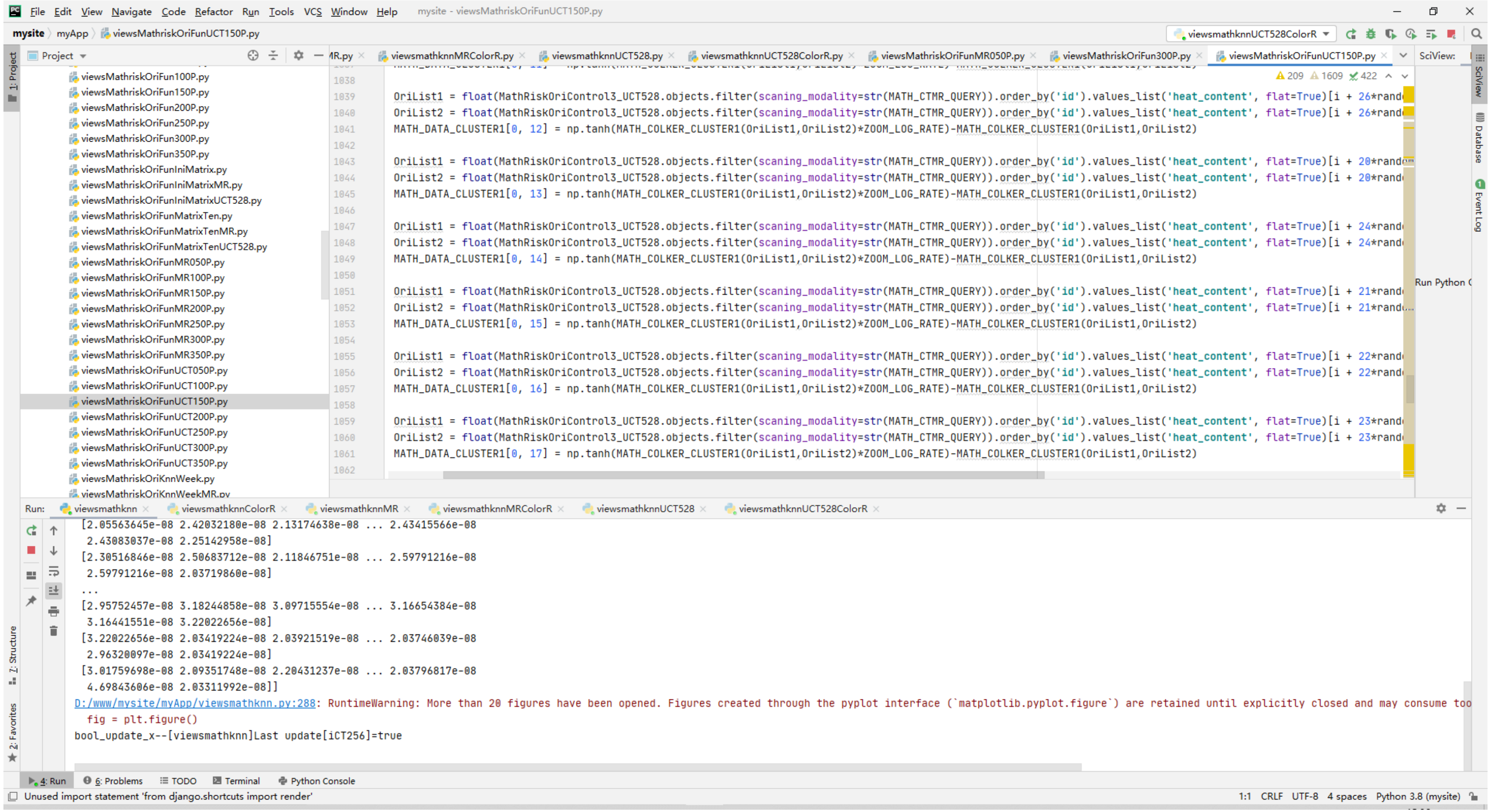

Supplementary Materials-A17.png

5. The high-speed array call of heavy core clustering high-dimensional data group and the technical treatment of high-speed micro vibration of the whole heavy core clustering high-dimensional data group greatly improves the computer running speed and greatly reduce the computer computing resources.

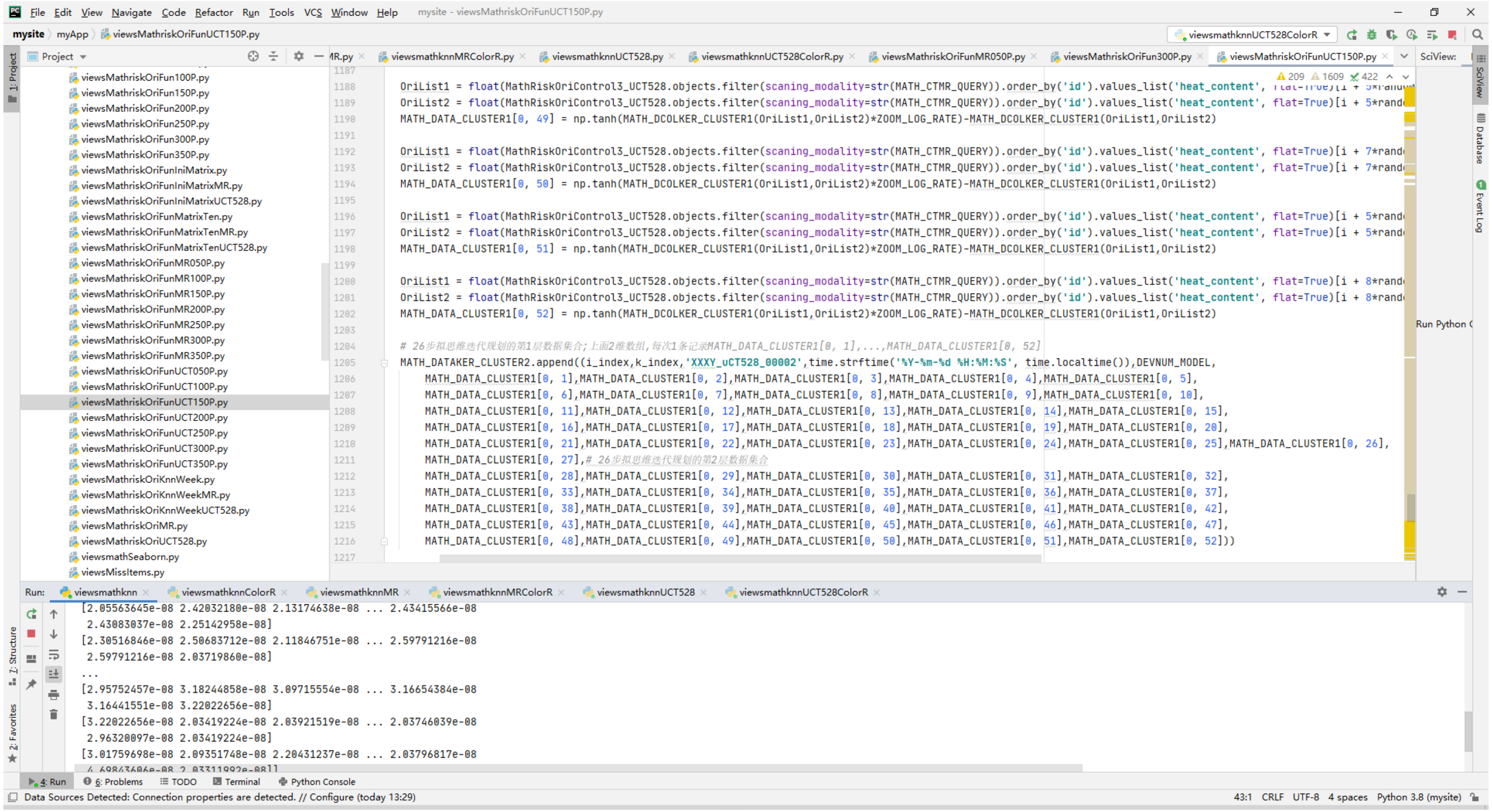

Supplementary Materials-B01.png

6. **Research Plan:** Contactless Medical Equipment AI Big Data Risk Control and Quasi Thinking Iterative Planning. **ResearchObjectives:** Visual predictability based on weakly nonlinear morphology maintains stable model structure. Form fully automatic and intelligent collection, classification, analysis and early warning of internal information and data of medical equipment, and display it through the front end of the web.

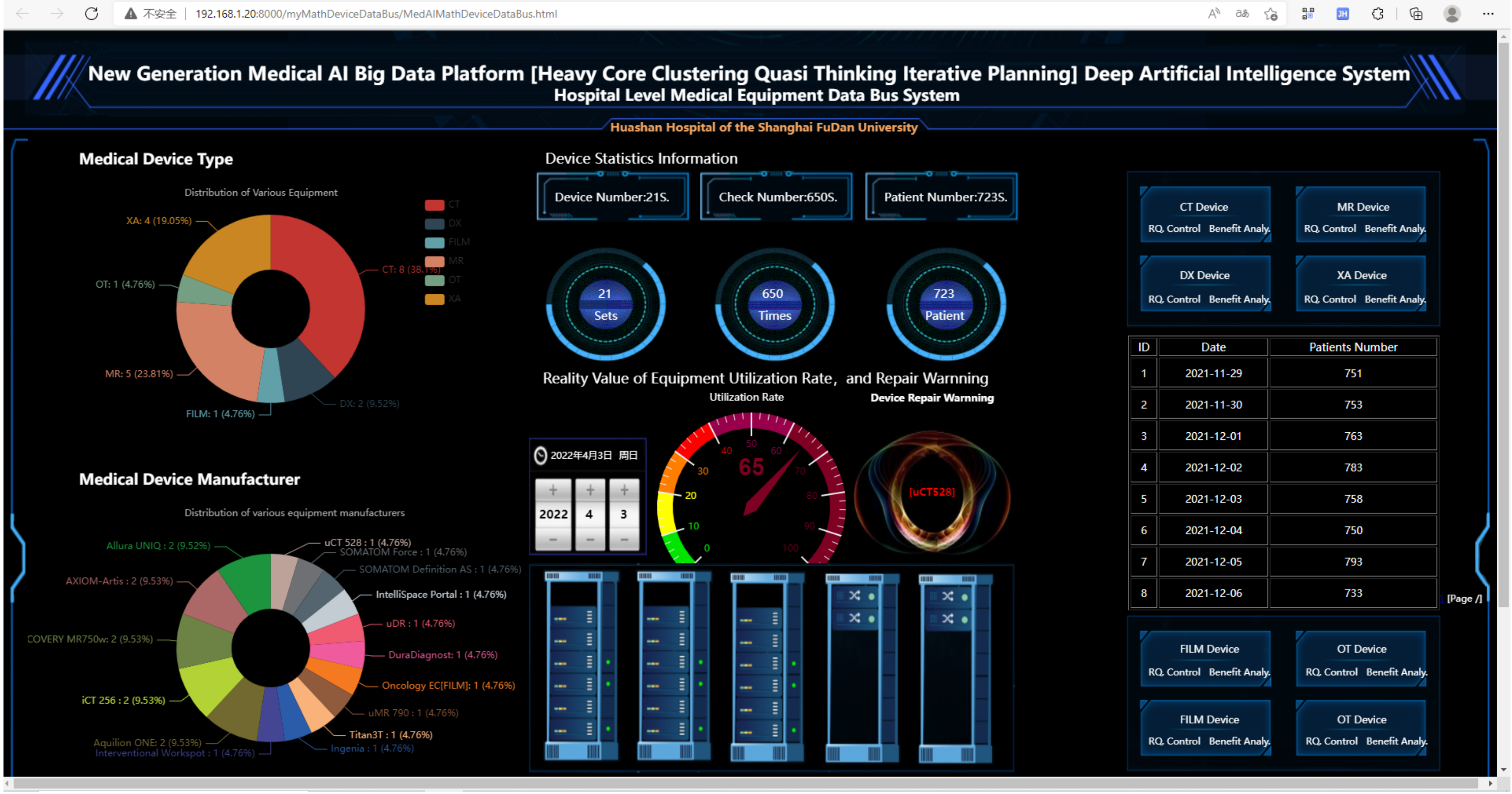

Supplementary Materials-B02.png

7. Predictive maintenance of high-end iCT256 and NMR DISCOVERY MR750w,and nature of iCT256 exposure time and heat capacity(mhu%) polar high-dimensional map and DISCOVERY MR750w RF peak SAR and image definition polar coordinate high-dimensional map. The combined parameters (formulas) of iCT256 ball tube, DISCOVERY MR750w RF, mT magnetic field, etc. form the reliability, reliable boundary, dynamic tracking value and mean value of the whole life cycle comprehensive evaluation of the machine. The comprehensive evaluation reliability is ct_tube_life1. Reliability boundary ct_tube_life2. Dynamic tracking value ct_tube_life3, mean ct_tube_life4. Core curve: comprehensive evaluation reliability is ct_tube_life1 curve and dynamic tracking value ct_tube_life3, when ct_tube_life3 curve crosses reliability boundary ct_tube_life2 curve, the device will change in the next few days.

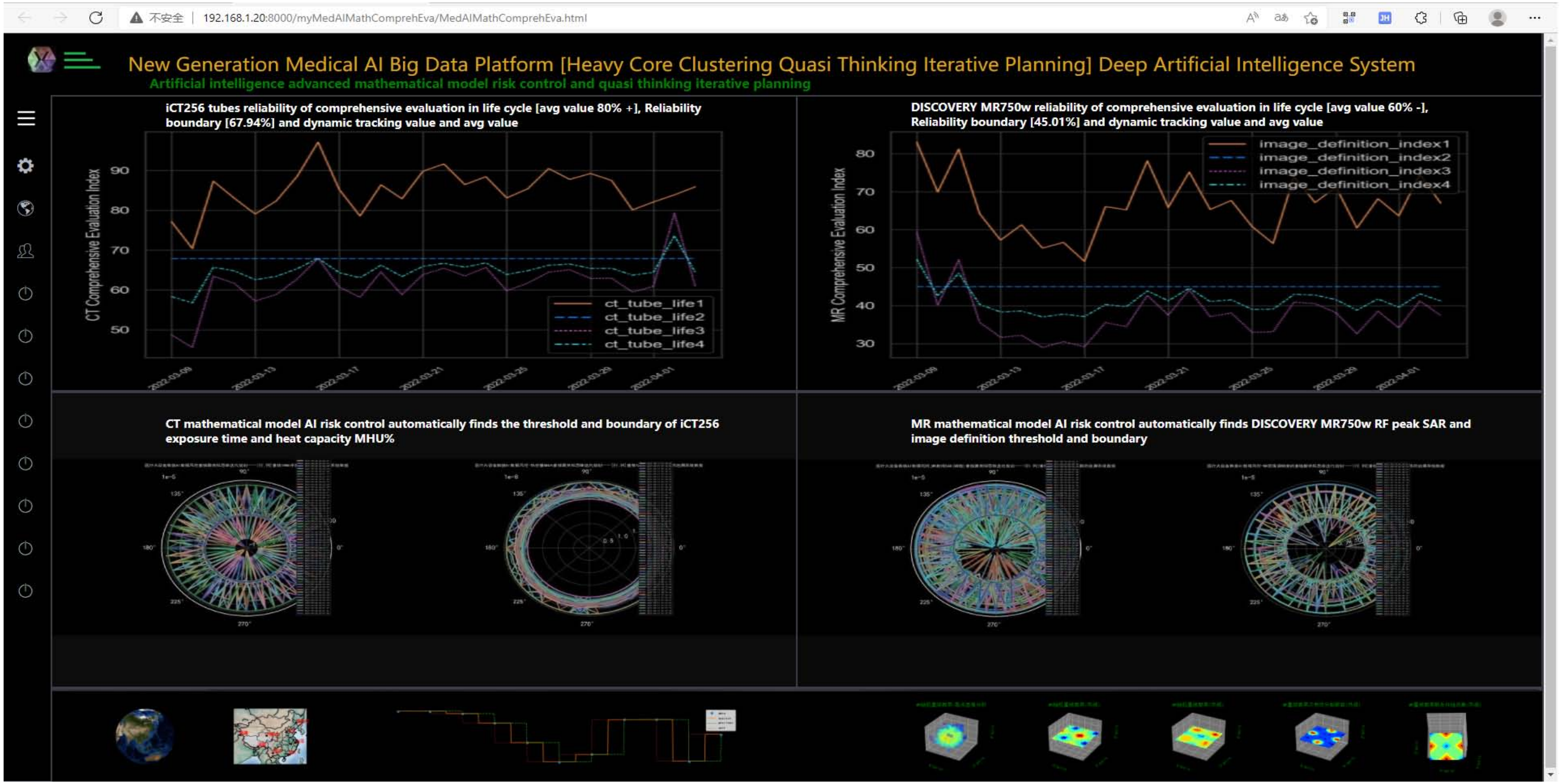

Supplementary Materials-B03.png

8. Contactless automation and intelligent collection of machine parameters of high-end iCT256 large medical equipment and big data AI digital analog risk control. Form the distribution curve of various machine parameters, and finally build dual core heavy core clustering of various lens risk control data with deep statistical AI mathematical model, which greatly improves the predictability, intelligent management and intelligent scheduling analysis of equipment in the hospital. At the same time, the evaluation of whether the machine abnormal change causes harm to the human body is constructed.

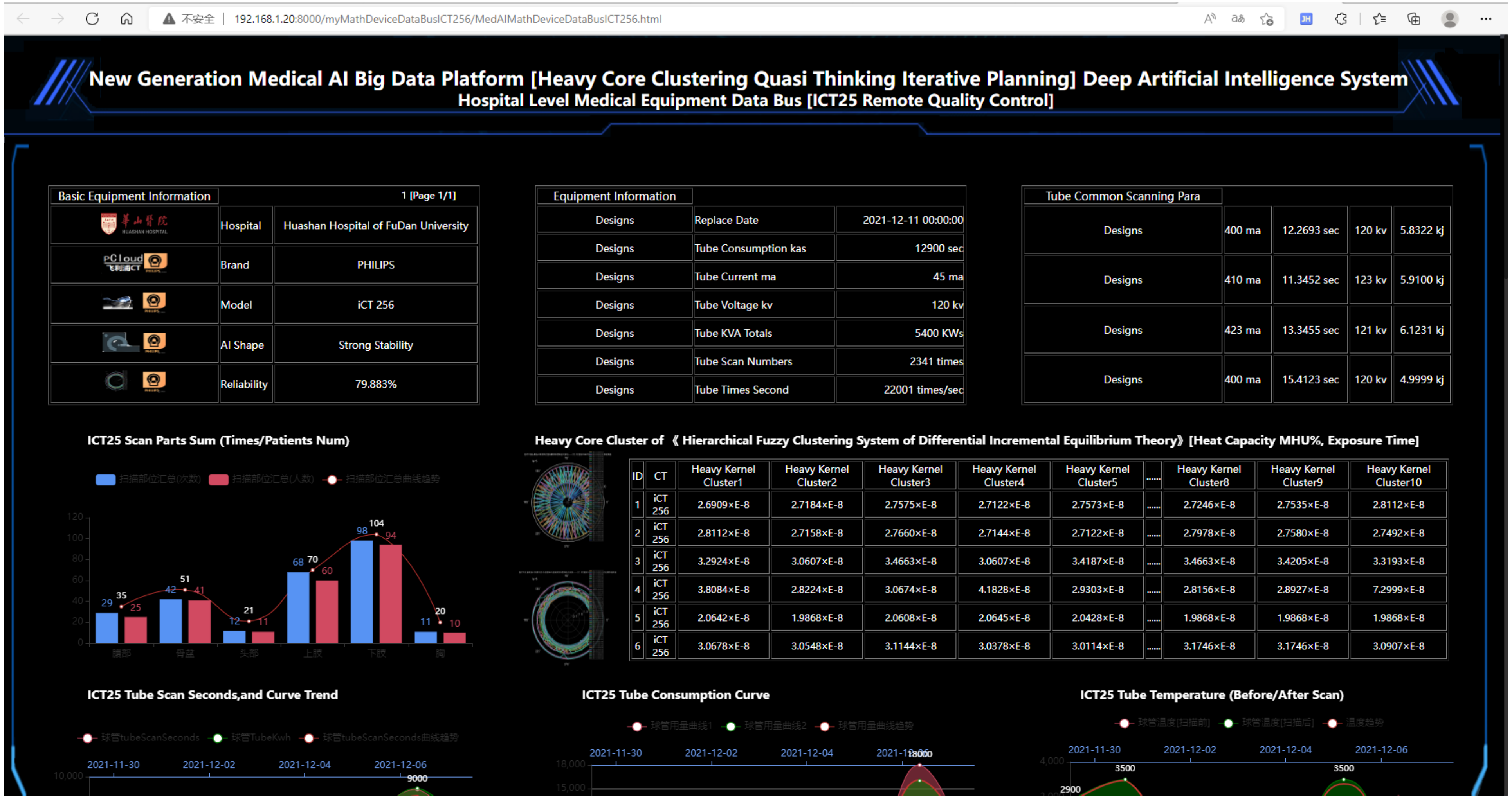

Supplementary Materials-B04.png

9. High-end iCT256's artificial intelligence advanced mathematical model risk control and quasi thinking iterative planning. AI risk control CT exposure time, heavy core clustering polar graph of heat capacity mhu%, tanh balanced high-dimensional traceability system, depth statistical morphological regression fitting graph and density estimation graph.

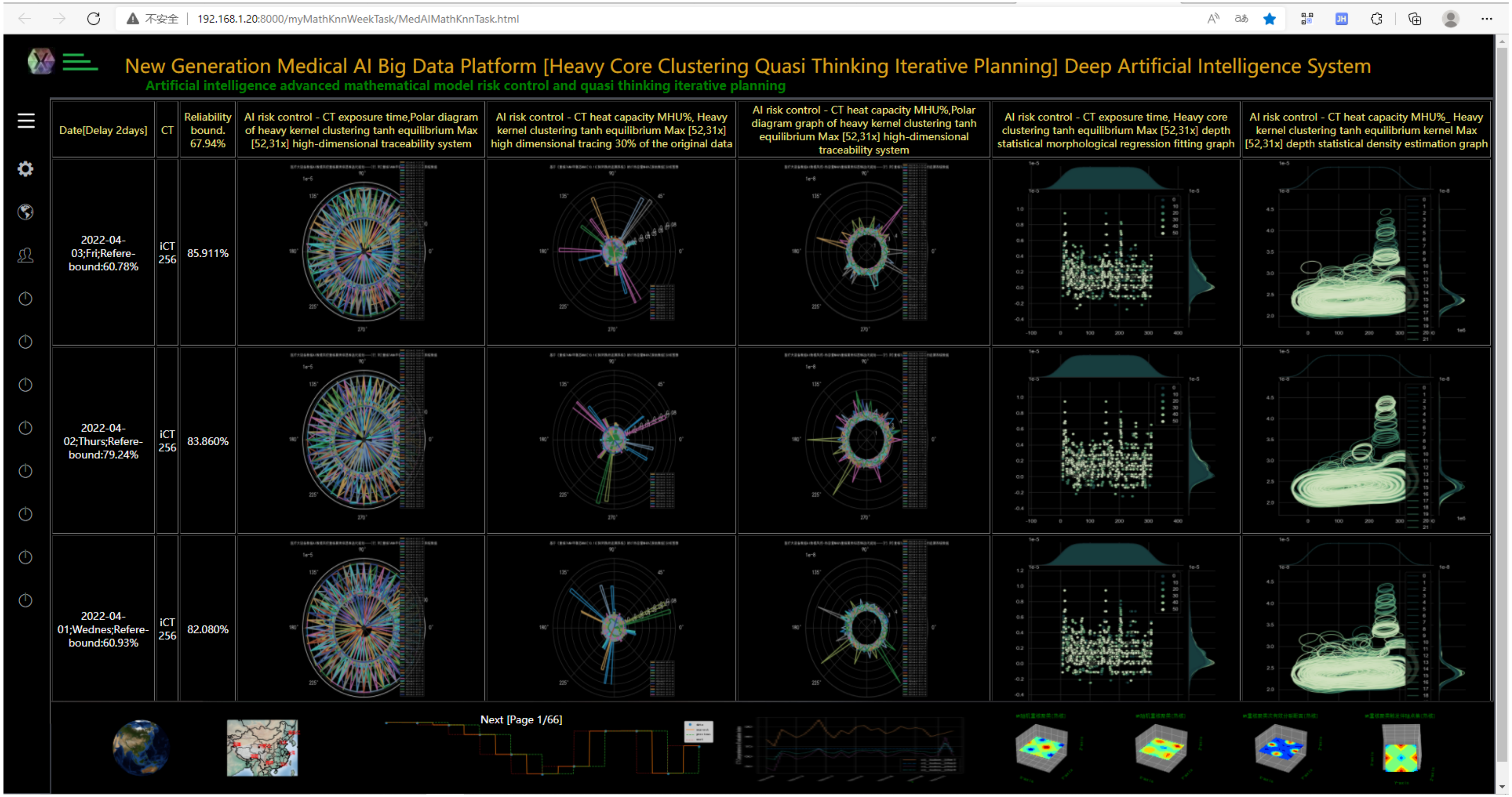

iCT256_2021-08-29.png

i. AI depth statistical mathematical model risk control CT exposure time, heat capacity mhu% heavy core clustering polar graph is more perfect, scientific graph tanh balanced high-dimensional traceability system, and deep statistical morphological regression fitting and density estimation graph reliable structure.

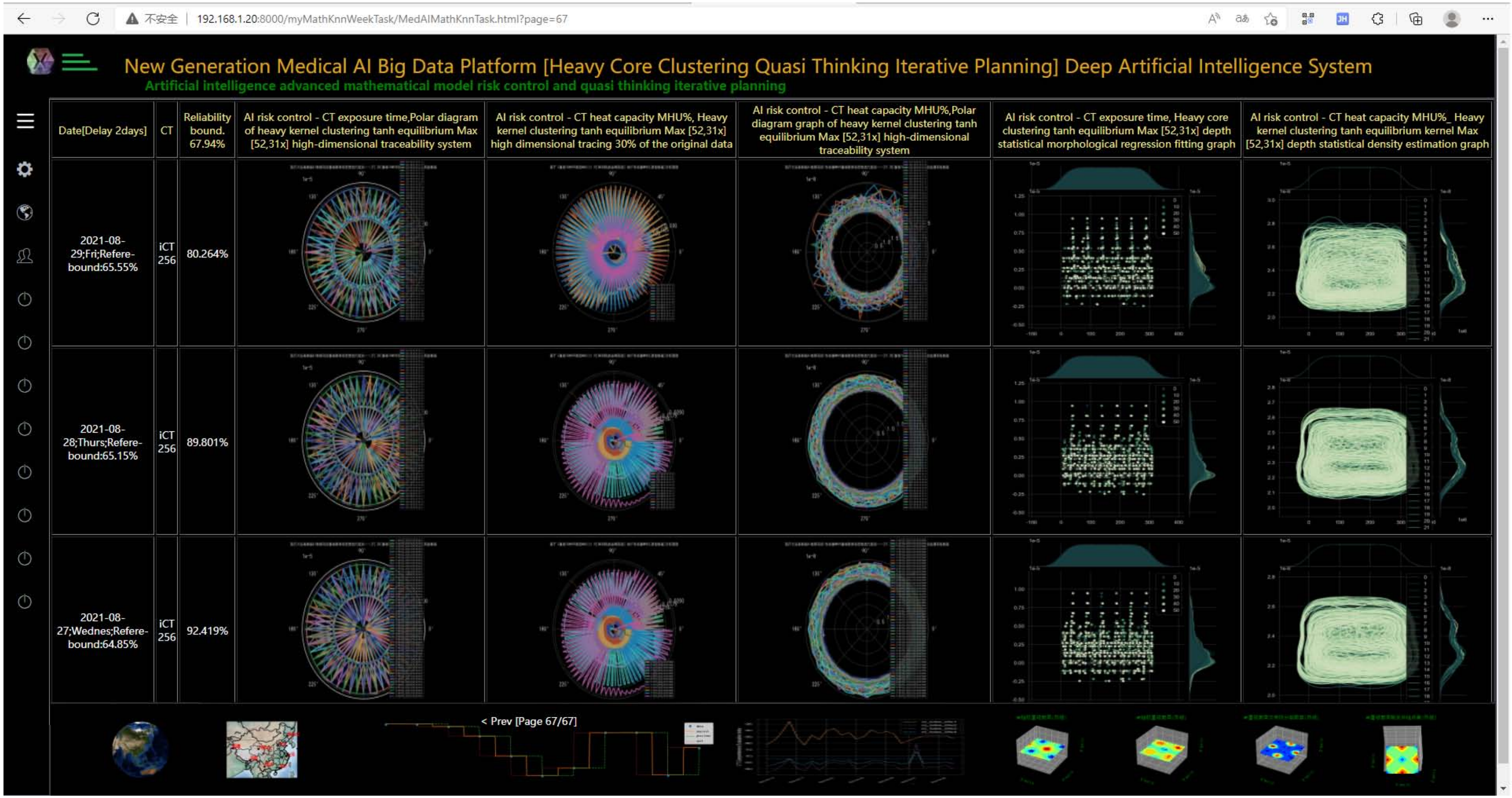

Supplementary Materials-B05.png

ii. AI depth statistical mathematical model risk control is directly related to the predictability of iCT256 equipment, which is reported for repair and early warning 4-7 days in advance. The **stability and reliability of the dual core heavy core lens matrix[10,1] cluster scale morphology** of the internal information comprehensive index of the CT tubes indicates that the performance of the iCT256 is stable.

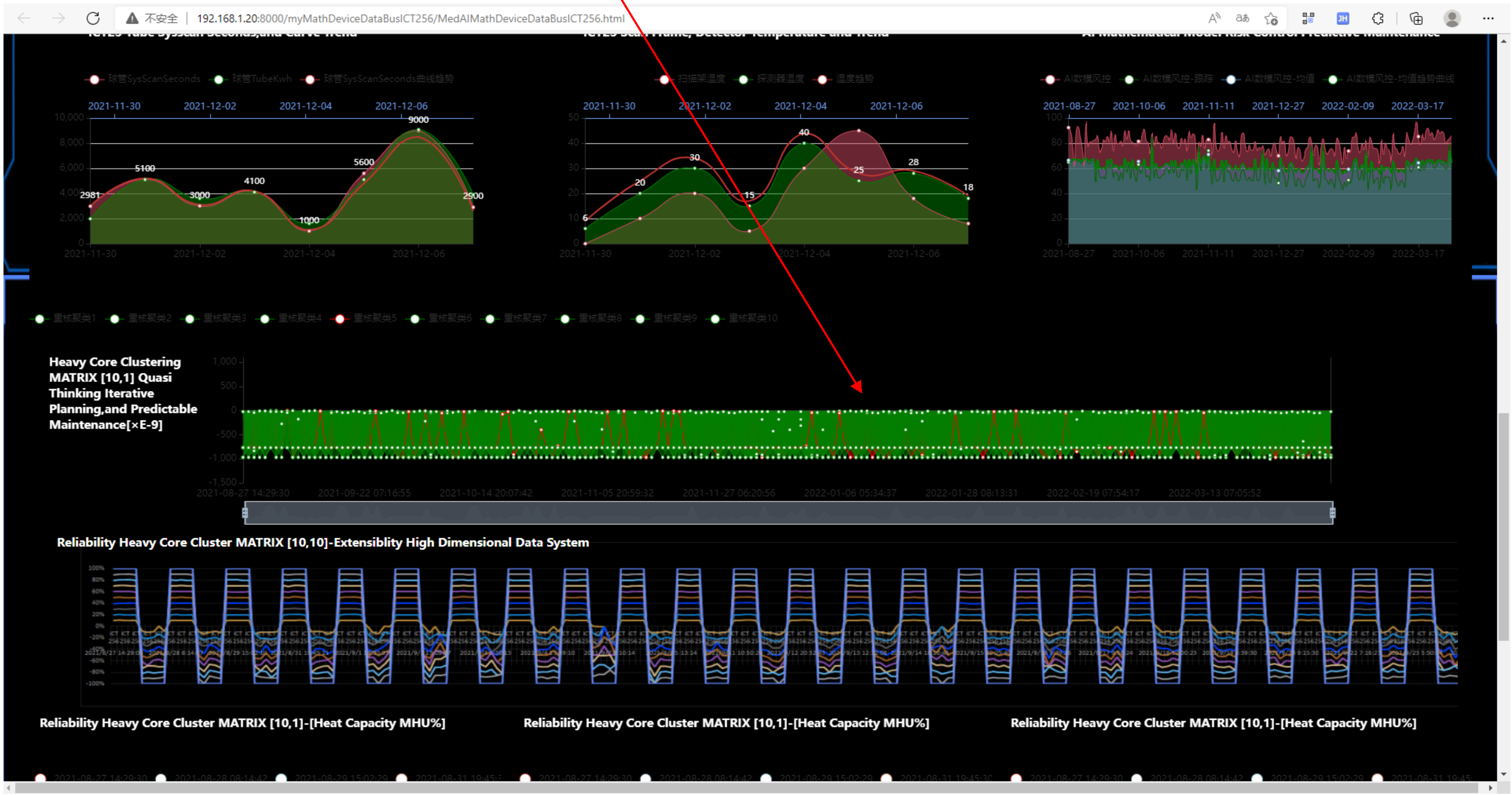

Supplementary Materials-B06.png

10. Contactless automation and intelligent collection of machine parameters of high-end DISCOVERY MR750w large medical equipment and AI mathematical model risk control of big data; Form the distribution curve of various machine parameters, and finally build a dual core heavy core cluster of various lens risk control data with deep statistical AI mathematical model, which can analyze the clarity index map of NMR image, and greatly improve the predictability, intelligent management and intelligent scheduling analysis of equipment in the hospital. At the same time, the evaluation of whether the machine abnormal change causes damage to the human body is constructed.

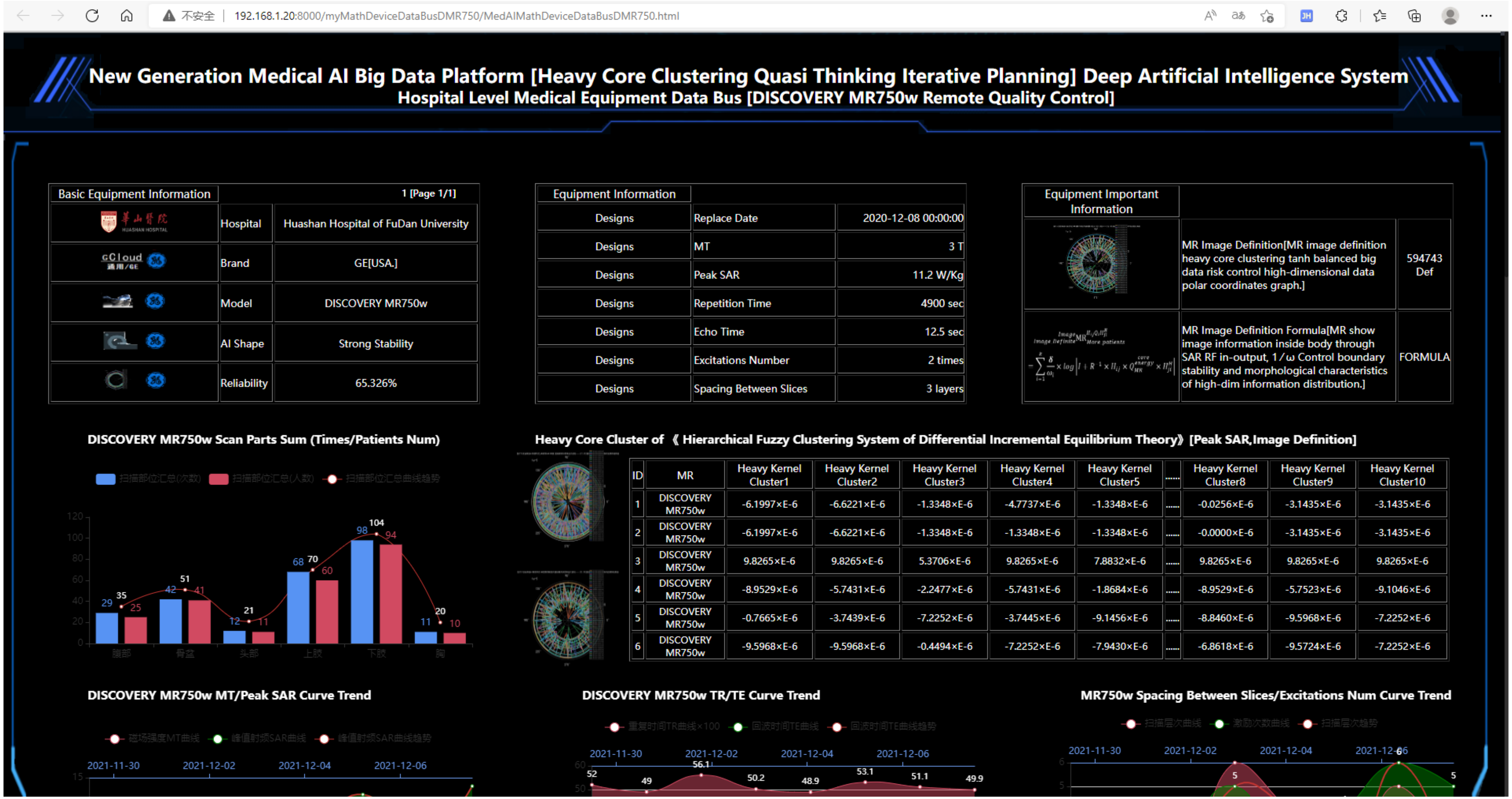

Supplementary Materials-B07.png

11. DISCOVERY MR750w artificial intelligence advanced mathematical model risk control and quasi thinking iterative planning. AI risk control MR machine internal information SAR peak and its fluctuation data heavy kernel clustering polar coordinate atlas is more perfect and scientific figure tanh balanced high-dimensional traceability system, and the reliability structure of depth statistical morphological regression fitting and density estimation diagram.

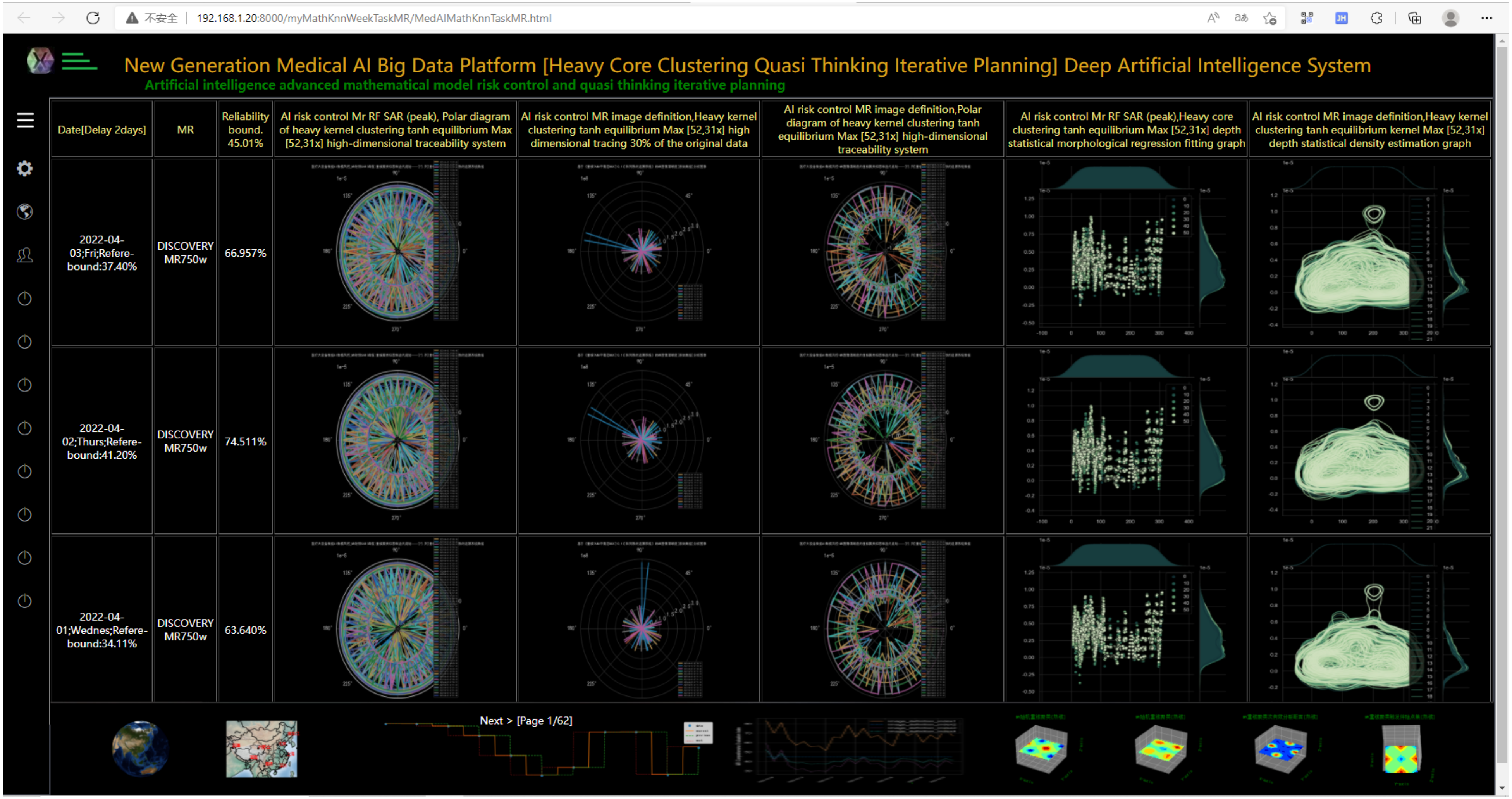

DISCOVERY MR750w_2021-10-06.png

i. AI risk control of MR SAR peak and NMR pause, as well as more perfect and scientific polar graph of heavy kernel clustering of fluctuation data, tanh balanced high-dimensional traceability system. Moreover, the polar graph tanh equilibrium graph of the big data depth statistical heavy kernel clustering mathematical model of the image definition of the MR machine's internal information parameter group (MT, SAR, TR, TE, etc.) and the reliability structure of the depth statistical morphological regression fitting and density estimation graph.

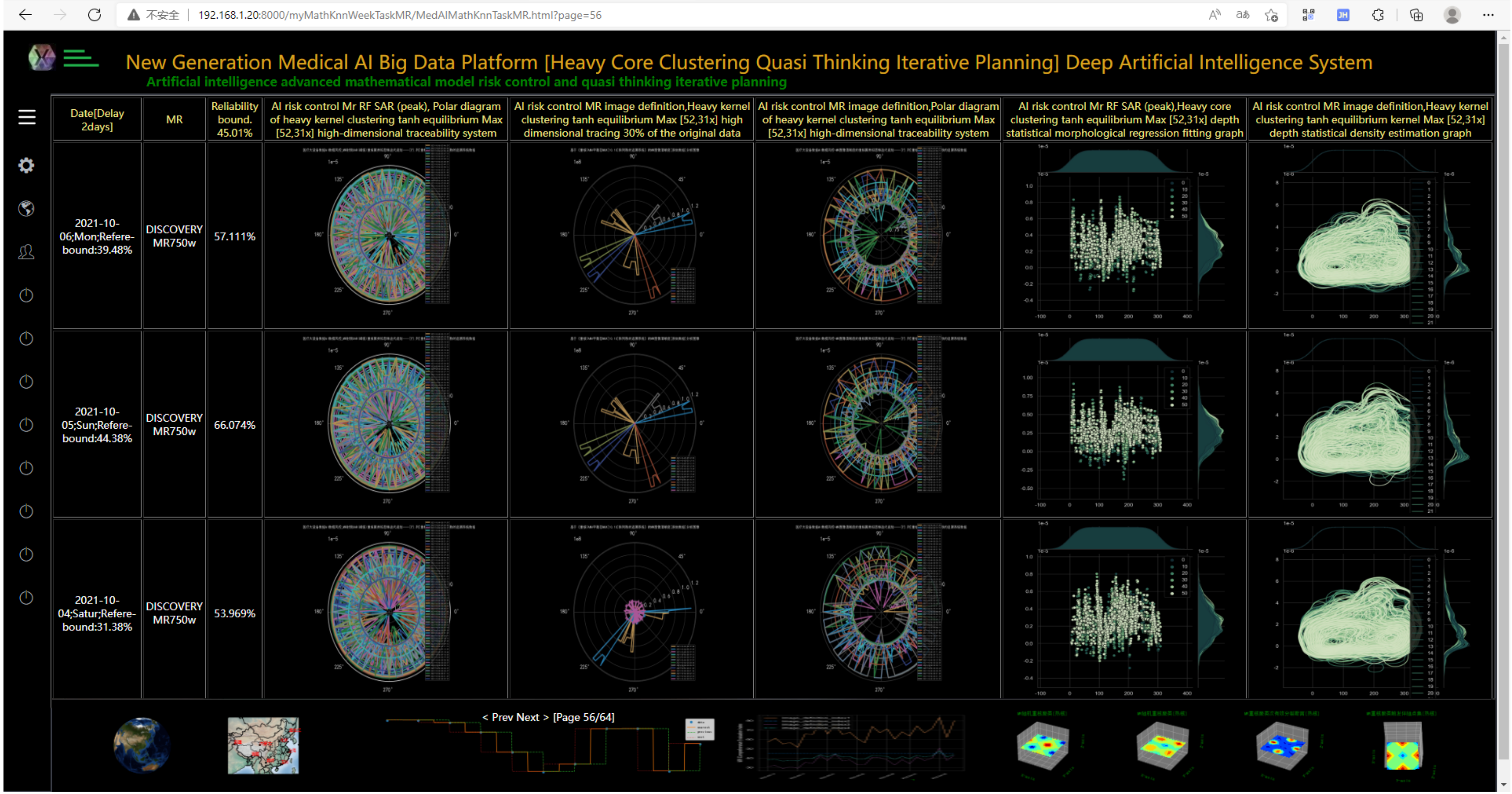

Supplementary Materials-B08.png

ii. AI depth statistical mathematical model risk control is directly related to the predictability of DISCOVERY MR750w equipment to apply for repair and early warning 4-7 days in advance. The **stability and reliability of the scale form of the dual core heavy core clustering lens (matrix[10,1])** of the internal comprehensive information index of DISCOVERY MR750w machine indicate that the performance of its equipment is stable.

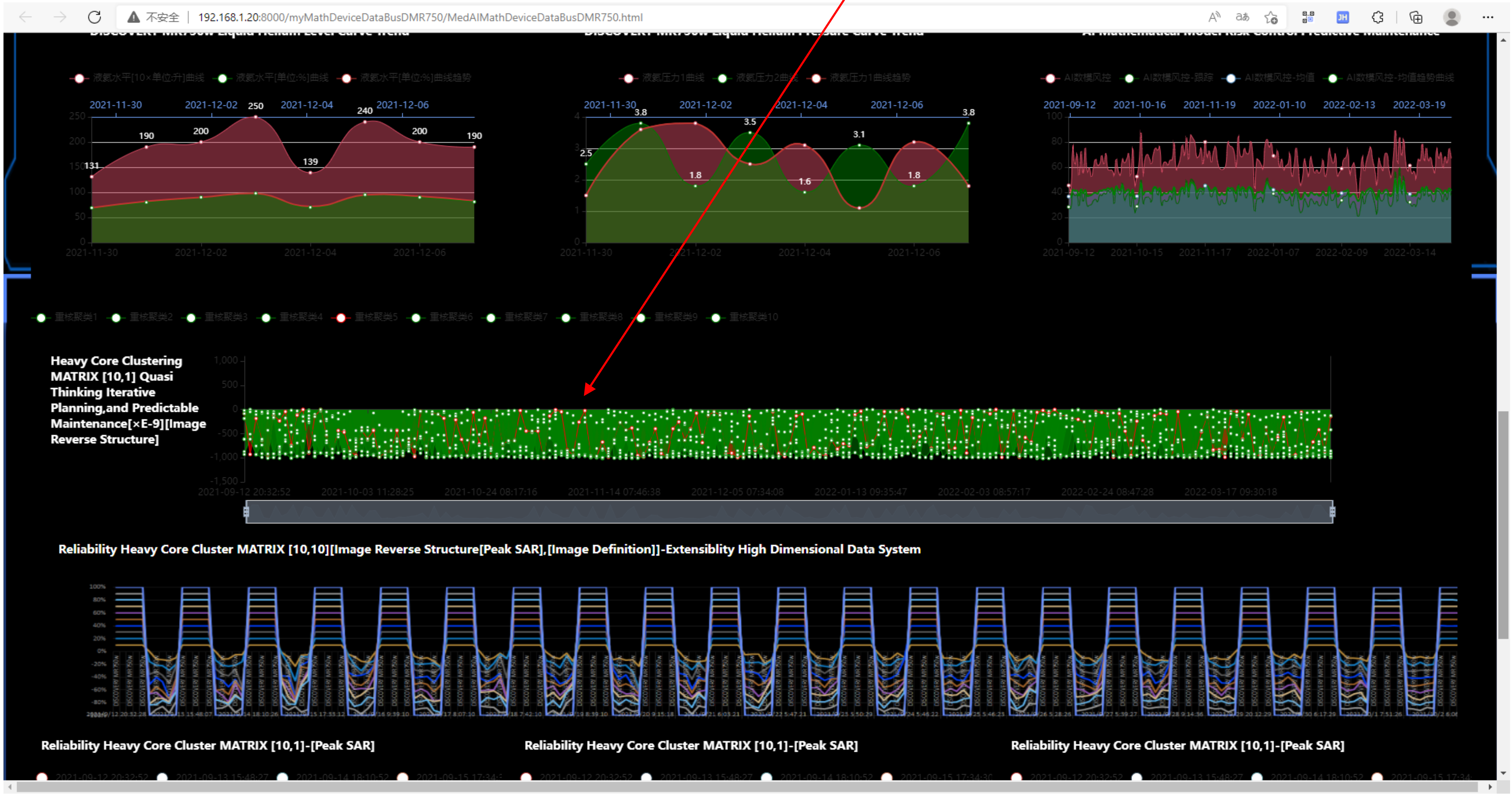

Supplementary Materials-B09.png

12. Contactless automation and intelligent collection of machine parameters of low-end uCT528 medical equipment and AI mathematical model risk control of big data; Form the distribution curve of various machine parameters, and finally build dual core heavy core clustering of various lens risk control data with deep statistical AI mathematical model, which greatly improves the predictability, intelligent management and intelligent scheduling analysis of equipment in the hospital. At the same time, the evaluation of whether the machine abnormal change causes harm to the human body is constructed.

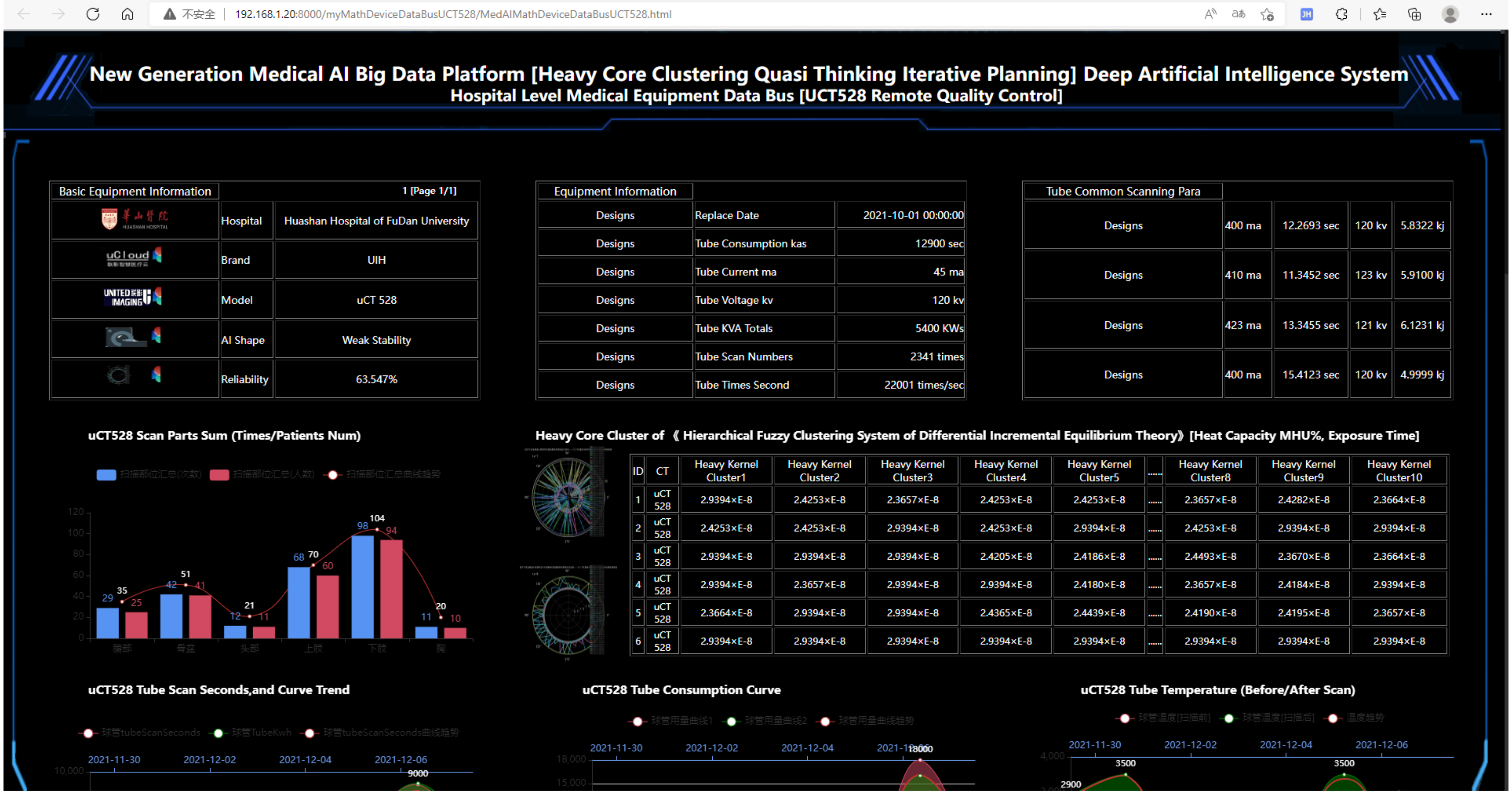

Supplementary Materials-B10.png

13. Low-end uct528's artificial intelligence advanced mathematical model risk control and quasi thinking iterative planning. AI mathematical model risk control CT exposure time, heat capacity(mhu%) heavy core clustering polar graph tanh balance high-dimensional traceability system, the formed high-dimensional polar graph is less scientific, stable and aesthetic than the high-end iCT256, and forms deep statistical morphological regression fitting graph and density estimation graph.

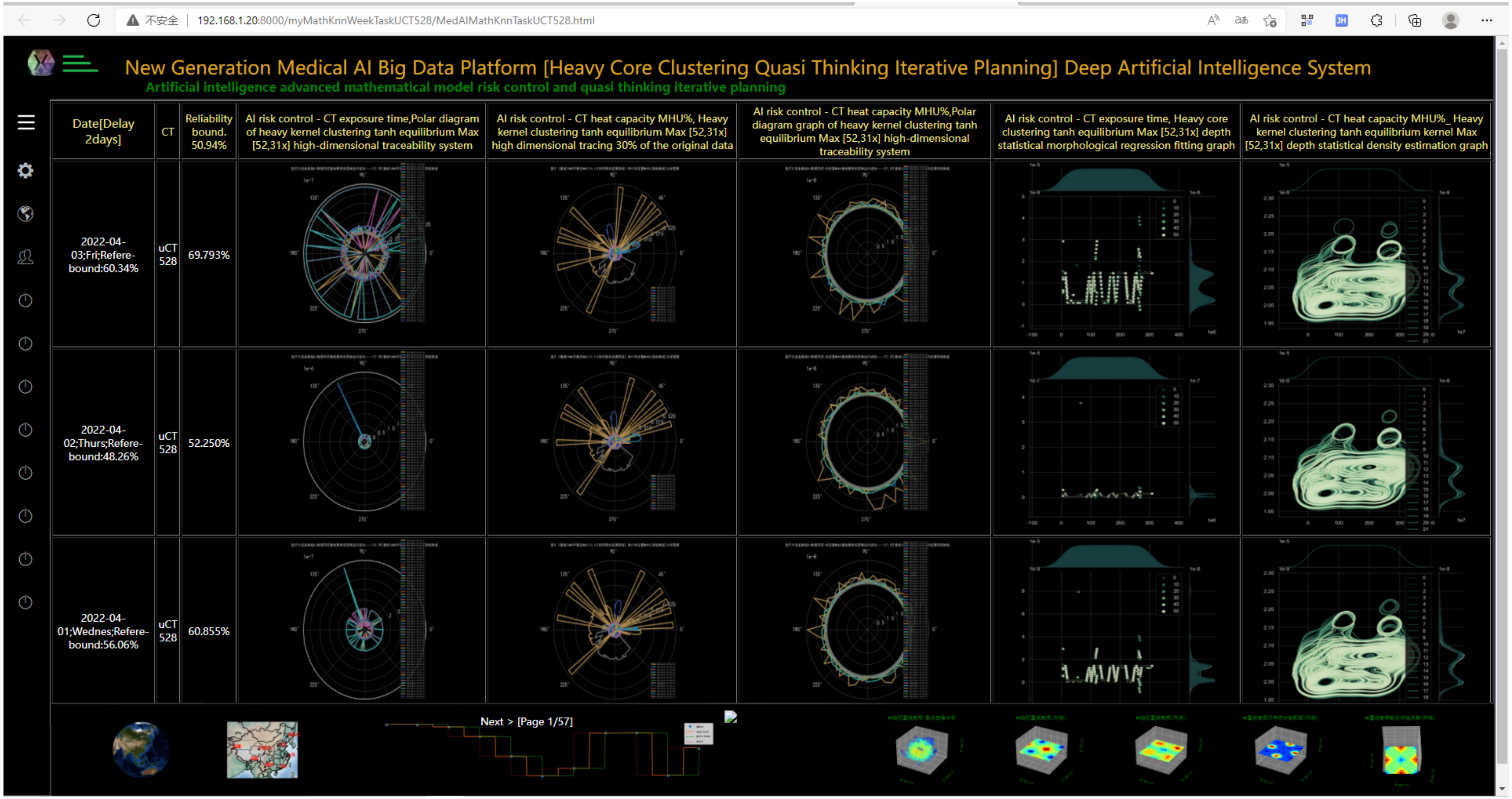

uCT528_2021-10-03.png

i. AI depth statistical mathematical model risk control of the low-end uCT528 dual core heavy core clustering polar coordinate map of CT exposure time and heat capacity(mhu%) is approaching the high-end CT, repairing the complex dependency parameter group of low-end to high-end CT equipment through the energy fluctuation of the dual core heavy core edge lens, and forming a visual low-end equipment how to obtain the mixed reverse complex dependency group parameters of high-end equipment.

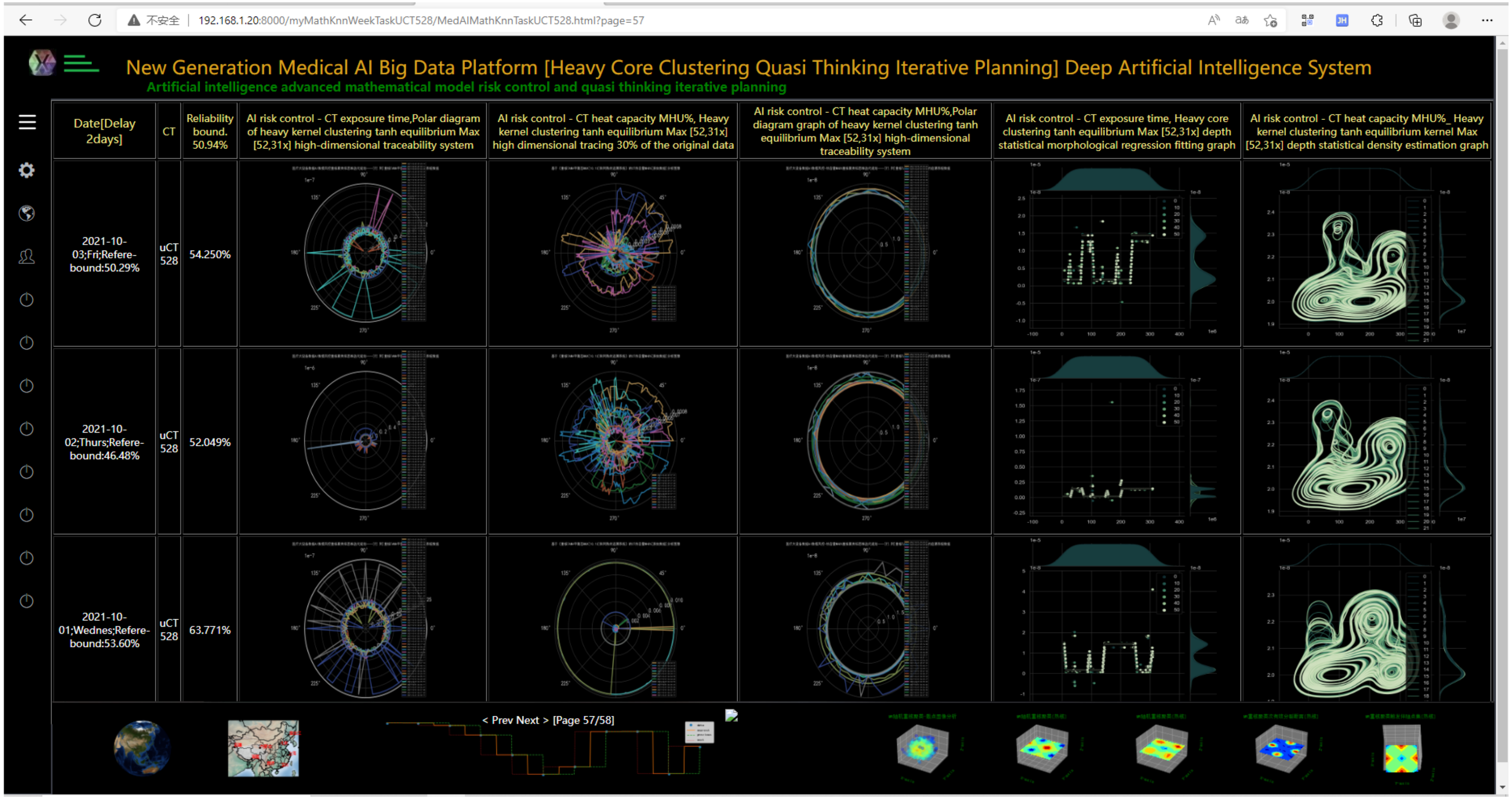

uCT528_2021-10-27.png

ii. The high-dimensional information field exposed by the low-end uCT528 is still in an unstable stage. At the same time, the normal fluctuation of the energy Stack barrier of the exposure time and the heat capacity(mhu%) cumulative energy fluctuation curve that may have abnormal phenomena.

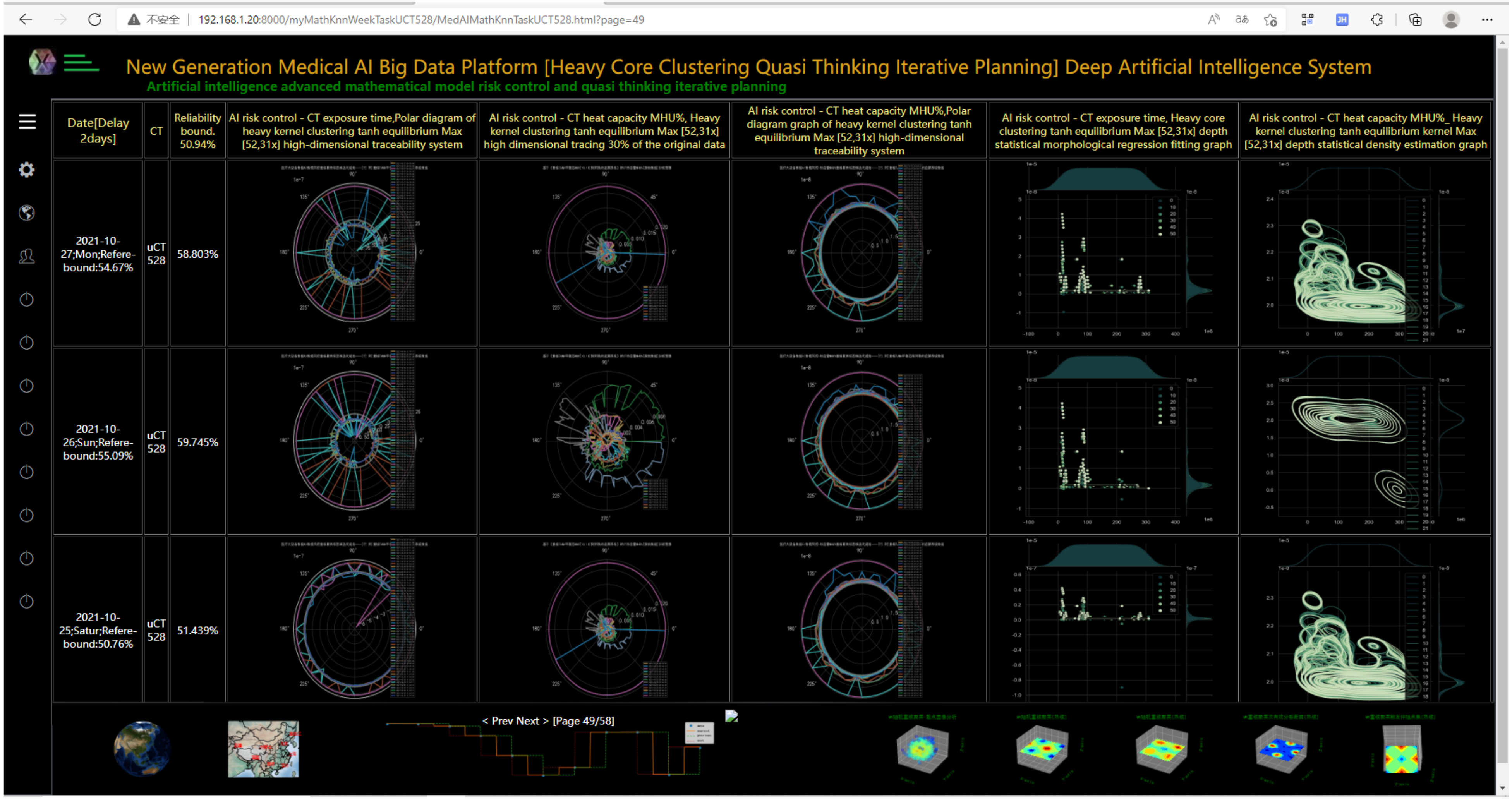

Supplementary Materials-B11.png

ⅲ. uCT528 has heavy core clustering lens [10,1]. Predictable maintenance of AI Mathematical model risk control found 3times machine repairs, and observes the following figure. **<u>1st machine repaired to uCT528, 2nd machine repaired to uCT528, 3rd machine repaired to uCT528.</u>**

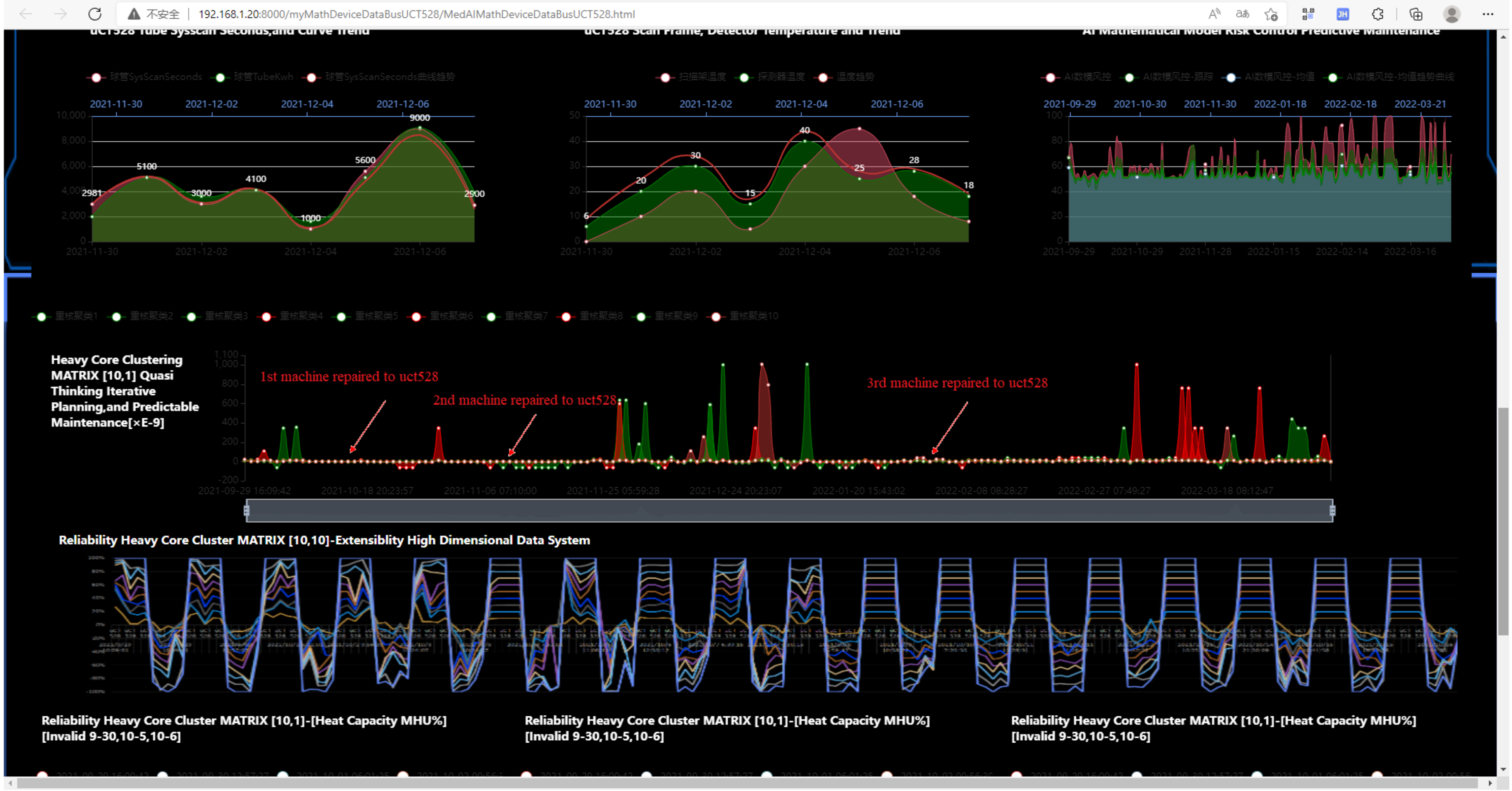

Supplement: Supplementary file 2 — Supplementary Information 2. [file 41598_2022_18724_MOESM2_ESM.pdf]
